# Application of Threonine Aldolases for the Asymmetric Synthesis of α‐Quaternary α‐Amino Acids

**DOI:** 10.1002/cctc.201800611

**Published:** 2018-07-04

**Authors:** Julia Blesl, Melanie Trobe, Felix Anderl, Rolf Breinbauer, Gernot A. Strohmeier, Kateryna Fesko

**Affiliations:** ^1^ Institute of Organic Chemistry Graz University of Technology Stremayrgasse 9, A- 8010 Graz Austria; ^2^ Austrian Centre of Industrial Biotechnology (ACIB) GmbH Petersgasse 14 8010 Graz Austria

**Keywords:** Amino acids, Asymmetric catalysis, Enzyme catalysis, Quaternary stereogenic centers, Threonine aldolases

## Abstract

We report the synthesis of diverse β‐hydroxy‐α,α‐dialkyl‐α‐amino acids with perfect stereoselectivity for the α‐quaternary center through the action of l‐ and d‐specific threonine aldolases. A wide variety of aliphatic and aromatic aldehydes were accepted by the enzymes and conversions up to >80 % were obtained. In the case of d‐selective threonine aldolase from *Pseudomonas* sp., generally higher diastereoselectivities were observed. The applicability of the protocol was demonstrated by performing enzymatic reactions on preparative scale. Using the d‐threonine aldolase from *Pseudomonas* sp., (2*R*,3*S*)‐2‐amino‐3‐(2‐fluorophenyl)‐3‐hydroxy‐2‐methylpropanoic acid was generated in preparative amounts in one step with a diastereomeric ratio >100 favoring the *syn*‐product. A Birch‐type reduction enabled the reductive removal of the β‐hydroxy group from (2*S*)‐2‐amino‐3‐hydroxy‐2‐methyl‐3‐phenylpropanoic acid to generate enantiopure l‐α‐methyl‐phenylalanine via a two‐step chemo‐enzymatic transformation.

## Introduction

Stereoselective syntheses of quaternary stereocenters are amongst the most challenging endeavors in organic chemistry.^1^ Apart from all‐carbon frameworks, heteroatoms are also present in assemblies of such structural elements. Several natural products such as sphingofungines E and F, lactacystin, as well as biologically‐active molecules for pharmaceutical applications contain α‐quaternary amino acids and, consequently, make these moieties attractive targets for the development of reliable synthetic strategies.^2^ Recently, α,α‐disubstituted amino acids were studied as building blocks for peptidomimetics to design novel short chain peptides useful for the treatment or prevention of diseases.^3^ The presence of α,α‐disubstituted amino acids in peptides generates specific conformational changes compared to their α‐hydrogen analogs and, in addition, their conformation tends to be more defined.^3^, ^4^ Among them, α‐methyl‐substituted amino acids such as α‐aminoisobutyric acid (Aib) or α‐methylphenylalanine ((αMe)Phe) are the most widely used candidates in peptide research.^5^ Moreover, peptides containing (αMe)Phe residues are promising sweeteners, chemoattractants, and candidates for molecular recognition studies. α‐Methyl‐DOPA is known as an antihypertensive drug, but has also found application as catalyst in asymmetric synthesis.^6^

The creation of α‐quaternary amino acids remains an often laborious task as protective steps need to be included and full control of the stereochemistry must be considered.^7^ The classical approach towards the asymmetric synthesis of α‐quaternary amino acids involves a diastereoselective alkylation of chiral lactim ethers or oxazolidinones.^8^ However, the application of these methods is limited due to substantial number of reaction steps involved. The asymmetric phase transfer‐catalyzed alkylation of intermediate Schiff bases, such as the one formed from benzaldehyde and alanine *tert*‐butyl ester, is a method to obtain α‐quaternary amino acids with *e.e*.’s up to 82 %.^9^ Enzymatic resolutions of chemically produced quaternary amino acid amides or amino acid esters using the amino amidase from *Mycobacterium neoaurum* ATCC 25795,^10^ respectively pig liver esterase,^11^ were reported for the synthesis of (*S*)‐α,α‐disubstituted amino acids with almost 100 % *e.e*. at 50 % maximal conversion.^12^

Biocatalytic methods for the direct formation of quaternary α‐amino acids are still scarce. Among those, aldolases proved to be efficient biocatalysts for the stereoselective carbon‐carbon bond formation,^13^ however, they are known for their strict specificity towards nucleophiles which limits the accessible product range. Recently, as one of few examples shown so far, the nucleophile specificity of d‐fructose‐6‐phosphate aldolase from *Escherichia coli* could be increased by enzyme engineering.^14^ Another prominent example where the nucleophile specificity was broadened was shown for the threonine aldolase type.^15^ For many years, threonine aldolases have been known for the asymmetric synthesis of α‐amino‐β‐hydroxy amino acids by coupling glycine with diverse sets of aldehydes with the aid of pyridoxal‐5′‐phosphate (PLP) as cofactor.^16^ Whereas the aldehyde scope is generally broad, the amino acid substrate of this enzyme class was strictly limited to glycine. In recent years, threonine aldolase enzymes able to accept alanine as a donor were found via screening and database mining^15^ or created via engineering of an alanine racemase^17^ or l‐serine hydroxymethyltransferase,^18^ and thus make these enzymes ideal catalysts for the direct synthesis of α‐quaternary α‐amino acids starting from aldehydes and amino acids. In addition, threonine aldolases are known to be highly stereospecific at the amino acids’ α‐carbon and the available enantiocomplementary forms provide access to l‐ and d‐amino acids with high stereoselectivity. Here, we present a more thorough investigation of threonine aldolases with broad donor specificity and show their applicability for the asymmetric synthesis of (*S*)‐ and (*R*)‐α,α‐disubstituted α‐amino acids by evaluating the substrate scope, reaction conditions and performing enzymatic reactions at larger scale. Additionally, methods to remove the β‐hydroxyl group in serine derivatives were investigated.

## Results and Discussion

**Figure 1:**
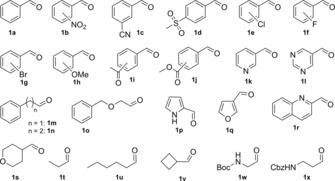
List of aldehydes used.

**Scheme 1:**
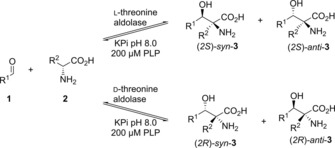
Biocatalytic synthesis of β‐hydroxy‐α,α‐dialkyl‐α‐amino acids using l‐ and d‐specific threonine aldolases.

To explore the synthetic potential of the wild‐type threonine aldolases with broad donor specificity, a panel of aldehydes (Figure 1) was screened in the aldol condensation reaction using d‐ or dl‐alanine in reactions with the l‐threonine aldolase from *Aeromonas jandaei* (l‐TA) and d‐threonine aldolase from *Pseudomonas* sp. (d‐TA) (Scheme 1, Table 1).

**Table 1:** *Syn*thesis of α,α‐disubstituted α‐amino acids with threonine aldolases using alanine.

Entry		**l‐TA**		**d‐TA**	
	**1**	*C* [%]^[a]^	*d.r*.^[b]^	*C* [%]^[a]^	*d.r*.^[b]^
1	**a**	25	1.7 (*syn*)	10	9.0 (*syn*)
2	**(*m*)‐b**	60	1.2 (*syn*)	40	9.0 (*syn*)
3	**c**	85	1.5 (*syn*)	58	18 (*syn*)
4	**d**	25	1.3 (*anti*)	<5	1.2 (*syn*)
5	**(*o*)‐e**	34	2.8 (*syn*)	13	>100 (*syn*)
6	**(*m*)‐e**	28	1.4 (*syn*)	8	>100 (*syn*)
7	**(*p*)‐e**	24	1.4 (*syn*)	<1	n. d.
8	**(*o*)‐f**	40	2.0 (*syn*)	29	>100 (*syn*)
9	**(*m*)‐f**	50	1.4 (*syn*)	18	>100 (*syn*)
10	**(*p*)‐f**	20	1.6 (*syn*)	2	n. d.
11	**(*o*)‐g**	4	1.5 (*syn*)	<1	n. d.
12	**(*m*)‐g**	5	1.5 (*syn*)	<1	n. d.
13	**(*p*)‐g**	15	1.4 (*syn*)	<1	n. d.
14	**(*o*)‐h**	<1	n. d.	‐	‐
15	**(*m*)‐h**	<1	n. d.	‐	‐
16	**(*p*)‐h**	<1	n. d.	‐	‐
17	**(*m*)‐i**	74	1.3 (*syn*)	8	5.1 (*syn*)
18	**(*p*)‐i**	74	1.2 (*syn*)	2	1.8 (*syn*)
19	**(*m*)‐j**	53	1.4 (*syn*)	2	11.5 (*syn*)
20	**(*p*)‐j**	44	1.1 (*syn*)	<1	n. d.
21	**k**	42	4.4 (*syn*)	45	1.5 (*syn*)
22	**l**	23	2.3 (*syn*)	38	1.6 (*syn*)
23	**m**	58	2.1 (*anti*)	37	2.8 (*syn*)
24	**n**	27	1.9 (*anti*)	14	2.6 (*syn*)
25	**o**	25	1.2 (*anti*)	63	1.2 (*anti*)
26	**p**	<1	n. d.	<1	n. d.
27	**q**	26	1.1 (*syn*)	38	1.9 (*anti*)
28	**r**	60	1.1 (*syn*)	‐	‐
29	**s**	27	1.3 (*anti*)	14	1.4 (*syn*)
30	**t**	60	1.2 (*anti*)	41	1.5 (*anti*)
31	**u**	58	1.2 (*anti*)	80	2.4 (*syn*)
32	**v**	44	1.9 (*syn*)	26	3.1 (*syn*)
33	**w**	41	1.3 (*anti*)	30	5.1 (*syn*)
34	**x**	30	1.3 (*anti*)	56	1.8 (*syn*)

[a] Conversion after 24 h at 30 °C in the presence of 50 mM aldehyde and 500 mM d‐alanine. [b] Diastereomeric ratio determined by rp‐HPLC after derivatization with *o*‐phthaldialdehyde and various thiol reagents and given as *syn*/*anti* ratio; *e.e*. >99.5 % (2*S* or 2*R*).

Forced by the often limited solubility of the aldehydes in water, different cosolvents were screened to study their compatibility with the aldolase‐catalyzed reactions (see Supporting Information). DMSO turned out as the best suited cosolvent and can be used in concentrations up to 20 % v/v in the enzymatic reactions. To compensate for the unfavorable reaction equilibrium in the aldol addition, a 10‐fold excess of a donor was used.

In view of the α‐methyl‐phenylalanine motifs used for studying peptide‐peptide or peptide‐membrane interactions,5a we have tested a range of substituted benzaldehydes as electrophiles in enzymatic aldol condensations with d‐alanine to evaluate the herein described method for the generation of α‐quaternary amino acids (Scheme 1). In all cases, only a single enantiomer of the product amino acid was obtained, which is strictly controlled by the type of enzyme used (e. g. *e.e._S_* >99 % for l‐TA, *e.e._R_* >99 % for d‐TA). Superior conversions were obtained with benzaldehydes bearing electron withdrawing groups, such as nitro (**1 b**), cyano (**1 c**), or acetyl (**1 i**), because of the increased electrophilic reactivity. In general, the conversion and diastereoselectivity varies dependent on the nature of the substituent and its position at the aromatic ring.

Interestingly, *meta*‐substituted benzaldehydes were often better converted with l‐TA, whereas the reactions with *ortho*‐substituted benzaldehydes usually gave higher conversions in the presence of d‐TA. Using 3‐nitrobenzaldehyde (**1 b**), a conversion up to 60 % and mixture of (*S*)‐*syn*‐ and (*S*)‐*anti*‐diastereomers with a *d.r*._*syn* _of 1.2 was obtained with l‐TA. Under the same conditions using d‐TA as catalyst, the corresponding (*R*)‐product was produced with a lower conversion of 40 % but in significantly higher *d.r._syn_* of 9 (Table 1, Entry 2). Other excellent acceptors in the aldol addition are halogenated aromatic aldehydes. Chloro‐ (**1 e**) and fluorobenzaldehydes (**1 f**) were well accepted by l‐ and d‐TA with d‐alanine as a donor (Table 1, Entries 5–10). However, the bromobenzaldehydes (**1 g**) were significantly lower converted by the enzymes, presumably because of the larger size of the halogen atom which causes increased steric hindrance. The aromatic aldehydes bearing a ketone (**1 i**) or a methyl ester function (**1 j**) were converted by l‐TA with up to 74 % and 53 % conversion, respectively. In contrast, the reactions with d‐TA gave conversions below 15 % (Table 1, Entries 17–20). Increasing the length of aliphatic chains between the aromatic ring and carbonyl function (e. g. aldehydes **1 m**, **1 n**, **1 o**) did not much influence the conversion in the l‐TA catalyzed reaction, but is beneficial in the case of d‐TA. Bulky substrates are well accepted by the investigated threonine aldolases and inverted stereopreference at the product β‐carbon was obtained in the case of l‐TA (Table 1, Entries 23–25).

Interestingly, the enzymes showed particularly high activity towards heterocyclic aldehydes, e. g. **1 k**, **1 l**, **1 q**, **1 r** and **1 s**, yielding α‐quaternary α‐amino acids with heterocyclic side chains, which are important motifs in many biologically relevant compounds. Pyridine‐3‐carbaldehyde (**1 k**) and quinoline‐2‐carbaldehyde (**1 r**) were converted into the corresponding amino acids with conversions up to 60 % for l‐TA and slightly lower conversion for d‐TA (Table 1, Entries 21 and 28). The aliphatic aldehydes **1 t**–**1 x** were also preferred substrates for the tested l‐TA and d‐TA (Table 1, Entries 30–34). Conversions up to 56 % was achieved with *N*‐Boc‐2‐aminoacetaldehyde (**1 w**) and 3‐[(carbobenzyloxy)amino]propionaldehyde (**1 x**) as acceptors and d‐alanine as donor with both l‐TA and d‐TA (Table 1, Entries 33 and 34). As a wide variety of aliphatic, aromatic and heterocyclic aldehydes was accepted by the enzymes, non‐natural amino acids with diverse side chains and different functional groups can be accessed.

**Figure 2:**
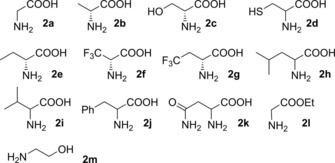
List of amino acids and other donors investigated.

Among all nucleophiles tested (Figure 2), both enzymes best tolerate glycine (**2 a**), d‐alanine (**2 b**), d‐serine (**2 c**), and d‐cysteine (**2 d**) as amino acid donors in most enzyme‐aldehyde combinations. More sterically demanding or electronically different candidates including **2 e**–**k**, ethyl glycinate (**2 l**) or 2‐aminoethanol (**2 m**) are not converted or converted to a little extent only (Table 2).

**Table 2:** Investigation of amino acid donor specificities.

Entry			l‐TA		d‐TA	
	**1** ^[a]^	**2** ^[a]^	*C* [%]^[a]^	*d.r*.^[a,b]^	*C* [%]^[a]^	*d.r*.^[a,b]^
1	**a**	**a**	24	3.2 (*syn*)	14	1.2 (*anti*)
2	**a**	**b**	35	1.1 (*syn*)	10	9.0 (*syn*)
3	**a**	**c**	10	3.2 (*anti*)	<1	n. d.
4	**a**	**d**	27	1.4 (*anti*)	33^[c]^	1.5 (*anti*)^[c]^
5	**a**	**e**	2	n.d.	1	n.d.
6	**a**	**f**	7	n.d.	10^[c]^	n.d.
7–13	**a**	**g**–**m**	0	–	0	–
14	**(*m*)‐b**	**a**	50	3.7 (*syn*)	25	1.7 (*syn*)
15	**(*m*)‐b**	**b**	60	1.2 (*syn*)	36	7.3 (*syn*)
16	**(*m*)‐b**	**c**	15	4.7 (*anti*)	5	1.6 (*anti*)
17	**(*m*)‐b**	**d**	30	1.3 (*syn*)	39^[c]^	1.1 (*syn*)^[c]^
18	**(*m*)‐b**	**e**	2	5.1 (*syn*)	4	2.3 (*syn*)
19	**(*m*)‐b**	**f**	<10	n.d.	<10^[c]^	n.d.
20‐26	**(*m*)‐b**	**g**–**m**	0	–	0	–

[a] Conversion after 24 h at 30 °C in the presence of 50 mM aldehyde and 500 mM **2 a‐m**. [b] Determined by rp‐HPLC after derivatization with *o*‐phthaldialdehyde and various thiol reagents and given as *syn*/*anti* ratio;.*e.e*. >99.5 % (2*S* or 2*R*). [c] Determined by ^18^F NMR.

To demonstrate the applicability of the described asymmetric biocatalytic synthesis of β‐hydroxy α‐quaternary amino acids, selected reactions were performed at preparative scale.^19^ Most reaction products were isolated with typically 10–30 % yield and perfect enantioselectivity using 100–250 mM concentrations of the aldehydes (Table 3), demonstrating a straightforward one‐step synthesis of α,α‐disubstituted non‐natural amino acids. At increasing aldehyde concentration, some inactivation of the enzymes was observed, and thus continuous feeding of the acceptor substrate turned out to be advantageous. Alternatively, for tackling substrate inhibition in threonine aldolase catalyzed reactions, slug‐flow microfluidic system could be used.^20^ In general, diastereomeric mixtures of amino acid products with moderate excess of the *syn*‐isomers were isolated from l‐TA‐catalyzed reactions. The diastereomers can be typically separated by flash‐chromatography on reversed‐phase silica gel. A single (*R*)‐*syn*‐diastereomer was isolated from the reaction with 3‐fluoro‐benzaldehyde (**(*m*)‐1 f**) and dl‐alanine catalyzed by d‐TA (Table 3, Entry 8).

**Table 3:** Preparative scale synthesis of α,α‐dialkyl α‐amino acids with threonine aldolases.

Entry	**1** ^[a]^	Enzyme^[b]^	Yield [%]^[c]^	*d.r*. [%]^[c,d]^
1	**a**	l‐TA	13	2.2 (*syn*)
2	**a**	d‐TA	12	7.8 (*syn*)
3	**(*o*)‐b**	l‐TA	14	2.3 (*syn*)
4	**(*m*)‐b**	l‐TA	14	1.6 (*syn*)
5	**(*p*)‐b**	l‐TA	7	1.2 (*syn*)
6	**(*o*)‐e**	l‐TA	16	2.5 (*syn*)
7	**(*o*)‐f**	l‐TA	20	3.5 (*syn*)
8	**(*m*)‐f**	d‐TA	27	>100 (*syn*)
9	**(*m*)‐i**	l‐TA	21	1.1 (*syn*)
10	**(*p*)‐i**	l‐TA	22	1.2 (*syn*)
11	**(*m*)‐j**	l‐TA	28	1.1 (*anti*)
12	**k**	l‐TA	23	2.2 (*syn*)
13	**r**	l‐TA	4	1.4 (*syn*)
14	**u**	l‐TA	10	1.25 (*anti*)
15	**u**	d‐TA	27	2.7 (*syn*)
16	**x**	d‐TA	32	57 (*syn*)

[a] 100–250 mM aldehyde, 1.0 or 1.5 M dl‐alanine used [b] l‐TA from *Aeromonas jandaei*, respectively d‐TA from *Pseudomonas* sp. used. [c] After reaction times of 2–7 d at 30 °C; isolated yield after chromatography. [d] Diastereomeric ratio determined by ^1^H NMR.

While the stereoselectivity at α‐quaternary center is stringently controlled by the applied enzymes, the selectivity at the β‐carbon (C_β_) forming the hydroxyl group is moderate and depends on the nature of the substrate aldehyde. In l‐TA‐catalyzed reactions with aromatic substrates, the *syn*‐products were preferably obtained, whereas with long‐chain aliphatic aldehydes or with increasing distance between the aromatic ring and the aldehyde group, the formation of *anti*‐products is favored. A plausible reason for the low diastereoselectivity was provided recently by pointing at the function of two histidine residues in the active site (His85 and His128′ for l‐TA from *Aeromonas jandaei*), which both can form hydrogen bonding with the C_β_‐oxygen from opposite sides.^21^ Until now, attempts to alter the stereoselectivity at the β‐carbon by enzyme engineering have failed mainly due to complex hydrogen bonding network in the active site participating in the protonation step.^22^ On the other hand, d‐specific TA usually give higher *d.r*.’s, which was explained by a slow thermodynamic equilibration step between *syn*‐ and *anti*‐isomers in contrast to the analogous reactions with the l‐TA.^23^ But this is only true for the β‐phenylserine derivatives, where a *d.r*. up to >100 can be reached with the d‐TA.16j

The initially formed aromatic β‐serine derivatives can be reductively de‐hydroxylated in order to obtain optically pure α‐tetrasubstituted amino acids. To demonstrate this, we have performed a Birch‐type reduction starting from a *syn*/*anti* diastereomeric mixture of (2*S*)‐2‐amino‐3‐hydroxy‐2‐methyl‐3‐phenylpropanoic acid (**3 a**), which had been obtained from an l‐TA‐catalyzed reaction of benzaldehyde **1 a** and dl‐alanine **2 b**. The Birch‐reduction is a particularly useful transformation for the reduction of aromatic compounds using group I metals, commonly sodium or lithium, in liquid ammonia and in the presence of weak acids which act as proton source.^24^ The advantage of this method is that unprotected amino acids can be directly used in the reduction reaction.^25^

**Scheme 2:**
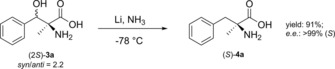
β‐Dehydroxylation of (2*S*)‐2‐amino‐3‐hydroxy‐2‐methyl‐3‐phenylpropanoic acid **3 a** via Birch reduction.

The corresponding (*S*)‐α‐methylphenylalanine **4 a** was isolated in 91 % yield and an unchanged high optical purity of >99 % (Scheme 2). Analogously, the (*R*)‐isomer can be obtained from (2*R*)‐2‐amino‐3‐hydroxy‐2‐methyl‐3‐phenylpropanoic acid **3 a**, synthesized by d‐TA. Thus, (*S*)‐α‐methylphenylalanine, a valuable non‐natural amino acid, which is used in peptidomimetics and as sweetener, was produced using a simple two‐step chemo‐enzymatic synthesis starting from inexpensive benzaldehyde and alanine in an overall yield of 10 % and an *e.e*. >99 %.

**Scheme 3:**
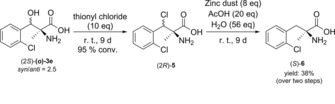
β‐Dehydroxylation of (2*S*)‐2‐amino‐3‐hydroxy‐2‐methyl‐3‐(2‐chlorophenyl)propanoic acid ***(o)***‐**3 e**.

As the conditions of the Birch‐reductions are not compatible with several functional groups (see SI for some examples), we investigated an alternative method of dehydroxylation which involves first the conversion of the β‐OH into a benzylic chlorine substituent followed by mild dehalogenation with Zn/AcOH.^26^ The application of this method for the removal of hydroxyl‐group from a *syn/anti* diastereomeric mixture of (2S)‐**(*o*)‐3 e** led to the (2S)‐2‐amino‐2‐methyl‐3‐(2‐chlorophenyl)propanoic acid (S)‐**6** with 38 % yield and *e.e*. >99 % (Scheme 3).

## Conclusions

In summary, threonine aldolases show a broad substrate scope for the preparation of β‐hydroxy α‐quaternary α‐amino acids with various structural features. In the aldol addition, two new stereogenic centers are formed with perfect enantiospecifity at the α‐carbon (> 99 % *e.e*.). Moreover, a strategy for the removal of the β‐hydroxy‐group gives access to quaternary α‐amino acids with perfect stereoselectivity. The herein described biocatalytic method provides a fast and green access towards enantiopure non‐natural quaternary amino acids in preparative amounts. Although some wild‐type threonine aldolases have the unique property to accept few d‐amino acids as donor, further sequence search or enzyme engineering based on directed evolution or rational design may lead to the development of catalysts with broader donor specificity, as recently shown for other aldolases. Combining threonine aldolases in cascades with other reactions can be beneficial to overcome the intrinsic limitations of the reaction equilibrium and, consequently, could lead to improved conversions.

## **Experimental Section**

**Procedure for the synthesis of (2*S*)‐2‐amino‐3‐(2‐chlorophenyl)‐3‐hydroxy‐2‐methylpropanoic acid) ((2*S*)‐3 e, Table** 3 **, entry 6)**

In a 500 mL round‐bottom flask, dl‐alanine (19.39 g, 218 mmol, 10.0 eq) was dissolved in 50 mM potassium phosphate buffer pH 8.0 (168 mL) and 2‐propanol (4.0 mL). Under stirring, PLP (6.0 mg) was added. The yellow solution was stirred at 25 °C for 30 min. Then l‐TA (5.0 mL, 1550 units) was added. 2‐Chlorobenzaldehyde (3.10 g, 21.8 mmol, 1.0 eq) was dissolved in 2‐propanol (18 mL, final concentration of 2‐propanol in the reaction: 10 vol%) and added under slow stirring via a syringe pump to the reaction over 24 h. After 24 h, further l‐TA (5 mL, 1550 units) was added and stirring continued another 2 d at 25 °C. Then formic acid (1 mL) was added and the yellowish, cloudy suspension heated to 80 °C for 30 min to inactivate the enzyme. After cooling to room temperature, the amino acid product remained soluble under these conditions. Insoluble materials were filtered off and the filtrate extracted with ethyl acetate (1×100 mL) to remove unconverted aldehyde prior to the chromatography. The aqueous phase was concentrated *in vacuo* to about 80 mL which were then directly applied on the column. Via flash chromatography (60 g silica gel 60 C18, 35–70 μm, Carl Roth, Karlsruhe, Germany, prod. no. 5504; column size: 11×3 cm) using a stepwise gradient starting with H_2_O/methanol 99 : 1+0.1 vol% formic acid up to 85 : 15, the product was isolated. All fractions containing the pure product were pooled and concentrated. Yield: 807 mg (3.51 mmol, 16 %) of colorless solid. e.e. >99.5 (2*S*), d.r.=2.5 ((2*S*,3*R*)/(2*S*,3*S*)). Mp=195–212 °C (dec.). [α]_D_^25^=−9.1 (*c*=0.1 in 1 M HCl). ^1^H‐NMR (300.36 MHz, D_2_O): δ=7.61–7.34 (m, 4H, H_Ar_), 5.73 (s, 1H, −CHOH, *syn*), 5.47 (s, 1H, −CHOH, *anti*), 1.54 (s, 1H, −CCH_3_NH_2_, *anti*), 1.35 (s, 1H, −CCH_3_NH_2_, *syn*). ^13^C‐NMR (75.53 MHz, D_2_O): δ=175.1 (C_q,_ −COOH), 173.4 (C_q,_ −COOH), 135.5 (C_q_, C_Ar_), 135.4 (C_q_, C_Ar_), 133.3 (C_q_, −CCl), 132.7 (C_q_, −CCl), 130.2 (C_Ar_), 130.1 (C_Ar_), 129.8 (C_Ar_), 129.6 (C_Ar_), 129.1 (C_Ar_), 128.4 (C_Ar_), 127.6 (C_Ar_), 127.1 (C_Ar_), 71.3 (−CHOH), 70.3 (−CHOH), 65.7 (C_q_, −CCH_3_NH_2_), 65.1 (C_q_, −CCH_3_NH_2_), 19.2 (−CCH_3_NH_2_), 18.3 (−CCH_3_NH_2_). HRMS (MALDI‐TOF): Calcd. for C_10_H_12_ClNO_3_H [M+H]^+^: 230.0584; found: 230.0585.

## Conflict of interest

The authors declare no conflict of interest.

## Supporting information

As a service to our authors and readers, this journal provides supporting information supplied by the authors. Such materials are peer reviewed and may be re‐organized for online delivery, but are not copy‐edited or typeset. Technical support issues arising from supporting information (other than missing files) should be addressed to the authors.

Supplementary

## References

[1] K. W. Quasdorf , L. E. Overman , Nature 2014, 516, 181–191. 25503231 10.1038/nature14007 PMC4697831

[2] S. H. Kang , S. Y. Kang , H.-S. Lee , A. J. Buglass , Chem. Rev. 2005, 105, 4537–4558. 16351053 10.1021/cr040608g

[3] K. A. Brun , A. Linden , H. Heimgartner , Helv. Chim. Acta 2008, 91, 526–558.

[4] M. Crisma , C. Peggion , A. Moretto , R. Banerjee , S. Supakar , F. Formaggio , C. Toniolo , Pept. Sci. 2014, 102, 145–158. 10.1002/bip.22450 24307568

[5a] M. De Zotti , S. Bobone , A. Bortolotti , E. Longo , B. Biondi , C. Peggion , F. Formaggio , C. Toniolo , A. Dalla Bona , B. Kaptein , L. Stella , Chem. Biodiversity 2015, 12, 513–527; 10.1002/cbdv.201400404 25879497

[5b] C. Toniolo , F. Formaggio , M. Crisma , G. Valle , W. H. J. Boesten , H. E. Schoemaker , J. Kamphuis , P. A. Temussi , E. L. Becker , G. Précigoux , Tetrahedron 1993, 49, 3641–3653.

[6] E. R. Parmee , O. Tempkin , S. Masamune , A. Abiko , J. Am. Chem. Soc. 1991, 113, 9365–9366.

[7a] C. Cativiela , M. D. Díaz-de-Villegas , Tetrahedron: Asymmetry 2007, 18, 569–623;

[7b] T. Wirth , Angew. Chem. Int. Ed. Engl. 1997, 36, 225–227.

[8a] M. Gander-Coquoz , D. Seebach , Helv. Chim. Acta 1988, 71, 224–236;

[8b] U. Schöllkopf , R. Lonsky , P. Lehr , Liebigs Ann. Chem. 1985, 1985, 413–417.

[9] Y. N. Belokon , K. A. Kochetkov , T. D. Churkina , N. S. Ikonnikov , A. A. Chesnokov , O. V. Larionov , V. S. Parmár , R. Kumar , H. B. Kagan , Tetrahedron: Asymmetry 1998, 9, 851–857.

[10] W. H. Kruizinga , J. Bolster , R. M. Kellogg , J. Kamphuis , W. H. J. Boesten , E. M. Meijer , H. E. Schoemaker , J. Org. Chem. 1988, 53, 1826–1827.

[11] B. Kaptein , W. H. J. Boesten , Q. B. Broxterman , P. J. H. Peters , H. E. Schoemaker , J. Kamphuis , Tetrahedron: Asymmetry 1993, 4, 1113–1116.

[12] T. Sonke , B. Kaptein , W. H. J. Boesten , Q. B. Broxterman , H. E. Schoemaker , J. Kamphuis , F. Formaggio , C. Toniolo , F. P. J. T. Rutjes , in Stereoselective Biocatalysis (Ed.: R. N. Patel), Marcel Dekker, Inc., New York, 2000, pp. 23–58.

[13] K. Fesko , M. Gruber-Khadjawi , ChemCatChem 2013, 5, 1248–1272.

[14] A. Szekrenyi , X. Garrabou , T. Parella , J. Joglar , J. Bujons , P. Clapés , Nat. Chem. 2015, 7, 724–729. 26291944 10.1038/nchem.2321

[15a] K. Fesko , G. A. Strohmeier , R. Breinbauer , Appl. Microbiol. Biotechnol. 2015, 99, 9651–9661; 26189018 10.1007/s00253-015-6803-y

[15b] K. Fesko , M. Uhl , J. Steinreiber , K. Gruber , H. Griengl , Angew. Chem. Int. Ed. 2010, 49, 121–124; 10.1002/anie.200904395 19943295

[16] For selected references on the application of threonine aldolases, see the following:

[16a] Q. Chen , X. Chen , Y. Cui , J. Ren , W. Lu , J. Feng , Q. Wu , D. Zhu , Catal. Sci. Technol. 2017, 7, 5964–5973;

[16b] Y. Hirato , M. Tokuhisa , M. Tanigawa , H. Ashida , H. Tanaka , K. Nishimura , Phytochemistry 2017, 135, 18–23; 28038776 10.1016/j.phytochem.2016.12.012

[16c] S. E. Franz , J. D. Stewart , in Advances in Applied Microbiology, Vol. 88 (Eds.: S. Sariaslani, G. M. Gadd), Academic Press, 2014, pp. 57–101; 10.1016/B978-0-12-800260-5.00003-6 24767426

[16d] F. Sagui , C. De Micheli , G. Roda , P. Magrone , R. Pizzoli , S. Riva , J. Mol. Catal. B 2012, 75, 27–34;

[16e] K. Baer , N. Dückers , T. Rosenbaum , C. Leggewie , S. Simon , M. Kraußer , S. Oßwald , W. Hummel , H. Gröger , Tetrahedron: Asymmetry 2011, 22, 925–928;

[16f] H.-J. Gwon , S.-H. Baik , Biotechnol. Lett. 2009, 32, 143; 19760118 10.1007/s10529-009-0125-z

[16g] M. L. Gutierrez , X. Garrabou , E. Agosta , S. Servi , T. Parella , J. Joglar , P. Clapés , Chem. Eur. J. 2008, 14, 4647–4656; 18384024 10.1002/chem.200800031

[16h] F. Sagui , P. Conti , G. Roda , R. Contestabile , S. Riva , Tetrahedron 2008, 64, 5079–5084;

[16i] J. Steinreiber , K. Fesko , C. Mayer , C. Reisinger , M. Schürmann , H. Griengl , Tetrahedron 2007, 63, 8088–8093;

[16j] J. Steinreiber , K. Fesko , C. Reisinger , M. Schürmann , F. van Assema , M. Wolberg , D. Mink , H. Griengl , Tetrahedron 2007, 63, 918–926; 10.1002/anie.200604142 17397072

[16k] J. Q. Liu , M. Odani , T. Yasuoka , T. Dairi , N. Itoh , M. Kataoka , S. Shimizu , H. Yamada , Appl. Microbiol. Biotechnol. 2000, 54, 44–51; 10952004 10.1007/s002539900301

[16l] K. Nishide , K. Shibata , T. Fujita , T. Kajimoto , C.-H. Wong , M. Node , Heterocycles 2000, 52, 1191–1201;

[16m] T. Kimura , V. P. Vassilev , G.-J. Shen , C.-H. Wong , J. Am. Chem. Soc. 1997, 119, 11734–11742.

[17] K. Fesko , L. Giger , D. Hilvert , Bioorg. Med. Chem. Lett. 2008, 18, 5987–5990. 18760921 10.1016/j.bmcl.2008.08.031

[18] K. Hernandez , I. Zelen , G. Petrillo , I. Usón , C. M. Wandtke , J. Bujons , J. Joglar , T. Parella , P. Clapés , Angew. Chem. Int. Ed. 2015, 54, 3013–3017; 10.1002/anie.201411484 25611820

[19] For a kg scale synthesis of (2*R*,3*S*)-2-amino-3-hydroxy-3-(pyridin-4-yl)-propanoic acid using an engineered d-threonine aldolase, see the following: S. L. Goldberg , A. Goswami , Z. Guo , Y. Chan , E. T. Lo , A. Lee , V. C. Truc , K. J. Natalie , C. Hang , L. T. Rossano , M. A. Schmidt , Org. Process Res. Dev. 2015, 19, 1308–1316.

[20] J. Čech , V. Hessel , M. Přibyl , Chem. Eng. Sci. 2017, 169, 97–105.

[21a] H.-M. Qin , F. L. Imai , T. Miyakawa , M. Kataoka , N. Kitamura , N. Urano , K. Mori , H. Kawabata , M. Okai , J. Ohtsuka , F. Hou , K. Nagata , S. Shimizu , M. Tanokura , Acta Crystallogr. Sect. D 2014, 70, 1695–1703; 24914980 10.1107/S1399004714007664

[21b] M. L. di Salvo , S. G. Remesh , M. Vivoli , M. S. Ghatge , A. Paiardini , S. D′Aguanno , M. K. Safo , R. Contestabile , FEBS J. 2014, 281, 129–145. 24165453 10.1111/febs.12581 PMC4366684

[22] K. Fesko , Appl. Microbiol. Biotechnol. 2016, 100, 2579–2590. 26810201 10.1007/s00253-015-7218-5 PMC4761611

[23] K. Fesko , C. Reisinger , J. Steinreiber , H. Weber , M. Schürmann , H. Griengl , J. Mol. Catal. B 2008, 52–53, 19–26.

[24] P. W. Rabideau , Z. Marcinow , in Organic Reactions, Vol. 42 (Ed.: L. A. Paquette), John Wiley & Sons, Inc., Hoboken, NJ, 1992, pp. 1–334.

[25] For Birch-type dehydroxylations of 2-amino-1-phenylethanol-like structure, see the following:

[25a] V. Kunalan , W. J. Kerr , N. NicDaéid , Forensic Sci. Int. 2012, 223, 321–329; 23127659 10.1016/j.forsciint.2012.10.008

[25b] P.-P. Ilich , K. R. McCormick , A. D. Atkins , G. J. Mell , T. J. Flaherty , M. J. Bruck , H. A. Goodrich , A. L. Hefel , N. Juranić , S. Seleem , J. Chem. Educ. 2010, 87, 419–422.

[26] M. Peters , M. Trobe , R. Breinbauer , Chem. Eur. J. 2013, 19, 2450–2456. 23281154 10.1002/chem.201203006

