# Supplementary material for: Application of Threonine Aldolases for the Asymmetric Synthesis of α‐Quaternary α‐Amino Acids
Source: ChemCatChem. 2018 Jul 4;10(16):3453–8. doi: 10.1002/cctc.201800611 (PMC6485451; doi:10.1002/cctc.201800611)

Supporting Information

© Copyright Wiley-VCH Verlag GmbH & Co. KGaA, 69451 Weinheim, 2018

Application of Threonine Aldolases for the Asymmetric Synthesis of α-Quaternary α-Amino Acids

Julia Blesl, Melanie Trobe, Felix Anderl, Rolf Breinbauer, Gernot A. Strohmeier, and Kateryna Fesko*©2018 The Authors. Published by Wiley-VCH Verlag GmbH & Co. KGaA. This is an open access article under the terms of the Creative Commons Attribution License, which permits use, distribution and reproduction in any medium, provided the original work is properly cited.

Table of contents

1. Experimental 2
   1.1. General 2
   1.2. Monitoring of reactions and analysis of compounds 3
   1.3. Preparation of threonine aldolases 6
   1.4. Screening of the reaction conditions 7
      1.4.1. Determination of the pH optimum in threonine aldolase-catalyzed reactions 7
      1.4.2. Investigation of the influence of co-solvents on the threonine aldolase-catalyzed reactions 7
      1.4.3. Influence of divalent manganese ions on the D-TA-catalyzed reactions 8
   1.5. Experimental procedure for threonine aldolase catalyzed reactions 8
      1.5.1. General procedure for the substrate screening in the aldol reactions catalyzed by L-TA and D-TA 8
      1.5.2. Biocatalytic syntheses of serine derivatives at preparative scale 9
   1.6. Additional syntheses and related experimental procedures 26
      1.6.1. Birch reductions of phenylserine derivatives 26
      1.6.2. Synthesis of (S)- and (R)-2-Amino-3-(2-chlorophenyl)-2-methylpropanoic acid 28
2. Literature 29
3. NMR spectra 30

1. Experimental

1.1. General

All experiments were carried out under air, unless noted otherwise. Inert reactions were carried out using standard Schlenk techniques under an inert atmosphere of argon or nitrogen. Before use, the glass apparatuses were dried under oil pump vacuum while heating with a heat-gun and then cooled to room temperature and flushed with inert gas. In non-enzymatic reactions, reagents and solvents were always added under an inert gas counter-stream. Enzyme-catalyzed reactions were run up to 1.5 mL scale under thermostatted conditions in Eppendorf Thermomixers comfort and at larger volumes in an incubation shaker (Infors AG, Bottmingen, Switzerland) or in glass vessels under magnetic stirring and temperature control. All chemicals and reagents were purchased from Acros Organics, Sigma Aldrich, Fluka, Merck KGaA, Riedel-de Haën, Carl Roth and VWR and were used without further purification unless otherwise mentioned. Ammonia used for chromatography purposes was a 14 M aqueous solution (25 weight% NH_3, ammonium hydroxide). Flash column chromatography was performed on silica gel 0.035-0.070 mm, 60 Å (product no. 240360300; Acros Organics, Geel, Belgium) or on reversed-phase silica gel 60 C18 0.035-0.070 mm (product no. 5504.3; Carl Roth, Karlsruhe, Germany). ^{1}H and ^{13}C NMR spectra were recorded on a Bruker AVANCE III 300 spectrometer (^{1}H: 300.36 MHz; ^{13}C: 75.53 MHz) and chemical shifts are referenced to residual protonated solvent signals as internal standard. HRMS spectra were recorded with MALDI-TOF mass spectrometry on a Micromass TofSpec 2E Time-of-Flight Mass Spectrometer. HPLC analysis was conducted on a Shimadzu Nexera instrument, equipped with a LCMS-2020 quadrupol mass spectrometer and SPD-M20A Prominence Diode Array Detector, an Agilent 1200 instrument, equipped with a 6120 quadrupol mass spectrometer, or on an Agilent 1100 Series HPLC system equipped with a diode array detector. The specific optical rotation was determined on a Perkin Elmer Polarimeter 341 with an integrated sodium vapor lamp. All samples were measured at the D-line of the sodium light (λ = 589 nm).

1.2. Monitoring of reactions and analysis of compounds

High-performance liquid chromatography (HPLC)

For general monitoring of the reactions, eluent mixtures consisting of methanol and water containing 0.01 vol% formic acid as additive were used. UV (210 nm) and ESI-MS detection, typically in positive mode, was used to identify the compounds.

Analyses of amino acids:

Analytical HPLC-MS measurements were performed on a Shimadzu Nexera LCMS-2020 system (method_1 and 2) or on an Agilent 1200 Series RR-HPLC system (method_3-5). The substances were detected at a wavelength of $\lambda = 210$ nm with the LCMS-2020 Liquid Chromatograph Mass Spectrometer or with the 6120 mass selective detector in ESI mode at positive and/or negative polarity. Following settings of the Agilent 6120 MSD were applied: drying gas: N_2, 10.5 L/min; drying gas temperature: 350°C; nebulizer pressure: 35 psi; capillary voltage: 3000 V; mode: negative; scan parameters: mass range: 100-450, fragmentor: 30 V, gain: 1.0, threshold: 150, step size: 0.10.

The separation of the analytes was carried out using a Poroshell® 120 EC-C18, 3.0 × 100 mm, 2.7 µm (Agilent, Vienna, Austria) reversed-phase column. Samples were either dissolved in acetonitrile or methanol and any insoluble particles were removed prior to analysis by centrifugation or filtration through 0.2 µm GHP Acrodic® 13 mm syringe filters (PN 4567, Pall Corporation, Ann Arbor, USA). The following methods were used for the separations:

method_1: 0.0 - 0.5 min 98 % water/ 0.01 % HCOOH and 2 % CH_3CN, 0.5 – 3.5 min linear gradient to 32 % CH_3CN, 3.5 – 4.5 min linear gradient to 100 % CH_3CN, 4.5 – 5.5 min 100 % CH_3CN; 0.7 mL/min, 40 °C.

method_2: 0.0 min: 98 % water/ 0.1 % HCOOH and 2 % CH_3OH, linear gradient to 100 % CH_3OH in 6 min, 6.0 – 8.0 min 100 % CH_3OH; 0.7 mL/min, 30 °C.

method_3: 0.0 – 6.0 min 98 % water/ 0.1 % HCOOH and 2 % CH_3OH, linear gradient to 32 % CH_3OH, 6.0 – 8.0 min linear gradient to 100 % CH_3OH, 8.0 – 10.0 min 100 % CH_3OH; 0.7 mL/min, 30 °C.

method_4: 0.0 – 4.0 min 98 % water/ 0.1 % HCOOH and 2 % CH_3OH, 4.0 – 6.0 min linear gradient to 30 % CH_3OH, 6.0 – 8.0 min linear gradient to 100 % CH_3OH; 0.7 mL/min, 30 °C.

method_5: 0.0 – 6.0 min 98 % water/ 0.1 % HCOOH and 2 % CH_3CN, linear gradient to 100 % CH_3OH, 6.0 – 8.0 min 100 % CH_3CN; 0.7 mL/min, 30 °C.

Reversed-phase preparative HPLC purifications were run on a Thermo Scientific UltiMate 3000 semi-preparative system. Detection was accomplished with a Dionex UltiMate MWD-3000. The separations were carried out on a 125/21 Nucleodur® 100-5 C18EC (125 × 21 mm, 5.0 µm; Macherey-Nagel, Düren, Germany) column. The following method was used:
method_6: 0.0 – 30.0 min 98 % 0.025 % HCOOH and 2 % CH_3OH, linear gradient to 52 % CH_3OH, 30.0 – 33.0 min 48 % 0.025 % HCOOH and 52 % CH_3OH, 33.0 – 34.0 min linear gradient to 100 % CH_3OH, 34 – 40 min 100 % CH_3OH; 19 mL/min, 30 °C.

Formation of isoindole derivatives of amino acids for the HPLC analyses:

The analyses of the isoindol-derivatized amino acids (after reaction with *ortho*-phthaldialdehyde (OPA) and a thiol in the presence of base) were performed on an Agilent 1100 Series HPLC system.[1] Detection of the analytes was accomplished with a diode array detector at a wavelength of λ = 210 nm and λ = 340 nm, respectively. The following methods were used for the separations:
method_7: The separations were carried out on an EC 150/3 Nucleodur® C18 Gravity column (150 × 3.0 mm, 3.0 µm; Macherey-Nagel, Düren, Germany). 0.0 min: 85 % 50 mM KOAc (pH = 6.4) and 15 % CH_3CN, linear increase to 35 % CH_3CN, 11.0 – 12.0 min 20 % 50 mM KOAc (pH = 6.4) and 80 % CH_3CN; 0.65 mL/min, 30 °C.
method_8: The separations were carried out on a Chromolith® Performance RP-18e column (100 × 4.6 mm, 2.0 µm; Merck KGaA, Darmstadt, Germany). 86 % 20 mM KH_2PO_4 (pH = 6.4) and 14 % CH_3CN isocratic flow; 2.5 mL/min; 12 min.

To enable stable conditions for the derivatization throughout the whole analysis sequence, OPA and the thiol compound (3-mercaptopropionic acid (3-MPA) or *N*-acetyl cysteine (NAC)) were kept separately prior to the derivatization step.
OPA solution (200 mM): 18 mg OPA dissolved in 1 mL CH_3CN
3-MPA solution (60 mM): 5.2 µL 3-mercaptopropionic acid (3-MPA) dissolved in 1 mL potassium tetraborate buffer (0.4 M, pH 10.5) + 4.2 mg $KBO_2 \cdot H_2O$
NAC solution (60 mM): 9.8 mg *N*-acetyl cysteine (NAC) dissolved in 1 mL potassium tetraborate buffer (0.4 M, pH 10.5) + 4.2 mg $KBO_2 \cdot H_2O$

For the derivatization of the amino acids, 50 µL of the appropriate thiol solution, 10 µL OPA solution and 10 µL sample (dilution: 50 µL from the reaction + 450 µL 0.4 M potassium borate pH 10.5) were mixed, kept at room temperature for one hour and then analyzed by rp-HPLC

(method_7 or method_8). The conversions were calculated based on peak area comparisons with commercially available *syn*-β-phenylserine as standard reference compound.

Table S1. List of analytical data of amino acid products

		L-TA		D-TA		MS (ESI, pos.) Calc.	MS (ESI, pos.) Found[1)]
Aldehyde	Amino acid	t_{anti} [min] [1)]	t_{syn} [min] [1)]	t_{anti} [min] [1)]	t_{syn} [min] [1)]	$[M+1]^+$	$[M+1]^+$
**1a**	**2b**	1.57<br>7.5[2)]	2.59<br>4.2[2)]	1.57<br>7.33[2)]	2.58<br>3.9[2)]	196.0	195.9
**1a**	**2a**	1.4	1.7	1.4	1.7	182.1	182.1
**1a**	**2c**	1.3	1.8	1.3	1.8	212.1	212.1
**1a**	**2e**	2.2	4.91	2.2	4.9	210.1	210.1
**(*m*)-1b**	**2b**	13.7[2)]	6.5[2)]	13.6[2)]	5.9[2)]		
**1c**	**2b**	5.7[2)]	2.8[2)]	5.3[2)]	2.7[2)]		
**1d**	**2b**	5.47[2)]	4.36[2)]	5.42[2)]	4.36[2)]		
**(*o*)-1e**	**2b**	4.42	5.09	4.41	5.07	230.1	230.0
**(*m*)-1e**	**2b**	3.33	3.73	3.30	3.71	230.1	229.9
**(*p*)-1e**	**2b**	3.55	4.01	-	-	230.1	229.9
**(*o*)-1f**	**2b**	5.1[2)]	3.5[2)]	-	3.33[2)]		
**(*m*)-1f**	**2b**	15.0[2)]	6.5[2)]	-	6.1[2)]		
**(*p*)-1f**	**2b**	15.4[2)]	7.5[2)]	-	7.35[2)]		
**(*o*)-1g**	**2b**	3.76	4.12	-	-	274.0	273.8
**(*m*)-1g**	**2b**	3.76	4.11	-	-	274.0	273.8
**(*p*)-1g**	**2b**	3.80	4.19	-	-	274.0	273.8
**(*o*)-1h**	**2b**	-	-	-	-		
**(*m*)-1h**	**2b**	-	-	-	-		
**(*p*)-1h**	**2b**	-	-	-	-		
**(*m*)-1i**	**2b**	3.21	3.50	3.23	3.54	238.1	238.1
**(*p*)-1i**	**2b**	2.91	3.26	2.92	3.27	238.1	238.1
**(*m*)-1j**	**2b**	4.42	4.78	4.42	4.78	254.1	254.1
**(*p*)-1j**	**2b**	4.21	4.80	-	-	254.1	254.1
**1k**	**2b**	7.12[2)]	5.66[2)]	7.08[2)]	5.61[2)]		
**1l**	**2b**	9.75[2)]	6.24[2)]	9.88[2)]	6.26[2)]		
**1m**	**2b**	6.07[2)]	4.2[2)]	5.67[2)]	3.65[2)]		
**1n**	**2b**	9.5[2)]	6.0[2)]	8.8[2)]	5.1[2)]		
**1o**	**2b**	3.37	3.5	3.37	3.5	240.1	240.1
**1p**	**2b**	8.74[2)]	4.1[2)]	10.8[2)]	4.78[2)]		
**1q**	**2b**	7.98[2)]	4.0[2)]	6.92[2)]	3.87[2)]		
**1r**	**2b**	4.95	6.32	-	-	247.1	247.1
**1s**	**2b**	4.73[2)]	2.1[2)]	4.71[2)]	2.0[2)]		
**1t**	**2b**	9.4[2)]	6.3[2)]	9.28[2)]	6.2[2)]		
**1u**	**2b**	14.7[2)]	7.24[2)]	8.9[2)]	5.9[2)]		
**1v**	**2b**	4.96[2)]	2.83[2)]	3.67[2)]	2.58[2)]		
**1w**	**2b**	7.54[2)]	4.77[2)]	7.25[2)]	4.25[2)]		
**1x**	**2b**	4.52	4.58	4.51	4.57	297.1	296.9

1) Determined by LC-MS using method_2
2) Determined by LC after pre-column derivatization with OPA/NAC using method_8

Gas chromatography

GC-MS measurements were performed on an Agilent Technologies 7890A (G3440A) GC system equipped with an Agilent Technologies J&W GC-column HP-5MS ((5%-phenyl) methylpolysiloxane; length: 30 m; inner-diameter: 0.250 mm; film: 0.25 μm) at a constant helium flow rate with He 5.0 as carrier gas. The GC was coupled to a 5975C inert mass sensitive detector with triple-axis detector (MSD, EI, 70 eV; transfer line: 300°C; MS source: 240°C; MS quad: 180°C).

method_A: 50°C 1 min, ramp: 40°C·min^{-1} linear to 300°C, 300°C 5 min, solvent delay: 4.0 min.

1.3. Preparation of threonine aldolases

The vectors pEamTA-TAaj (for L-TA from *Aeromonas jandaei*)[2] and pEamTA-TAps (for the D-TA from *Pseudomonas* sp.)[2b] were re-transformed into *E.coli* BL21AI cells. This constructs contain the *tac* promoter and the LacI repressor, which control the protein expression level. The cells were cultivated on 2×TY media supplemented with 100 mg/mL ampicillin. 100 mL overnight cultures in 300 mL flasks were inoculated with single colonies and grown at 37 °C with shaking. The 330 mL main cultures in baffled 1000 mL flasks were inoculated with 7 ml of the pre-culture and grown at 37 °C for approximately 3 h to an OD600 of 1.5. Temperature was then lowered to 25 °C (L-TA) or 16 °C (D-TA) and induced with 0.1 mM of β-D-1-thiogalactopyranoside (IPTG). Cultivation was continued overnight and cells harvested by centrifugation for 15 min at 4500 × g. After re-suspension of the pellets in sodium phosphate buffer (0.1 M, pH 7.0), the cells were disrupted by ultrasonic treatment for 7 min. The crude lysate was cleared by centrifugation at 20000 × g for 1 h at 4 °C and the supernatant (cell-free extract) was used for the experiments without further purification.

1.4. Screening of the reaction conditions

1.4.1. Determination of the pH optimum in threonine aldolase-catalyzed reactions

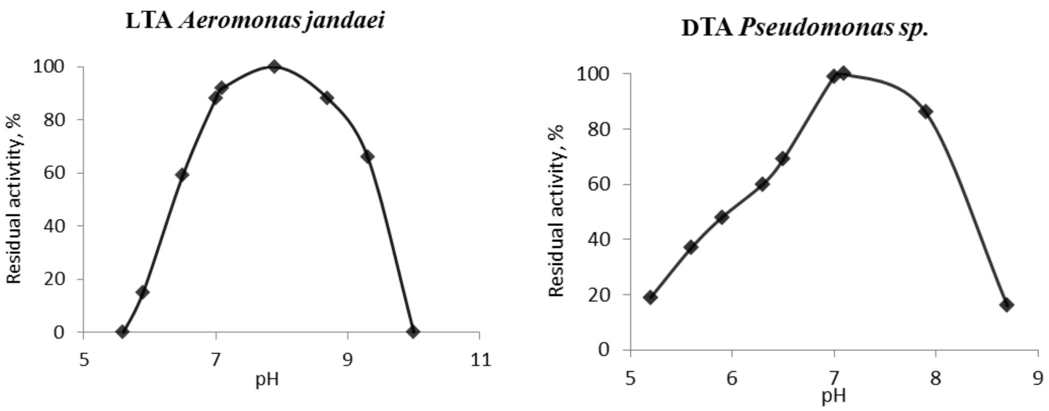

Figure S1. Effect of the pH on the conversion in aldol condensations of benzaldehyde and D-alanine in the presence of L-TA or D-TA after 24 h. Conditions: 50 mM benzaldehyde, 500 mM D-alanine, 1.0 mg TA, 50 µM PLP, 50 mM citrate buffer (pH 5.0-6.0); 50 mM sodium phosphate buffer (pH 6.0 – 8.0); 50 mM sodium bicarbonate buffer (pH 9.0-10.0)

1.4.2. Investigation of the influence of co-solvents on the threonine aldolase-catalyzed reactions

Table S2. Influence of cosolvent addition on the conversion in aldol condensations catalyzed by L-TA and D-TA.

Cosolvent	L-TA rel. conv. [%]	D-TA rel. conv. [%]
No	100	100
DMSO	124	110
Methanol	110	100
Ethanol	100	80
Dioxane	110	110
DMF	95	80
Acetonitrile	120	100
MTBE	25	95
Cyclohexane	50	60
THF	100	90
Dichlormethane	30	15
Acetone	0	15
2-Propanol	95	110
Ethyl acetate	50	80

Conditions: 50 mM benzaldehyde, 500 mM D-alanine, 1.0 mg TA, 50 µM PLP, 50 mM sodium phosphate buffer (pH 8.0); 15 vol% cosolvent; 24 h at 30°C (L-TA), 20°C (D-TA).

1.4.3. Influence of divalent manganese ions on the D-TA-catalyzed reactions

Figure S2. Influence of Mn^{2+} ions on the conversion and the *d.e.* in aldol condensations catalyzed by D-TA.

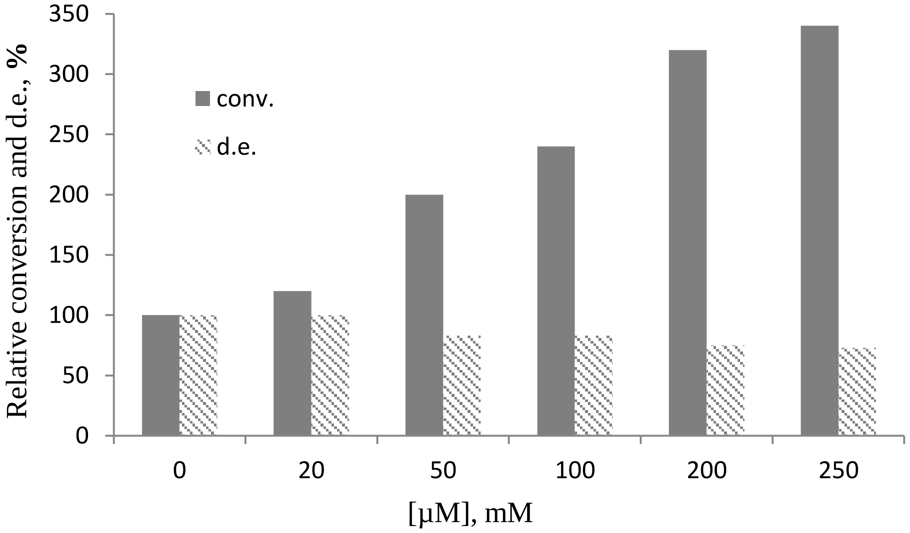

Reaction conditions: 50 mM benzaldehyde, 500 mM D-alanine, 1 mg D-TA, 50 mM sodium phosphate buffer (pH 8.0); 50 µM PLP; 0-250 µM $MnCl_2$; 24 h at 20°C.

1.5. Experimental procedure for threonine aldolase catalyzed reactions

1.5.1. General procedure for the substrate screening in the aldol reactions catalyzed by L-TA and D-TA

In 1.5-mL polypropylene tubes (Eppendorf, Hamburg, Germany), L-TA from *Aeromonas jandaei* or D-TA from *Pseudomonas sp.* (50 µL; 15.5 units) was added to a reaction mixture containing pyridoxal-5´-phosphate (100 µM), D-alanine (44.5 mg, 0.5 M), 50 mM sodium phosphate buffer pH 8.0, DMSO (10 vol%,) and an aldehyde (50 mM) in total volume 1 mL. The reaction mixture was shaken in an Eppendorf shaker at 25 °C and 750 rpm. A sample (50 µL) was taken after 24 h, diluted with 0.4 M potassium borate buffer (450 µL) and then analyzed by HPLC.

1.5.2. Biocatalytic syntheses of serine derivatives at preparative scale

(2*S*)-2-Amino-3-phenyl-3-hydroxy-2-methylpropanoic acid ((2*S*)-**3a**, Table 3, entry 1):

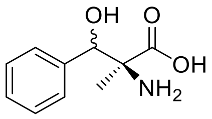

In a 250 mL round bottom flask, 10.69 g (120.0 mmol, 6.0 eq) DL-alanine **2a** was dissolved in 70 mL 100 mM potassium phosphate (KPi) buffer pH 8.0. Under gentle stirring, 4.3 mg (16.0 µmol) PLP was added. To the yellow solution was then added 800 µL benzaldehyde **1a** and 5.0 mL L-TA (1550 U) and the reaction mixture stirred at r. t. After 3 h, further 600 µL benzaldehyde **1a** was added. Another 3 h later, 631 µL benzaldehyde **1a** (to a total amount of 2.031 mL, 20.0 mmol) and 2.5 mL L-TA (to a total amount of 10.0 mL) were added and gentle stirring continued at r.t. After 20 h, 2.5 mL L-TA and 5.1 mL (6 vol%) isopropanol were added. The reaction was stopped after 48 h by the addition of 400 mL methanol at r. t. The colorless precipitate which mostly contains DL-alanine **2a** was removed by filtration and washed with methanol (3 × 15 mL). Subsequently, the filtrate was concentrated under reduced pressure. The 4.344 g of crude material containing the desired product (2*S*)-**3a** were purified via flash chromatography (45 g silica gel, 10 × 3.0 cm, CH_2Cl_2/CH_3OH = 75:25 + 5 vol % NH_3). All fractions containing the product were pooled and the solvents were removed under reduced pressure.

Yield: 507 mg (2.60 mmol, 13 %), colorless solid.

$C_{10}H_{13}NO_3$ [195.22 g/mol]

$[\alpha]_D^{25}$ = -23.6 (c = 0.2 in 1 M HCl)

mp = 188 °C (dec.)

R_f = 0.60 (CH_2Cl_2/CH_3OH /NH_3 = 10/10/1, UV und ninhydrin staining)

^{1}H-NMR (300.36 MHz, D_2O): δ = 7.50 – 7.32 (m, 5H, H_{Ar}), 5.13 (s, 0.72H, -C$\underline{H}$OH, *syn*), 5.06 (s, 0.37H, -C$\underline{H}$OH, *anti*), 1.62 (s, 0.94H, -C$C\underline{H}_3NH_2$, *anti*), 1.28 (s, 2.00H, -C$C\underline{H}_3NH_2$, *syn*). *Syn/anti* = 2.15.

^{13}C-NMR (75.53 MHz, D_2O): δ = 175.4 (C_q, -$\underline{C}$OOH, *anti*), 174.3 (C_q, -$\underline{C}$OOH, *syn*), 137.6 (C_q, *anti*), 137.3 (C_q, *syn*), 128.9 (C_{Ar}), 128.8 (C_{Ar}), 128.7 (C_{Ar}), 127.3 (C_{Ar}, *syn*), 126.7 (C_{Ar}, *anti*), 75.0 (-$\underline{C}$HOH, *syn*), 74.7 (-$\underline{C}$HOH, *anti*), 65.9 (C_q, -$\underline{C}CH_3NH_2$, *anti*), 65.0 (C_q, -$\underline{C}CH_3NH_2$, *syn*), 20.0 (-C$\underline{C}H_3NH_2$, *anti*), 18.8 (-C$\underline{C}H_3NH_2$, *syn*).

(2*R*)-2-Amino-3-phenyl-3-hydroxy-2-methylpropanoic acid ((2*R*)-**3a**, Table 3, entry 2):

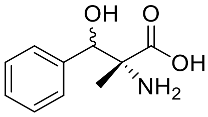

In a 250 mL round bottom flask, 10.69 g (120.0 mmol, 6.0 eq) DL-alanine **2a** was dissolved in 70 mL 100 mM KPi buffer pH 8.0. Under gentle stirring, 4.3 mg (16.0 µmol) PLP was added. To the yellow solution was then added 800 µL benzaldehyde **1a** and 5.0 mL D-TA (600 U) and the reaction mixture stirred at r. t. After 16 h, 1231 µL benzaldehyde **1a** (to a total amount of 2.031 mL, 20.0 mmol) and 5.0 mL D-TA (to a total amount of 10.0 mL) were added and gentle stirring continued at r.t. The reaction was stopped after 72 h by the addition of 400 mL methanol. The colorless precipitate which mostly contains DL-alanine **2a** was removed by filtration and washed with methanol (3 × 15 mL). Subsequently, the filtrate was concentrated under reduced pressure. The 5.738 g of crude material containing the desired product (2*R*)-3a were purified via flash chromatography (30 g silica gel, 10 × 2.5 cm, CH_2Cl_2/CH_3OH = 75:25 + 5 vol% NH_3). All fractions containing the product were pooled and the solvents were removed under reduced pressure.

Yield: 481 mg (2.46 mmol, 12 %), colorless solid.

$C_{10}H_{13}NO_3$ [195.22 g/mol]

$[\alpha]_D^{25}$ = 32.1 (c = 0.2 in 1 M HCl)

mp = 216 °C (dec.)

R_f = 0.60 (CH_2Cl_2/CH_3OH /NH_3 = 10/10/1, UV und ninhydrin staining)

^{1}H-NMR (300.36 MHz, D_2O): δ = 7.50 – 7.32 (m, 5H, H_{Ar}), 5.10 (s, 0.93H, -C$\underline{H}$OH, *syn*), 5.03 (s, 0.17H, -C$\underline{H}$OH, *anti*), 1.59 (s, 0.34H, -C$C\underline{H}_3NH_2$, *anti*), 1.26 (s, 2.64H, -C$C\underline{H}_3NH_2$, *syn*). *Syn/anti* = 7.8.

^{13}C-NMR (75.53 MHz, D_2O): δ = 175.4 (C_q, -$\underline{C}$OOH, *anti*), 174.3 (C_q, -$\underline{C}$OOH, *syn*), 137.6 (C_q, *anti*), 137.3 (C_q, *syn*), 128.9 (C_{Ar}), 128.8 (C_{Ar}), 128.7 (C_{Ar}), 127.3 (C_{Ar}, *syn*), 126.7 (C_{Ar}, *anti*), 75.0 (-$\underline{C}$HOH, *syn*), 74.7 (-$\underline{C}$HOH, *anti*), 65.9 (C_q, -$\underline{C}CH_3NH_2$, *anti*), 65.0 (C_q, -$\underline{C}CH_3NH_2$, *syn*), 20.0 (-C$\underline{C}H_3NH_2$, *anti*), 18.8 (-C$\underline{C}H_3NH_2$, *syn*).

(2*S*)-2-Amino-3-hydroxy-2-methyl-3-(2-nitrophenyl)propanoic acid ((2*S*)-**(*o*)-3b**, Table 3, entry 3):

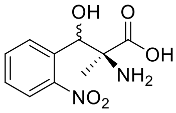

In a 100 mL round bottom flask, 6.41 g (72.0 mmol, 6.0 eq) DL-alanine **2b** was dissolved in 40 mL 100 mM KPi buffer pH 8.0. Under stirring, 2.6 mg (10.0 µmol) PLP were added and the yellow solution was stirred at r. t. for 15 min. Then 1.81 g (12.0 mmol, 1.0 eq) 2-nitrobenzaldehyde ***(o)*-1b** was dissolved in 3 mL DMSO to prepare a stock solution. 2.5 mL of the 2-nitrobenzaldehyde/DMSO solution, 4 mL DMSO and 3 mL L-TA were added. The suspension was stirred at r. t. for 18 h. After 18 h, further 0.5 mL 2-nitrobenzaldehyde/DMSO solution, 1 mL DMSO and 2.5 mL L-TA were added. The suspension was stirred at r. t. for 6 d. The conversion was monitored via HPLC-MS. 7 mL conc. HCl was added to the yellow, cloudy suspension to stop the reaction and the solution stirred at r. t. for 30 min. The colorless precipitate which contains no product was removed by filtration and washed with 0.1 M HCl (2 × 25 mL). After evaporation of the solvent under reduced pressure, a solid-phase extraction through a pad of silica gel (diameter: 6 cm, height: 2 cm) was performed. The filter cake was washed first with 100 % CH_2Cl_2 (3 × 100 mL), then with methanol/H_2O = 7:3 (4 × 100 mL fractions). The fractions were collected and the solvent was removed under reduced pressure. The product (2*S*)-***(o)*-3b** was purified via reversed-phase flash chromatography (60 g silica gel C18, 12 × 3 cm, H_2O/CH_3OH = 100:0 + 0.1 % formic acid (fraction 1 – 5), 98:2 + 0.1 % formic acid (fraction 6 – 8), 80:20 + 0.1 % formic acid (fractions 9 – 11), 50 mL fractions). The resulting yellow solid dissolved again in 3 mL CH_2Cl_2/CH_3OH (80:20 + 5 % NH_3) and the solution was filtered through a pad of silica gel (diameter: 2 cm, height: 2.5 cm) and the filter cake was washed with CH_2Cl_2/CH_3OH = 80:20 + 5 % NH_3 (11 × 25 mL fractions). The filtrates of fraction 5 - 10 were collected and the solvent was removed under reduced pressure.
Yield: 410 mg (1.71 mmol, 14 %), yellow solid.

$C_{10}H_{12}N_2O_5$ [240.07 g/mol]

$[\alpha]_D^{25}$ = -7.1 (c = 0.2 in 1 M HCl)

mp = 158 - 166 °C (dec.)

HPLC-MS (method_1): t_{R1} = 2.29 min, t_{R2} = 2.65 min

^{1}H-NMR (300.36 MHz, D_2O): δ = 8.05 (d, $^3J_{HH}$ = 8.2 Hz, 2H, H_{Ar}), 7.91 – 7.64 (m, 6H, H_{Ar}), 6.07 (s, 1H, -C<u>H</u>OH, *syn*), 5.81 (s, 1H, -C<u>H</u>OH, *anti*), 1.53 (s, 1H, -CC<u>H</u>$_3$NH$_2$, *anti*), 1.29 (s, 1H, -CC<u>H</u>$_3$NH$_2$, *syn*).

^{13}C-NMR (75.53 MHz, D_2O): δ = 174.6 (C_q, -<u>C</u>OOH), 148.4 (C_{Ar}), 133.9 (C_{Ar}), 133.3 (C_{Ar}), 133.1 (-<u>C</u>NO$_2$), 132.1 (-<u>C</u>NO$_2$), 129.7 (C_{Ar}), 129.0 (C_{Ar}), 128.9 (C_{Ar}), 125.1 (C_{Ar}), 124.9 (C_{Ar}), 69.3 (-<u>C</u>HOH), 68.6 (-<u>C</u>HOH), 65.4 (-<u>C</u>CH$_3$NH$_2$), 65.3 (-<u>C</u>CH$_3$NH$_2$), 20.1 (-C<u>C</u>H$_3$NH$_2$), 17.6 (-C<u>C</u>H$_3$NH$_2$).

HRMS (MALDI-TOF): Calcd. for $C_{10}H_{12}N_2O_5H$ $[M+H]^+$: 241.0824; found: 241.0845.

(2*S*)-2-Amino-3-hydroxy-2-methyl-3-(3-nitrophenyl)propanoic acid ((2*S*)-**(*m*)-3b**, Table 3, entry 4):

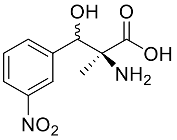

In a 100 mL round bottom flask, 6.25 mL 1.6 M DL-alanine solution in H_2O, 100 µL 10 mM PLP, 1 mL 1 M KPi buffer pH 8.0 and 1.15 mL dist. H_2O was stirred at r. t. for 10 min. Then 75.6 mg (500.0 µmol, 1.0 eq) 3-nitrobenzaldehyde **(*m*)-1b** was dissolved in 1 mL DMSO to prepare a stock solution. The 3-nitrobenzaldehyde/DMSO solution and 0.5 mL L-TA were added. The suspension was shaken at 120 rpm at 30 °C for 24 h. The conversion was monitored via HPLC-MS. The yellow, cloudy suspension was finally heated to 80 °C for 90 min to inactivate the enzyme. 40 mL methanol was added to the suspension and the solution was stirred at r. t. for 2 h. The colorless precipitate which contains no product was removed through a pad of silica gel (diameter: 6 cm, height: 1 cm) and the filter cake was subsequently washed with CH_3OH/H_2O = 7:1 (3 × 20 mL fractions). The filtrates were collected and the solvent was removed under reduced pressure. DMSO was removed under high vacuum. Then the product (2*S*)-**(*m*)-3b** was purified via flash chromatography (15 g silica gel, 8 × 2 cm, CH_2Cl_2/ CH_3OH = 80:20 + 5 % NH_3 (fraction 1 – 13), 20 mL fractions). Fractions 8 - 11 were collected and the solvent was removed under reduced pressure.

Yield: 23.2 mg (97 µmol, 16 %), yellowish solid.

$C_{10}H_{12}N_2O_5$ [240.07 g/mol]

$[\alpha]_D^{25}$ = -22.1 (c = 0.2 in 1 M HCl)

mp = 162 - 180 °C (dec.)

HPLC-MS (method_3): t_{R1} = 3.12 min, t_{R2} = 3.42 min

^{1}H-NMR (300.36 MHz, D_2O) : δ = 8.27 – 8.17 (m, 4H, H_{Ar}), 7.81 – 7.73 (m, 2H, H_{Ar}), 7.66 – 7.56 (m, 2H, H_{Ar}), 5.31 (s, 1H, -C$\underline{H}$OH, *syn*), 5.17 (s, 1H, -C$\underline{H}$OH, *anti*), 1.60 (s, 1H, -C$\underline{H}_3$NH$_2$, *anti*), 1.38 (s, 1H, -CC$\underline{H}_3$NH$_2$, *syn*). ^{13}C-NMR (75.53 MHz, D_2O): δ = 172.5 (C_q, -$\underline{C}$OOH), 171.4 (C_q, -$\underline{C}$OOH), 148.1 (C_q, -$\underline{C}$NO$_2$), 147.9 (C_q, -$\underline{C}$NO$_2$), 139.1 (C_q, C_{Ar}), 138.3 (C_q, C_{Ar}), 134.1 (C_{Ar}), 133.7 (C_{Ar}), 130.9 (C_{Ar}), 130.1 (C_{Ar}),129.8 (C_{Ar}), 126.3 (C_{Ar}), 124.3 (C_{Ar}), 124.1 (C_{Ar}), 123.1 (C_{Ar}), 122.4 (C_{Ar}), 122.0 (C_{Ar}), 74.0 (-$\underline{C}$HOH), 74.5 (-$\underline{C}$HOH), 64.4 (-$\underline{C}$CH$_3$NH$_2$), 64.0 (-$\underline{C}$CH$_3$NH$_2$), 19.2 (-C$\underline{C}$H$_3$NH$_2$), 17.8 (-C$\underline{C}$H$_3$NH$_2$). HRMS (MALDI-TOF): Calcd. for $C_{10}H_{12}N_2O_5H$ $[M+H]^+$: 241.0824; found: 241.0826.

(2*S*)-2-Amino-3-hydroxy-2-methyl-3-(4-nitrophenyl)propanoic acid ((2*S*)-**(*p*)-3b**, Table 3, entry 5):

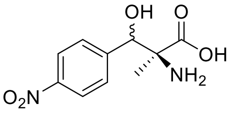

In a 100 mL round bottom flask, 6.41 g (72.0 mmol, 6.0 eq) DL-alanine **2b** was dissolved in 40 mL 100 mM KPi buffer pH 8.0. Under stirring, 2.5 mg (10.0 µmol) PLP were added and the yellow solution was stirred at r. t. for 15 min. Then 1.81 g (12.0 mmol, 1.0 eq) 4-nitrobenzaldehyde **(*p*)-1b** was dissolved in 3 mL DMSO to prepare a stock solution. 2.5 mL of the 4-nitrobenzaldehyde/DMSO solution, 4 mL DMSO and 3 mL L-TA were added. The suspension was stirred at r. t. for 18 h. Then another 0.5 mL 4-nitrobenzaldehyde/DMSO solution, 1 mL DMSO and 2.5 mL L-TA were added. The suspension was stirred at r. t. for 9 d. The conversion was monitored via HPLC-MS. 7 mL conc. HCl was finally added to the yellowish, cloudy suspension to inactivate the enzyme and the solution was stirred at r. t. for 30 min. The colorless precipitate containing no product was removed by filtration and washed with 1 M HCl (2 × 10 mL). After evaporation of the solvent under reduced pressure a filtration through a pad of silica gel (diameter: 6 cm, height: 2 cm) was performed. The filter cake was washed first with CH_2Cl_2/ CH_3OH = 75:25 + 5 % NH_3 (2 × 100 mL) and then with CH_2Cl_2/CH_3OH = 65:35 + 5 % NH_3 (2 × 100 mL). The filtrates were collected and the solvent was removed under reduced pressure. After evaporation of the solvent under reduced pressure, the product (2*S*)-**(*p*)-3b** was purified via flash chromatography (60 g silica gel C18, 11 × 3 cm, H_2O/CH_3OH = 98:2 + 0.1 % formic acid (fraction 1 – 8), 95:5 + 0.1 % formic acid (fractions 9 – 14), 50 mL fractions), followed by normal-phase chromatography (20 g silica gel, 16 × 2 cm, CH_2Cl_2/CH_3OH = 85:15 + 2.5 % NH_3 (fraction 1 – 49), 75:25 + 5 % NH_3 (fraction 50-57), 50 mL fractions) to reach the desired final purity. Fractions 42 - 55 were collected and the solvent was removed under reduced pressure.

Yield: 210 mg (873 µmol, 7 %), yellowish solid.

$C_{10}H_{12}N_2O_5$ [240.07 g/mol]

$[\alpha]_D^{25}$ = -13.3 (c = 0.2 in 1 M HCl)

mp = 167 - 178 °C (dec.)

R_f = 0.20 ($CH_2Cl/CH_3OH/NH_3$ = 75/25/1, UV und Ninhydrin)

HPLC-MS (method_1): t_{R1} = 2.45 min, t_{R2} = 2.90 min

^{1}H-NMR (300.36 MHz, D_2O): δ = 8.28 – 8.20 (m, 4H, H_{Ar}), 7.67 – 7.58 (m, 4H, H_{Ar}), 5.33 (s, 1H, -C$\underline{H}$OH, *syn*), 5.19 (s, 1H, -C$\underline{H}$OH, *anti*), 1.63 (s, 1H, -C$C\underline{H}_3NH_2$, *anti*), 1.41 (s, 1H, -C$C\underline{H}_3NH_2$, *syn*).
^{13}C-NMR (75.53 MHz, D_2O): δ = 172.5 (C_q, -$\underline{C}$OOH), 171.4 (C_q, -$\underline{C}$OOH), 148.1 (C_q, -$\underline{C}NO_2$), 148.0 (C_q, -$\underline{C}NO_2$), 144.5 (C_q, C_{Ar}), 143.7 (C_q, C_{Ar}), 128.6 (C_{Ar}), 128.2 (C_{Ar}), 123.9 (C_{Ar}), 123.7 (C_{Ar}), 74.1 (-$\underline{C}$HOH), 73.7 (-$\underline{C}$HOH), 64.4 (-$\underline{C}CH_3NH_2$), 64.0 (-$\underline{C}CH_3NH_2$), 19.3 (-C$\underline{C}H_3NH_2$), 17.8 (-C$\underline{C}H_3NH_2$).
HRMS (MALDI-TOF): Calcd. for $C_{10}H_{12}N_2O_5H$ $[M+H]^+$: 241.0829; found: 241.0824.

(2*S*)-2-Amino-3-(2-chlorophenyl)-3-hydroxy-2-methylpropanoic acid ((2*S*)-**(*o*)-3e**, Table 3, entry 6):

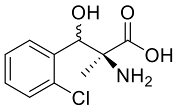

In a 500 mL round bottom flask, 19.39 g (218.0 mmol, 10.0 eq) DL-alanine **2b** was dissolved in 11 mL 100 mM KPi buffer pH 8.0, 157 mL H_2O and 3.8 mL isopropanol. Under stirring, 6 mg PLP were added and the yellow solution was stirred at r. t. for 30 min. Then 5 mL L-TA were added. A solution of 3.1 g (21.8 mmol, 1.0 eq) 2-chlorobenzaldehyde **1e** in 18 mL isopropanol was added under gentle stirring via a syringe pump over 24 h. After addition of another 5 mL L-TA the suspension was stirred at r. t. for altogether 3 d. The conversion was monitored via HPLC-MS. 1 mL formic acid was added and the yellowish, cloudy suspension was heated to 80 °C to inactivate the enzyme. After 30 min the suspension was allowed to cool to r. t. The suspension was filtrated and the filtrate was extracted with ethyl acetate (1 × 100 mL). The aqueous phase was concentrated by rotary evaporation. The product (2*S*)-**(*o*)-3e** was purified via reversed-phase flash chromatography (60 g silica gel C18, 11 × 3 cm, H_2O/CH_3OH = 99:1 + 0.1 % formic acid (fraction 1 – 6), 90:10 + 0.1 % formic acid (fraction 7 – 9), 85:15 + 0.1 % formic acid (fraction 10 – 14), 50 mL fractions). Fractions 8 - 11 were collected and the solvent was removed under reduced pressure.
Yield: 807 mg (3.51 mmol, 16 %), colorless solid.
$C_{10}H_{12}ClNO_3$ [229.05 g/mol]
$[\alpha]_D^{25}$ = -9.1 (c = 0.1 in 1 M HCl)
mp = 195 - 212 °C (dec.)
HPLC-MS (method_3): t_{R1} = 4.34 min, t_{R2} = 5.00 min

^{1}H-NMR (300.36 MHz, D_2O): δ = 7.61 – 7.34 (m, 4H, H_{Ar}), 5.73 (s, 1H, $-C\underline{H}OH$, *syn*), 5.47 (s, 1H, $-C\underline{H}OH$, *anti*), 1.54 (s, 1H, $-CC\underline{H}_3NH_2$, *anti*), 1.35 (s, 1H, $-CC\underline{H}_3NH_2$, *syn*).
^{13}C-NMR (75.53 MHz, D_2O): δ = 175.1 ($C_{q,}$ $-\underline{C}OOH$), 173.4 ($C_{q,}$ $-\underline{C}OOH$), 135.5 (C_q, C_{Ar}), 135.4 (C_q, C_{Ar}), 133.3 (C_q, $-\underline{C}Cl$), 132.7 (C_q, $-\underline{C}Cl$), 130.2 (C_{Ar}), 130.1 (C_{Ar}), 129.8 (C_{Ar}), 129.6 (C_{Ar}), 129.1 (C_{Ar}), 128.4 (C_{Ar}), 127.6 (C_{Ar}), 127.1 (C_{Ar}), 71.3 ($-\underline{C}HOH$), 70.3 ($-\underline{C}HOH$), 65.7 (C_q, $-\underline{C}CH_3NH_2$), 65.1 (C_q, $-\underline{C}CH_3NH_2$), 19.2 ($-C\underline{C}H_3NH_2$), 18.3 ($-C\underline{C}H_3NH_2$).
HRMS (MALDI-TOF): Calcd. for $C_{10}H_{12}ClNO_3H$ $[M+H]^+$: 230.0584; found: 230.0585.

(2*S*)-2-Amino-3-(2-fluorophenyl)-3-hydroxy-2-methylpropanoic acid ((2*S*)-**(*o*)-3f**, Table 3, entry 7):

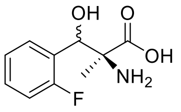

In a 100 mL round bottom flask, 6.41 g (72.0 mmol, 6.0 eq) DL-alanine **2b** was dissolved in 40 mL 100 mM KPi buffer pH 8.0. Under stirring, 2.5 mg (10.0 µmol) PLP and 4 mL isopropanol were added. The yellow solution was stirred at r. t. for 15 min. Then 350 µL 2-fluorobenzaldehyde **1f** and 2.5 mL L-TA were added and the solution was stirred at r. t. for 3 h. After 3 h, further 350 µL 2-fluorobenzaldehyde **1f** was added. After 2 h another 560 µL 2-fluorobenzaldehyde **1f** (to a total amount of 1.26 mL, 12.0 mmol, 1.0 eq), 0.8 mL isopropanol and 1.5 mL L-TA were added and the suspension was stirred at r. t. for 6 d. The conversion was monitored via HPLC-MS. 250 mL methanol was added to the yellowish, cloudy suspension and the solution was stirred at r. t. for 2 h. The colorless precipitate was removed by filtration and washed with methanol (3 × 15 mL). The filtrate was concentrated under reduced pressure and the product (2*S*)-**(*o*)-3b** purified via flash chromatography (40 g silica gel, 13 × 2.5 cm, CH_2Cl_2/CH_3OH = 70:30 + 5 % NH_3 (fraction 1 – 9), 20:10 + 5 % NH_3 (fraction 10 – 12), 50 mL fractions). Fractions 6 - 10 were collected and the solvent was removed under reduced pressure.
Yield: 501 mg (2.35 mmol, 20 %), colorless solid.
$C_{10}H_{12}FNO_3$ [213.08 g/mol]
$[\alpha]_D^{25}$ = -15.3 (c = 0.2 in 1 M HCl)
mp = 180 - 191 °C (dec.)
R_f = 0.50 (CH_2Cl_2/CH_3OH /NH_3 = 10/10/1, UV und Ninhydrin)
HPLC-MS (method_1): t_{R1} = 1.47 min, t_{R2} = 1.71 min.

^{1}H-NMR (300.36 MHz, D_2O): δ = 7.56 – 7.40 (m, 4H, H_{Ar}, H_{Ar}), 7.34 – 7.13 (m, 4H, H_{Ar}, H_{Ar}), 5.54 (s, 1H, -C$\underline{H}$OH, *syn*), 5.34 (s, 1H, -C$\underline{H}$OH, *anti*), 1.60 (s, 3H, -C$\underline{H_3}$NH$_2$, *anti*), 1.42 (s, 3H, -C$\underline{H_3}$NH$_2$, *syn*).

^{13}C-NMR (75.53 MHz, D_2O): δ = 172.8 (C_q, -$\underline{C}$OOH), 171.6 (C_q, -$\underline{C}$OOH), 161.6 (C_q, -$\underline{C}$F), 158.3 (C_q, -CF), 131.3 (C_{Ar}), 131.2 (C_{Ar}), 129.0 (C_{Ar}),128.6 (C_{Ar}), 125.0 (C_{Ar}), 124.6 (C_{Ar}), 123.5 (C_q, C_{Ar}), 123.4 (C_q, C_{Ar}), 115.95 (C_{Ar}), 115.8 (C_{Ar}), 69.9 (-$\underline{C}$HOH), 68.6 (-$\underline{C}$HOH), 64.5 (C_q, -$\underline{C}$CH$_3$NH$_2$), 64.2 (C_q, -$\underline{C}$CH$_3$NH$_2$), 18.5 (-C$\underline{C}$H$_3$NH$_2$), 17.5 (-C$\underline{C}$H$_3$NH$_2$).

HRMS (MALDI-TOF): Calcd. for $C_{10}H_{12}FNO_3H$ $[M+H]^+$: 214.0880; found: 214.0872.

(2*R*)-2-Amino-3-(2-fluorophenyl)-3-hydroxy-2-methylpropanoic acid ((2*R*)-**(*o*)-3f**, Table 3, entry 8):

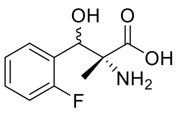

In a 100 mL round bottom flask, 2.67 g (30.0 mmol, 10.0 eq) DL-alanine **2b** was dissolved in 3 mL 100 mM KPi buffer pH 8.0, 21.7 mL dist. H_2O and 3 mL isopropanol. Under stirring, 300 µL PLP were added. The yellow solution was stirred at r. t. for 10 min and then cooled to 15 °C. Then 80 µL 2-fluorobenzaldehyde **1f** and 0.5 mL D-TA were added and the solution was stirred at 15 °C. After 3.5 h another 80 µL 2-fluorobenzaldehyde **1f** were added. The reaction mixture was shaken at 120 rpm at 17.5 – 18.5 °C. After 20 h, further 156 µL 2-fluorobenzaldehyde **1f** (to a total amount of 316 µL, 3.0 mmol, 1.0 eq) and 0.5 mL D-TA were added and the suspension was stirred at 17.5 – 18.5 °C for 3 d whereby the conversion was monitored via HPLC-MS. After adding 0.5 mL formic acid, the reaction mixture was heated to 80 °C for 2 h to inactivate the enzyme. The resulting precipitate was removed by filtration and washed with H_2O + 0.01 % formic acid (3 × 15 mL). The filtrate was concentrated under reduced pressure and the crude product (2*R*)-**(*o*)-3b** was purified via reversed-phase flash chromatography (60 g silica gel C18, 10.5 × 3 cm, H_2O/CH_3OH = 98:2 + 0.01 % formic acid (fraction 1 – 3), 90:10 + 0.01 % formic acid (fraction 4 – 10), 100 ml fractions (F1 -2), 50 mL fractions (F3 – 10)). Fractions 4 - 7 were collected and the solvent was removed under reduced pressure.

Yield: 175 mg (821 µmol, 27 %), colorless solid.

$C_{10}H_{12}FNO_3$ [213.08 g/mol]

$[\alpha]_D^{25}$ = 28.8 (c = 0.2 in 1 M HCl)

mp = 220 - 237 °C (dec.)

HPLC-MS (method_2): t_{R1} = 3.70 min.

^{1}H-NMR (300.36 MHz, D_2O): δ = 7.57 – 7.44 (m, 4H, H_{Ar}, H_{Ar}), 7.34 – 7.29 (m, 2H, H_{Ar}), 7.24 – 7.18 (m, 2H, H_{Ar}), 5.55 (s, 1H, -C<u>H</u>OH, *syn*), 1.42 (s, 3H, -CC<u>H</u>$_3$NH$_2$, *syn*).

^{13}C-NMR (75.53 MHz, D_2O): δ = 173.0 (C_q, -<u>C</u>OOH), 161.6 (C_q, -<u>C</u>F), 158.3 (C_q, -<u>C</u>F), 131.3 (C_{Ar}), 131.2 (C_{Ar}), 128.6 (C_{Ar}), 124.9 (C_{Ar}), 123.6 (C_q, C_{Ar}), 123.5 (C_q, C_{Ar}), 115.9 (C_{Ar}), 115.7 (C_{Ar}), 68.7 (-<u>C</u>HOH), 64.6 (C_q, -<u>C</u>CH$_3$NH$_2$), 17.5 (-C<u>C</u>H$_3$NH$_2$), 17.1 (-C<u>C</u>H$_3$NH$_2$).

HRMS (MALDI-TOF): Calcd. for $C_{10}H_{12}FNO_3H$ $[M+H]^+$: 214.0880; found: 214.0878.

<u>(2*S*)-3-(3-Acetylphenyl)-2-amino-3-hydroxy-2-methylpropanoic acid ((2*S*)-**(*m*)-3i**, Table 3, entry 9):</u>

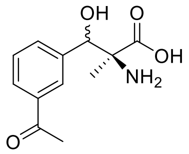

In a 100 mL round bottom flask, 2.67 g (30.0 mmol, 10.0 eq) DL-alanine **2b** was dissolved in 3 mL 1 M KPi buffer pH 8.0, 21.7 mL H_2O and 2 mL isopropanol. Under stirring, 300 µL 10 mM PLP were added and the yellow solution was stirred at r. t. for 10 min. Then a solution of 445 mg (3.0 mmol, 1.0 eq) 3-acetylbenzaldehyde **(*m*)-1i** in 1 mL isopropanol and 1 mL L-TA were added. The suspension was stirred at r. t. for 18 h. After addition of another 1 mL L-TA, the suspension was stirred at r. t. for 3 d. The conversion was monitored via HPLC-MS. 2.5 mL formic acid was added to the yellowish, cloudy suspension to stop the reaction and the solution was stirred at r. t. for 10 min. After evaporation of the solvent under reduced pressure, the product (2*S*)-**(*m*)-3i** was purified via reversed-phase flash chromatography (60 g silica gel C18, 10.5 × 3 cm, H_2O/CH_3OH = 98:2 + 0.1 % formic acid (fraction 1 – 5), 90:10 + 0.1 % formic acid (fraction 6 – 10), 50 mL fractions). Then a short-path normal-phase chromatography was performed to remove residual DL-alanine, in which the yellow solid was dissolved in 2 mL CH_2Cl_2/CH_3OH (80:20 + 5 % NH_3) and filtered through a pad of silica gel (diameter: 2 cm, height: 2 cm) and the filter cake washed with CH_2Cl_2/CH_3OH = 80:20 + 5 % NH_3 (11 × 25 mL fractions). The fractions 6 - 10 were collected and the solvent was removed under reduced pressure.

Yield: 152 mg (640 µmol, 21 %), yellowish solid.

$C_{12}H_{15}NO_4$ [237.10 g/mol]

$[\alpha]_D^{25}$ = -13.9 (c = 0.2 in 1 M HCl)

mp = 173 - 185 °C (dec.)

HPLC-MS (method_3): t_{R1} = 3.37 min, t_{R2} = 3.65 min

^{1}H-NMR (300.36 MHz, D_2O): δ = 8.02 – 7.97 (m, 4H, H_{Ar}), 7.72 – 7.53 (m, 4H, H_{Ar}), 5.22 (s, 1H, -C$\underline{H}$OH, *syn*), 5.13 (s, 1H, -C$\underline{H}$OH, *anti*), 2.68 (d, $^{2}J_{HH}$ = 5.5 Hz, 6H, -CO$\underline{C}H_3$), 1.63 (s, 3H, -C$\underline{H}_3NH_2$, *anti*), 1.30 (s, 3H, -C$\underline{H}_3NH_2$, *syn*).
^{13}C-NMR (75.53 MHz, D_2O): δ = 203.7 (C_q, -$\underline{C}OCH_3$), 203.6 (C_q, -$\underline{C}OCH_3$), 175.2 (C_q, -$\underline{C}$OOH), 174.1 (C_q, -$\underline{C}$OOH), 138.5 (C_q, .-$\underline{C}COCH_3$), 138.2 (C_q, -$\underline{C}COCH_3$), 136.7 (C_q, C_{Ar}) 136.6 (C_q, C_{Ar}), 132.7 (C_{Ar}), 132.2 (C_{Ar}), 129.1 (C_{Ar}), 129.0 (C_{Ar}), 128.8 (C_{Ar}), 128.8 (C_{Ar}), 127.1 (C_{Ar}), 126.7 (C_{Ar}), 74.6 (-$\underline{C}$HOH), 74.4 (-$\underline{C}$HOH), 65.8 (C_q, -$\underline{C}CH_3NH_2$), 65.0 (C_q, -$\underline{C}CH_3NH_2$), 26.4 (-CO$\underline{C}H_3$), 20.1 (-C$\underline{C}H_3NH_2$), 18.4 (-C$\underline{C}H_3NH_2$).
HRMS (MALDI-TOF): Calcd. for $C_{12}H_{15}NO_4H$ $[M+H]^+$: 238.1079; found: 238.1078.

(2*S*)-3-(4-Acetylphenyl)-2-amino-3-hydroxy-2-methylpropanoic acid ((2*S*)-**(*p*)-3i**, Table 3, entry 10):

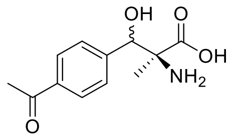

In a 100 mL round bottom flask, 2.67 g (30.0 mmol, 10.0 eq) DL-alanine **2b** was dissolved in 3 mL 100 mM KPi buffer pH 8.0, 21.7 mL dist. H_2O and 2 mL isopropanol. Under stirring, 300 µL 10 mM PLP were added. The yellow solution was stirred at r. t. for 10 min. Then a solution of 445 mg (3.0 mmol, 1.0 eq) 4-acetylbenzaldehyde **(*p*)-1i** in 1 mL isopropanol and 1 mL L-TA were added. The suspension was stirred at r. t. for 18 h. After addition of another 1 mL L-TA, the suspension was further stirred at r. t. for 4 d. The conversion was monitored via HPLC-MS. 2.5 mL formic acid was added to the yellowish, cloudy suspension to stop the reaction and the solution was stirred at r. t. for 20 min. After evaporation of the solvent under reduced pressure, the product (2*S*)-**(*p*)-3i** was purified via reversed-phase flash chromatography (60 g silica gel C18, 10.5 × 3 cm, H_2O/CH_3OH = 98:2 + 0.1 % formic acid (fraction 1 – 5), 90:10 + 0.1 % formic acid (fraction 6 – 10), 50 mL fractions). Fractions 5 - 9 were collected and the solvent was removed under reduced pressure.
Yield: 157 mg (662 µmol, 22 %), yellowish solid.
$C_{12}H_{15}NO_4$ [237.10 g/mol]
$[\alpha]_D^{25}$ = -11.6 (c = 0.2 in 1 M HCl)
mp = 172 - 181 °C (dec.)
HPLC-MS (method_2): t_{R1} = 3.01 min, t_{R2} = 3.36 min.

^{1}H-NMR (300.36 MHz, D_2O): δ = 8.05 – 7.98 (m, 4H, H_{Ar}), 7.59 – 7.51 (m, 8.2 Hz, 4H, H_{Ar}), 5.22 (s, 1H, -C<u>H</u>OH, *syn*), 5.12 (s, 1H, -C<u>H</u>OH, *anti*), 2.7 (s, 3H, -COC<u>H</u>$_3$, *syn*), 2.6 (s, 3H, -COC<u>H</u>$_3$, *anti*), 1.63 (s, 3H, -CC<u>H</u>$_3$NH$_2$, *anti*), 1.31 (s, 3H, -CC<u>H</u>$_3$NH$_2$, *syn*).
^{13}C-NMR (75.53 MHz, D_2O): δ = 203.7 (C_q, -CO<u>C</u>H$_3$), 203.6 (C_q, -CO<u>C</u>H$_3$), 174.8 (C_q, -<u>C</u>OOH), 173.7 (C_q, -<u>C</u>OOH), 143.6 (C_q, C_{Ar}), 143.3 (C_q, C_{Ar}), 136.8 (C_q, -<u>C</u>COCH$_3$), 136.7 (C_q, -<u>C</u>COCH$_3$), 128.8 (C_{Ar}), 128.7 (C_{Ar}), 127.7 (C_{Ar}), 127.2 (C_{Ar}), 74.5 (-<u>C</u>HOH), 74.4 (-<u>C</u>HOH), 65.8 (C_q, -<u>C</u>CH$_3$NH$_2$), 64.9 (C_q, -<u>C</u>CH$_3$NH$_2$), 26.4 (-CO<u>C</u>H$_3$), 20.0 (-C<u>C</u>H$_3$NH$_2$), 18.3 (-C<u>C</u>H$_3$NH$_2$).
HRMS (MALDI-TOF): Calcd. for $C_{12}H_{15}NO_4H$ $[M+H]^+$: 238.1079; found: 238.1081.

<u>(2*S*)-2-Amino-3-hydroxy-3-[3-(methoxycarbonyl)phenyl]-2-methyl-propanoic acid ((2*S*)-**(*m*)-3j**, Table 3, entry 11):</u>

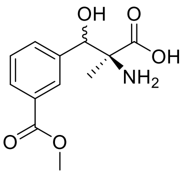

In a 100 mL round bottom flask, 2.67 g (30.0 mmol, 10.0 eq) DL-alanine **2b** was dissolved in 3 mL 100 mM KPi buffer pH 8.0, 21.7 mL H_2O and 2 mL isopropanol. Under stirring, 300 µL 10 mM PLP were added and the yellow solution was stirred at r. t. for 10 min. Then a solution of 492 mg (3.0 mmol, 1.0 eq) methyl 3-formylbenzoate **(*m*)-1j** in 1 mL isopropanol and 1 mL LTA were added. The suspension was stirred at r. t. for 18 h. After addition of another 1 mL LTA, the suspension was stirred at r. t. for 4 d. The conversion was monitored via HPLC-MS. 2.5 mL formic acid was added to the yellowish, cloudy suspension to stop the reaction and the solution was stirred at r. t. for 15 min. After evaporation of the solvent under reduced pressure, the product (2*S*)-**(*m*)-3j** was purified via reversed-phase flash chromatography (60 g silica gel C18, 10.5 × 3 cm, H_2O/CH_3OH = 98:2 + 0.1 % formic acid (fraction 1 – 4), 90:10 + 0.1 % formic acid (fraction 5 – 14), 50 mL fractions). Fractions 6 - 13 were collected and the solvent was removed under reduced pressure.
Yield: 210 mg (829 µmol, 28 %), colorless solid.
$C_{12}H_{15}NO_5$ [253.10 g/mol]
$[\alpha]_D^{25}$ = -16.3 (c = 0.2 in 1 M HCl)
mp = 175 - 184 °C (dec.)
HPLC-MS (method_2): t_{R1} = 4.48 min, t_{R2} = 4.83 min.

^{1}H-NMR (300.36 MHz, D_2O): δ = 8.04 – 7.94 (m, 4H, H_{Ar}), 7.75 – 7.46 (m, 4H, H_{Ar}), 5.27 (s, 1H, -CHOH, *syn*), 5.14 (s, 1H, -CHOH, *anti*), 3.89 (s, 6H, -$COOCH_3$), 1.62 (s, 3H, -CCH_3NH_2, *anti*), 1.39 (s, 3H, -CCH_3NH_2, *syn*).
^{13}C-NMR (75.53 MHz, D_2O): δ = 172.8 (C_q, -COOH), 171.6 (C_q, -COOH), 169.0 (C_q, -$COOCH_3$), 165.6 (C_q, -$COOCH_3$), 137.6 (C_q, C_{Ar}), 136.8 (C_q, C_{Ar}), 132.4 (C_q, C_{Ar}), 132.0 (C_q, C_{Ar}), 130.2 (C_{Ar}), 130.1 (C_{Ar}), 130.0 (C_{Ar}), 129.8 (C_{Ar}), 129.2 (C_{Ar}), 129.0 (C_{Ar}), 128.3 (C_{Ar}), 127.8 (C_{Ar}), 74.4 (-CHOH), 74.0 (-CHOH), 64.4 (C_q, -CCH_3NH_2), 64.0 (C_q, -CCH_3NH_2), 52.8 (-$COOCH_3$), 19.2 (-CCH_3NH_2), 17.9 (-CCH_3NH_2).
HRMS (MALDI-TOF): Calcd. for $C_{12}H_{15}NO_5H$ $[M+H]^+$: 254.1028; found: 245.1022.

(2*S*)-2-Amino-3-hydroxy-2-methyl-3-(pyridine-3-yl)propanoic acid ((2*S*)-**3k**, Table 3, entry 12):

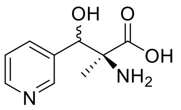

In a 100 mL round bottom flask, 2.67 g (30.0 mmol, 10.0 eq) DL-alanine **2b** was dissolved in 3 mL 100 mM KPi buffer pH 8.0 and 22.7 mL dist. H_2O. Under stirring, 300 µL 10 mM PLP were added and the yellow solution was stirred at r. t. for 10 min. Then 321 mg (3.0 mmol, 1.0 eq) nicotinaldehyde **1k** were dissolved in 2 mL DMSO to obtain a stock solution and 0.5 mL L-TA was added to the reaction vessel. The nicotinaldehyde/DMSO solution was added under stirring in 5 portions over a time span of 8 h. The reaction mixture was shaken at 120 rpm at 30 °C. After addition of another 0.5 mL L-TA, the suspension was shaken at 30 °C for 3 d. The conversion was monitored via HPLC-MS. The yellow, cloudy suspension was finally heated to 80 °C to inactivate the enzyme. After 60 min, the suspension was allowed to cool to r. t. The suspension was concentrated by rotary evaporation. Then the yellow solid was dissolved in 2 mL CH_2Cl_2/CH_3OH (10:9 + 1 vol% NH_3), filtered through a pad of silica gel (diameter: 2 cm, height: 2.5 cm) and the filter cake was washed with CH_2Cl_2/CH_3OH = 10:9 + 1 vol% NH_3 (9 × 50 mL fractions). The filtrates were collected and the solvent was removed under reduced pressure. From the purification via normal-phase flash chromatography (80 g silica gel, 30 × 1.5 cm, CH_2Cl_2/CH_3OH = 80:20 + 5 % NH_3 (fraction 1 – 17), 70:30 + 5 % NH_3 (fraction 18), 60:40 + 5 % NH_3 (fraction 19), 50:50 + 5 % NH_3 (fraction 20), 100:0 + 5 % NH_3 (fraction 21), 50 mL fractions (fraction 1 – 18), 250 mL fractions (fractions 19 – 21)) the fractions 2 to 21 were collected and the solvent was removed under reduced pressure. Co-eluting DMSO was removed under high vacuum prior to the second chromatography step. Finally, the product (2*S*)-**3k** was purified via normal-phase flash chromatography (40 g silica

gel, 30 × 1.5 cm, CH_2Cl_2/CH_3OH = 70:30 + 5 % NH_3 (fraction 1 – 18), 50 mL fractions). Fractions 7 - 15 were collected and the solvent was removed under reduced pressure.
Yield: 134 mg (683 µmol, 23 %), yellow-brownish solid.
$C_9H_{12}N_2O_3$ [196.08 g/mol]
$[\alpha]_D^{25}$ = -17.7 (c = 0.2 in 1 M HCl)
mp = 112 - 168 °C (dec.)
HPLC-MS (method_4): t_R = 0.83 min.
^{1}H-NMR (300.36 MHz, D2O): δ = 8.56 – 8.50 (m, 4H, H_{Ar}), 7.93 – 7.86 (m, 2H, H_{Ar}), 7.54 - 7.46 (m, 1H, H_{Ar}), 5.20 (s, 1H, -C<u>H</u>OH, syn), 5.10 (s, 1H, -C<u>H</u>OH, anti), 1.61 (s, 3H, -CC<u>H</u>$_3$NH$_2$, anti), 1.31 (s, 3H, -CC<u>H</u>$_3$NH$_2$, syn).
^{13}C-NMR (75.53 MHz, D2O): δ = 174.7 (C_q, -<u>C</u>OOH), 173.7 (C_q, -<u>C</u>OOH), 148.9 (C_{Ar}), 148.6 (C_{Ar}), 147.4 (C_{Ar}), 147.0 (C_{Ar}), 136.5 (C_{Ar}), 136.4 (C_{Ar}), 134.4 (C_q, C_{Ar}), 134.1 (C_q, C_{Ar}), 124.2 (C_{Ar}), 124.1 (C_{Ar}), 73.0 (-<u>C</u>HOH), 72.9 (-<u>C</u>HOH), 65.7 (C_q, -<u>C</u>CH$_3$NH$_2$), 65.0 (C_q, -<u>C</u>CH$_3$NH$_2$), 20.0 (-C<u>C</u>H$_3$NH$_2$), 17.9 (-C<u>C</u>H$_3$NH$_2$).
HRMS (MALDI-TOF): Calcd. for C12H15NO5H [M+H]+: 197.0926; found: 197.0927.

<u>(2*S*)-2-Amino-3 hydroxy-2-methyl-3-(quinolin-2-yl)propanoic acid ((2*S*)-**3r**, Table 3, entry 13):</u>

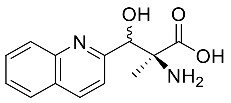

In a 100 mL round bottom flask, 2.67 g (30.0 mmol, 10.0 eq) DL-alanine **2b** was dissolved in 3 mL 100 mM KPi buffer pH 8.0 and 21.7 mL dist. H_2O. Under stirring, 300 µL 10 mM PLP were added and the yellow solution was stirred at r. t. for 10 min. Then 1 mL L-TA was added. A stock solution of 472 mg (3.0 mmol, 1.0 eq) quinolone-2-carbaldehyde **1r** in 2 mL ethanol was added under stirring in 5 portions over 8 h. After 20 h another 1 mL L-TA was added. The suspension was stirred at r. t. for 7 d. The conversion was monitored via HPLC-MS. The brown, cloudy suspension was finally heated to 80 °C to inactivate the enzyme. After 2 h the suspension was allowed to cool to r. t.. The suspension was transferred to a separating funnel and extracted with ethyl acetate (2 × 20 mL) to remove unconverted aldehyde and other non-amino acid impurities. The aqueous phase was concentrated by rotary evaporation. The product (2*S*)-**3r** was purified via reversed-phase flash chromatography (60 g silica gel C18, 10.5 × 3 cm, H_2O/CH_3OH = 98:2 + 0.1 % formic acid (fraction 1 – 3), 80:20 + 0.1 % formic acid (fraction 4 – 20), 75:25 + 0.1 % formic acid (fraction 21 – 25), 100 mL

fractions (fraction 1 – 2), 50 mL fractions (fraction 3 – 25)). Fractions 7 - 12 were collected and the solvent was removed under reduced pressure.

Yield: 31 mg (126 µmol, 4 %), colorless solid.

$C_{13}H_{14}N_2O_3$ [246.10 g/mol]

mp = 170 - 178 °C (dec.)

HPLC-MS (method_2): t_{R1} = 3.57 min, t_{R2} = 4.02 min.

^{1}H-NMR (300.36 MHz, D_2O): δ = 9.06 (d, $^3J_{HH}$ = 8.7 Hz, 1H, H_{Ar}), 8.27 (t, $^3J_{HH}$ = 9.4 Hz, 2H, H_{Ar}), 8.15 – 8.06 (m, 2H, H_{Ar}), 8.02 (d, $^3J_{HH}$ = 8.6 Hz, 1H, H_{Ar}), 7.91 (d, $^3J_{HH}$ = 7.3 Hz, 1H, H_{Ar}), 5.61 (s, 1H, -C<u>H</u>OH), 1.71 (s, 3H, -CC<u>H</u>$_3$NH$_2$).

^{13}C-NMR (75.53 MHz, D_2O): δ = 170.4 (C_q, -<u>C</u>OOH), 165.6 (-<u>C</u>CH$_3$NH$_2$), 154.3 (-<u>C</u>HOH), 147.5 (C_{Ar}), 135.8 (C_{Ar}), 130.5 (C_q, C_{Ar}), 129.3 (C_{Ar}), 128.9 (C_{Ar}), 120.8 (C_{Ar}), 120.0 (C_{Ar}), 72.8 (C_q, C_{Ar}), 64.0 (C_q, C_{Ar}), 19.5 (-C<u>C</u>H$_3$NH$_2$).

HRMS (MALDI-TOF): Calcd. for $C_{13}H_{14}N_2O_3H$ $[M+H]^+$: 247.1083; found: 214.1087.

<u>(2*S*)-2-Amino-3-hydroxy-2-methyloctanoic acid ((2*S*)-**3u**, Table 3, entry 14):</u>

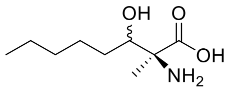

In a 100 mL round bottom flask, 2.67 g (30.0 mmol, 10.0 eq) DL-alanine **2b** was dissolved in 3 mL 100 mM KPi buffer pH 8.0 and 23.2 mL dist. H_2O. Under stirring, 300 µL 10 mM PLP and 1.5 mL isopropanol were added. The yellow solution was stirred at r. t. for 10 min. Then 1 mL L-TA was added. 365 µL (3.0 mmol, 1.0 eq) hexanal **1u** were added under stirring in 4 portions over 6 h. After 20 h, another 1 mL of L-TA lysate was added. The suspension was stirred at r. t. for 5 d. The conversion was monitored via HPLC-MS. The yellow, cloudy suspension was finally heated to 90 °C to inactivate the enzyme. After 45 min, the suspension was allowed to cool to r. t. The suspension was concentrated by rotary evaporation. The product (2*S*)-**3u** was purified via reversed-phase flash chromatography (60 g silica gel C18, 10.5 × 3 cm, H_2O/CH_3OH = 98:2 + 0.01 % formic acid (fraction 1 – 2), 96:4 + 0.01 % formic acid (fraction 3), H_2O/CH_3CN 50:50 + 0.01 % formic acid (fraction 4 - 7), 100 mL fractions). Fraction 4 was collected and the solvent was removed under reduced pressure. A short second flash chromatography using normal phase conditions (4 g silica gel, 7 × 1 cm, CH_2Cl_2/ CH_3OH = 75:25 + 5 % NH_3, 3 mL fractions) was required to remove further polar impurities. Fractions 6 - 20 were collected and the solvent was removed under reduced pressure.

Yield: 57 mg (300 µmol, 10 %), colorless solid.

$C_9H_{19}NO_3$ [189.14 g/mol]

$[\alpha]_D^{25}$ = 4.0 (c = 0.2 in 1 M HCl)

mp = 205 - 245 °C (dec.)

HPLC-MS (method_2): t_{R1} = 3.97 min, t_{R2} = 4.31 min.

^{1}H-NMR (300.36 MHz, D_2O): δ = 3.98 (d, $^3J_{HH}$ = 8.9 Hz, 1H, -C$\underline{H}$OH, syn), 3.82 (d, $^3J_{HH}$ = 7.5 Hz, 1H, -C$\underline{H}$OH, *anti*), 1.59 – 1.57 (m, 2H, -C$\underline{H}_2$CH$_2$CH$_2$CH$_2$CH$_3$), 1.56 (s, 3H, -CC$\underline{H}_3$NH$_2$, *anti*), 1.48 (s, 3H, -CC$\underline{H}_3$NH$_2$, *syn*), 1.29 (m, 12H, -CH$_2$C$\underline{H}_2$C$\underline{H}_2$C$\underline{H}_2$CH$_3$), 0.85 (s, 3H, -CH$_2$CH$_2$CH$_2$CH$_2$C$\underline{H}_3$), 0.84 (s, 3H, -CH$_2$CH$_2$CH$_2$CH$_2$C$\underline{H}_3$).

^{13}C-NMR (75.53 MHz, D_2O): δ = 173.2 (C_q, -$\underline{C}$OOH), 172.5 (C_q, -$\underline{C}$OOH), 73.3 (-C$\underline{H}$OH), 72.6 (-C$\underline{H}$OH), 64.3 (C_q, -$\underline{C}$CH$_3$NH$_2$), 64.1(C_q, -$\underline{C}$CH$_3$NH$_2$), 30.5 (-CH$_2$CH$_2$$\underline{C}H_2CH_2CH_3$), 30.4 (-CH$_2CH_2$$\underline{C}H_2CH_2CH_3$), 29.4 (-$\underline{C}H_2CH_2CH_2CH_2CH_3$), 25.1 (-CH$_2$$\underline{C}H_2CH_2CH_2CH_3$), 24.8 (-CH$_2$$\underline{C}H_2CH_2CH_2CH_3$), 21.8 (-CH$_2CH_2CH_2$$\underline{C}H_2CH_3$), 21.7 (-CH$_2CH_2CH_2$$\underline{C}H_2CH_3$), 19.1 (-C$\underline{C}H_3NH_2$), 16.7 (-C$\underline{C}H_3NH_2$), 13.2 (-CH$_2CH_2CH_2CH_2$$\underline{C}H_3$).

HRMS (MALDI-TOF): Calcd. for $C_9H_{19}NO_3H$ $[M+H]^+$: 190.1443; found: 190.1444.

<u>(2*R*)-2-Amino-3-hydroxy-2-methyloctanoic acid ((2*R*)-**3u**, Table 3, entry 15):</u>

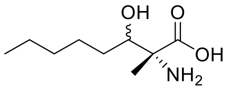

In a 100 mL round bottom flask, 2.67 g (30 mmol, 10.0 eq) DL-alanine **2b** was dissolved in 3 mL 100 mM KPi buffer pH 8.0 and 23.2 mL dist. H_2O. Under stirring, 300 µL 10 mM PLP, 60 µL 100 mM $MnCl_2$ (250 µM in reaction) and 1.5 mL isopropanol were added. The yellow solution was stirred at 15 °C for 15 min. Then 90 µL hexanal **1u** and 0.5 mL D-TA were added. The reaction mixture was stirred at 15 °C. After 3.5 h, another 90 µL hexanal **1u** were added. The reaction mixture was shaken at 120 rpm at 17.5 – 18.5 °C. After 20 h, further 185 µL aldehyde **1u** (to a total amount of 365 µL, 3.0 mmol, 1.0 eq) and 0.5 mL D-TA were added and the suspension further shaken at 17.5 – 18.5 °C for 4 d. The conversion was monitored via HPLC-MS. To prepare for the purification, the suspension was concentrated by rotary evaporation. The product (2*R*)-**3u** was purified via reversed-phase flash chromatography (60 g silica gel C18, 10.5 × 3 cm, H_2O/ CH_3OH = 98:2 + 0.01 % formic acid (fraction 1 – 2), 85:15 + 0.01 % formic acid (fraction 3 - 8), 80:20 + 0.01 % formic acid (fraction 9 - 16), 100 mL fractions (fraction 1 – 2), 50 mL fractions (fraction 3 – 16)). Fractions 8 - 14 were collected and the solvent was removed under reduced pressure.

Yield: 153 mg (808 µmol, 27 %), colorless solid.

$C_9H_{19}NO_3$ [189.14 g/mol]

mp = 221 - 235 °C (dec.)

$[\alpha]_D^{25}$ = -36.3 (c = 0.2 in 1 M HCl)

R_f = 0.34 (CH_2Cl_2/CH_3OH /NH_3 = 10/10/1, UV und Ninhydrin)

HPLC-MS (method_2): t_{R1} = 4.03 min, t_{R2} = 4.66 min.

^{1}H-NMR (300.36 MHz, D_2O): δ = 3.96 (d, $^3J_{HH}$ = 9.2 Hz, 1H, -C<u>H</u>OH, *syn*), 1.57 (m, 2H, -C<u>H</u>$_2$CH$_2$CH$_2$CH$_2$CH$_3$, *syn*), 1.46 (s, 3H, -CC<u>H</u>$_3$NH$_2$, *syn*), 1.30 (m, 6H, -CH$_2$C<u>H</u>$_2$C<u>H</u>$_2$C<u>H</u>$_2$CH$_3$), 0.84 (m, 3H, -CH$_2$CH$_2$CH$_2$CH$_2$C<u>H</u>$_3$, *syn*).

^{13}C-NMR (75.53 MHz, D_2O): δ = 173.1 (C_q, -<u>C</u>OOH), 72.6 (-C<u>H</u>OH), 64.3 (C_q, -<u>C</u>CH$_3$NH$_2$), 30.5 (-CH$_2$CH$_2$<u>C</u>H$_2$CH$_2$CH$_3$), 29.4 (-CH$_2$<u>C</u>H$_2$CH$_2$CH$_2$CH$_3$), 24.8 (-<u>C</u>H$_2$CH$_2$CH$_2$CH$_2$CH$_3$), 21.8 (-CH$_2$CH$_2$CH$_2$<u>C</u>H$_2$CH$_3$), 16.7 (-C<u>C</u>H$_3$NH$_2$), 13.2 (-CH$_2$CH$_2$CH$_2$CH$_2$<u>C</u>H$_3$).

HRMS (MALDI-TOF): Calcd. for $C_9H_{19}NO_3H$ $[M+H]^+$: 190.1443; found: 190.1443.

<u>(2*R*)-2-Amino-5-(((benzyloxy)carbonyl)amino)-3-hydroxy-2-methyl-pentanoic acid ((2*R*)-**3x**, Table 3, entry 16):</u>

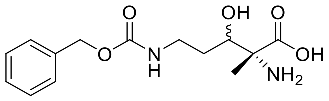

In a 100 mL round bottom flask, 1.34 g (15 mmol, 10.0 eq) DL-alanine **2b** was dissolved in 1.5 mL 100 mM KPi buffer pH 8.0 and 10.85 mL dist. H_2O. Under stirring, 150 µL 10 mM PLP and 30 µL 100 mM $MnCl_2$ (250 µM in reaction) were added. The yellow solution was stirred at 15 °C for 15 min. 0.25 mL D-TA were added. Then a solution of 311 mg (1.5 mmol, 1 eq.) benzyl (3-oxopropyl)carbamate **1x** in 0.5 mL isopropanol was added over a period of 10 h. The reaction mixture was shaken at 120 rpm at 17.5 – 18.5 °C. After 20 h, further 0.25 mL D-TA was added. The suspension was shaken at 17.5 – 18.5 °C for 2 d. The conversion was monitored via HPLC-MS. 85 mL methanol was finally added to stop the reaction and the mixture was kept stirring for 18 h. The precipitate was removed by filtration and washed with methanol (3 × 15 mL). The filtrate was concentrated by rotary evaporation and the product (2*R*)-**3x** was purified via reversed-phase flash chromatography (60 g silica gel C18, 10.5 × 3 cm, H_2O/CH_3OH = 98:2 + 0.01 % formic acid (fraction 1 – 2), 90:10 + 0.01 % formic acid (fraction 3 - 15), 85:15 + 0.01 % formic acid (fraction 16 - 28), 100 mL fractions (fraction 1 – 2), 50 mL fractions (fraction 3 – 16)). Fractions 19 - 28 were collected and the solvent was removed under reduced pressure.

Yield: 140 mg (473 µmol, 32 %), colorless solid.

$C_{14}H_{20}N_2O_5$ [296.14 g/mol]

$[\alpha]_D^{25}$ = -43.3 (c = 0.2 in 1 M HCl)

mp = 180 - 193 °C (dec.)

HPLC-MS (method_2): t_{R1} = 4.54 min, t_{R2} = 4.61 min.

^{1}H-NMR (300.36 MHz, D_2O): δ = 7.38 (s, 5H, H_{Ar}), 5.08 (s, 2H, -COOC$\underline{H}_2$Ph), 4.02 (d, $^{3}J_{HH}$ = 10.4 Hz, 1H, -C$\underline{H}$OH), 3.28 – 3.17 (m, 2H, -CH_2C$\underline{H}_2$NH-), 1.90 – 1.80 (m, 1H, -C$\underline{H}_2$$CH_2$NH-), 1.53 – 1.47 (m, 1H, -C$\underline{H}_2$$CH_2$NH-), 1.42 (s, 3H, -CC$\underline{H}_3$$NH_2$).

^{13}C-NMR (75.53 MHz, D_2O): δ = 172.8 (C_q, -$\underline{C}$OOH), 158.5 (C_q, -NH$\underline{C}$OOCH$_2$-), 136.5 (C_q, C_{Ar}), 128.8 (C_{Ar}), 128.4 (C_{Ar}), 127.6 (C_{Ar}), 67.0 (-$\underline{C}$HOH), 66.9 (-COO$\underline{C}$H$_2$Ph), 64.2 (C_q, -$\underline{C}$CH$_3$NH$_2$), 37.1 (-CH$_2$$\underline{C}H_2$NH-), 29.8 (-$\underline{C}H_2CH_2$NH-), 16.8 (-C$\underline{C}H_3NH_2$).

HRMS (MALDI-TOF): Calcd. for $C_{14}H_{20}N_2O_5H$ $[M+H]^+$: 297.1451; found: 214.1459.

<u>(*S*)-2-Amino-2-methyl-3-phenylpropanoic acid ((*S*)-**4a**):</u>

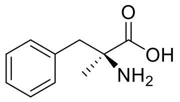

A flame-dried and N_2-flushed 100 mL three-neck round-bottom flask equipped with magnetic stirring bar, dry-ice condenser, and gas-bubbler was charged with 150 mg (624 µmol, 1.0 eq.) 2-amino-3-hydroxy-2-methyl-3-phenylpropanoic acid. The system was then cooled to -78 °C (dry ice/acetone) and 20 mL NH_3 were condensed into the flask. Afterwards, 16 mg Li (2.31 mmol, 3.00 equiv.) were slowly added and stirring continued for 3 h. Then the dark blue suspension was quenched by the addition of 10 mL H_2O and concentrated by rotary evaporation. The product <u>(*S*)-**4a**</u> was purified via flash chromatography (20 g silica gel, 11 x 2 cm, CH_2Cl_2/CH_3OH/NH_4OH = 10:8:1, 8 mL fractions). Fractions 17 - 26 were collected and the solvent was removed under reduced pressure.

Yield: 126 mg (704 µmol, 91 %), colorless solid.

$C_{10}H_{13}NO_2$ [179,09 g/mol]

mp = 195 - 212 °C (dec.)

HPLC-MS (method_3): t_R = 4.34 min

^{1}H-NMR (300.36 MHz, DMSO-d_6): δ = 7.25 (m, 3H, H_{Ar}), 7.16 (m, 2H, H_{Ar}), 3.12 (d, $^{3}J_{HH}$ = 13.9 Hz, 1H, -C$\underline{H}_2$-Ph), 2.80 (d, $^{3}J_{HH}$= 13.9 Hz, 1H, -C$\underline{H}_2$-Ph), 1.32 (s, 1H, -CC$\underline{H}_3$NH$_2$).

^{13}C-NMR (APT) (75.53 MHz, DMSO-d_6): δ = 174.8 (C_q, -$\underline{C}$OOH), 135.9 (C_q, C_{Ar}), 131.3 (C_{Ar}), 129.7 (C_{Ar}), 128.4 (C_{Ar}), 62.2 (C_q, -$\underline{C}$CH$_3$NH$_2$), 43.5 (-$\underline{C}$H$_2$-), 23.7 (-C$\underline{C}$H$_3$NH$_2$).

1.6. Additional syntheses and related experimental procedures

1.6.1. Birch reductions of phenylserine derivatives

Birch reductions applied for the β-dehydroxylation of (2*S*)-amino-3-hydroxy-2-methyl-3-(2-nitrophenyl)propanoic acid (2*S*)-**(*m*)-3b** resulted in the formation of the cyclic cross-coupling product (2*S*)-**4b** (Scheme S1).

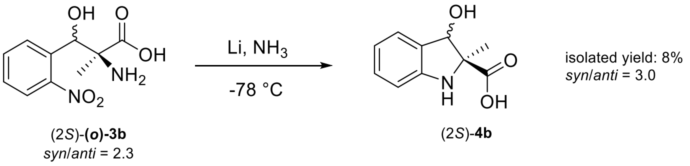

Scheme S1. Birch reduction of (2*S*)-**(*o*)-3b**

In the case of (2*S*)-2-amino-3-hydroxy-3-[3-(methoxycarbonyl)phenyl]-2-methyl-propanoic acid (2*S*)-**(*m*)-3j**, the Birch reduction accomplished the desired β-dehydroxylation but additionally resulted in a formal regioselective decarboxylative alkylation at the phenyl ring (Scheme S2).

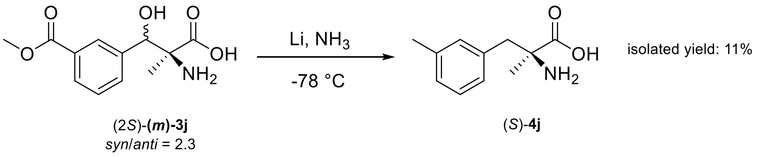

Scheme S2. Birch reduction of (2*S*)-**(*m*)-3j**

<u>(2*S*)-3-Hydroxy-2-methylindole-2-carboxylic acid (2*S*)-**4b**:</u>

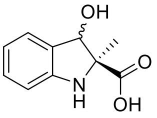

A flame-dried and N_2-flushed 100 mL three-neck round-bottom flask equipped with magnetic stirring bar, dry-ice condenser, and gas-bubbler was charged with 150 mg (624 µmol, 1.0 eq.) (2*S*)-2-amino-3-hydroxy-2-methyl-3-(2-nitrophenyl)propanoic acid (2*S*)-**(*m*)-3b**. The system was then cooled to -78 °C (dry ice/acetone) and 25 mL NH_3 were condensed into the flask. Afterwards, 34 mg Li (4.9 mmol, 7.85 equiv.) were slowly added and stirring continued for 4 h. Then the dark blue suspension was quenched by the addition of 20 mL H_2O and concentrated by rotary evaporation. The product (2*S*)-**4b** was purified via flash chromatography (5 g silica gel, 6.5 × 1 cm, CH_2Cl_2/CH_3OH = 70:30 + 5 % NH_3, 3 mL

fractions). Fractions 4 - 5 were collected and the solvent was removed under reduced pressure.

Yield: 11 mg (57 µmol, 9 %), yellow oil.

$C_{10}H_{11}NO_3$ [193.26 g/mol]

$[\alpha]_D^{25}$ = -6.0 (c = 0.3 in 1 M HCl)

HPLC-MS (method_2): t_{R1} = 4.44 min, t_{R1} = 4.66 min

^{1}H-NMR (300.36 MHz, D_2O): δ = 7.50 - 7.36 (m, 4H, H_{Ar}), 7.28 – 7.20 (m, 2H, H_{Ar}), 7.05 - 6.98 (m, 2H, H_{Ar}), 5.21 (s, 1H, -C<u>H</u>OH), 1.43 (s, 3H, -CC<u>H</u>$_3$NH$_2$, *anti*), 1.33 (s, 3H, -CC<u>H</u>$_3$NH$_2$, *syn*).

^{13}C-NMR (75.53 MHz, D_2O): δ = 169.2 (C_q, -<u>C</u>OOH), 165.7 (C_q, -<u>C</u>OOH), 133.1 (C_q, C_{Ar}), 130.9 (C_q, C_{Ar}), 129.9 (C_q, C_{Ar}), 129.5 (C_q, C_{Ar}),125.1 (C_{Ar}), 124.8 (C_{Ar}), 124.5 (C_{Ar}), 116.6 (C_{Ar}), 116.1 (C_{Ar}), 71.1 (-<u>C</u>HOH), 68.9 (-<u>C</u>HOH), 60.2 (C_q, -<u>C</u>CH$_3$NH$_2$), 18.2 (-C<u>C</u>H$_3$NH$_2$), 13.8 (-C<u>C</u>H$_3$NH$_2$).

<u>(*S*)-2-Amino-3-hydroxy-2-methyl-3-(m-tolyl)propanoic acid (*S*)-**4j**:</u>

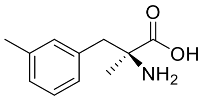

A flame-dried and N_2-flushed 100 mL three-neck round-bottom flask equipped with magnetic stirring bar, dry-ice condenser, and gas-bubbler was charged with 100 mg (395 µmol, 1.0 eq.) (2*S*)-2-amino-3-hydroxy-3-[3-(methoxycarbonyl)phenyl]-2-methyl-propanoic acid (2*S*)-**(*m*)-3j**. The system was cooled to -78 °C (dry ice/ acetone) and 25 mL NH_3 were condensed into the flask. Afterwards, 8.5 mg Li (1.2 mmol, 3.1 equiv.) were slowly added and stirring continued for 1 h. Additional 27 mg Li (3.9 mmol, 9.9 eq.) were slowly added. After altogether 4 h, the dark blue suspension was quenched by the addition of 15 mL H_2O and concentrated by rotary evaporation. The residue was dissolved in 1.5 mL H_2O/CH_3OH (1:2) and 300 µL formic acid and the product (*S*)-**4j** purified via reversed-phase preparative HPLC (method_7).

Yield: 8 mg (41 µmol, 11 %), colorless solid.

$C_{11}H_{15}NO_2$ [193.11 g/mol]

HPLC-MS (method_3): t_{R1} = 4.17 min

^{1}H-NMR (300.36 MHz, D_2O): δ = 7.0 (t, $^3J_{HH}$ = 7.5 Hz, 1H, H_{Ar}), 7.22 (d, $^3J_{HH}$ = 7.4 Hz, 1H, H_{Ar}), 7.11 – 7.06 (m, 2H, H_{Ar}), 3. 11 (dd, $^2J_{HH}$ = 14.2 Hz, 2H, -C<u>H</u>OH), 2.33 (s3 6H, -PhC<u>H</u>$_3$), 1.55 (s, 3H, -CC<u>H</u>$_3$NH$_2$).

^{13}C-NMR (75.53 MHz, D_2O): δ = 176.2 ($C_{q,}$ -C̲OOH), 139.1 (C_q, C_{Ar}), 134.3 (C_q, C_{Ar}), 130.7 (C_{Ar}), 128.9 (C_{Ar}), 128.5 (C_{Ar}), 127.0 (C_{Ar}), 62,2 ($C_{q,}$ -C̲CH_3NH_2), 42.6 (-C̲HOH), 22.4 (-CC̲H_3NH_2), 20.4 (-PhC̲H_3).

1.6.2. (*S*)-2-Amino-3-(2-chlorophenyl)-2-methylpropanoic acid (*S*)-6:

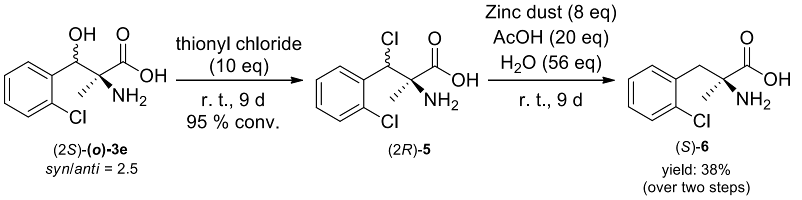

Scheme S3. Synthesis of (*S*)-2-amino-3-(2-chlorophenyl)-2-methylpropanoic acid (S)-6

The dehydroxylation of (2*S*)-**(*o*)-3e** was conducted by a two-step procedure comprising a conversion of the hydroxyl-group into the corresponding chloride and subsequent treatment with zinc under acidic conditions to generate (*S*)-**6**.

(2*R*)-2-Amino-3-chloro-3-(2-chlorophenyl)-2-methylpropanoic acid (2*R*)-**5**:

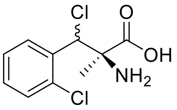

To a flame-dried 10 mL round-bottom flask with nitrogen inlet, 350 mg (1.5 mmol, 1.0 eq) (2*S*)-amino-3-(2-chlorophenyl)-3-hydroxy-2-methylpropanoic acid (2*S*)-**(*o*)-3e** and 1.1 mL (15.2 mmol, 10.0 eq) thionyl chloride were added. The formed orange/red oil was stirred at 60 °C for 18 h. The conversion was monitored via HPLC-MS. After 95 % conversion, the red oil was added dropwise to 2 mL H_2O and 3 mL acetone at 0 °C to hydrolyze thionyl and the amino acid chloride. The solvent was removed under reduced pressure using an evaporator and a cooling trap immersed in liquid nitrogen to avoid corrosive compounds entering the pump system. The collecting vessel was filled with 50 mL CH_3OH/H_2O (1:1) to dissolve HCl and quench residual thionyl chloride. The crude product was used without further purification in the next reaction.

$C_{10}H_{11}Cl_2NO_2$ [248.10 g/mol]

HPLC-MS (method_2): t_{R1} = 4.42 min, t_{R2} = 4.51 min.

(*S*)-2-Amino-3-(2-chlorophenyl)-2-methylpropanoic acid (*S*)-**6**:

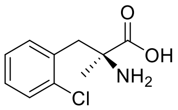

In a flame-dried 50 mL two-neck round-bottom flask with nitrogen inlet, 248 mg (1.0 mmol, 1.0 eq) 2-amino-3-chloro-3-(2-chlorophenyl)-2-methylpropanoic acid (2*R*)-**5** were dissolved in 1.2 mL (20.0 mmol, 20 eq) acetic acid and 1 mL dist. H_2O (56.0 mmol, 56 eq). 532 mg (8.0 mmol, 8.0 eq) zinc dust was added and the reaction mixture was stirred at r. t. for 2 d. 490 µL (18 eq) conc. HCl were added to the brown/grey suspension. The product (*S*)-**6** was purified without any work-up via reversed-phase flash-chromatography (60 g silica gel C18, 10.5 × 3 cm, H_2O/CH_3OH = 98:2 + 0.01 % formic acid (fraction 1 – 10), 90:10 + 0.01 % formic acid (fraction 11 - 21), 85:15 + 0.01 % formic acid (fraction 22 - 30), 50 mL fractions). Fractions 21 - 29 were collected and the solvent was removed under reduced pressure.

Yield: 82 mg (384 µmol, 38 %), colorless solid.

$C_{10}H_{12}ClNO_2$ [213.66 g/mol]

$[\alpha]_D^{25}$ = -6.3 (c = 0.2 in 1 M HCl)

mp = 175 - 196 °C (dec.)

HPLC-MS (method_2): t_{R1} = 3.37 min, t_{R2} = 3.65 min.

^{1}H-NMR (300.36 MHz, D_2O): δ = 7.49 (m, 1H, H_{Ar}), 7.34 (m, 3 H, H_{Ar}), 3.44 (dd, $^2J_{HH}$ = 14.6 Hz , 2 H, -C$\underline{H}_2$Ph), 1.66 (s, 1 H, -C$\underline{H}_3$$NH_2$).

^{13}C-NMR (75.53 MHz, D_2O): δ = 173.2 (-$\underline{C}$OOH), 123.6 (C_q, C_{Ar}), 132.4 (-$\underline{C}$Cl), 131.0 (C_{Ar}), 130.0 (C_{Ar}), 129.9 (C_{Ar}), 127.5 (C_{Ar}), 61.0 (C_q, -$\underline{C}CH_3NH_2$), 39.0 (-C$\underline{C}H_2$Ph), 21.4 (-C$\underline{C}H_3NH_2$).

HRMS (MALDI-TOF): Calcd. for $C_{10}H_{12}ClNO_2H$ $[M+H]^+$: 214.0635; found: 214.0637.

2. Literature

[1] a) I. Molnár-Perl, *J. Chromatogr. A* **2001**, *913*, 283-302; b) M. G. Chernobrovkin, E. N. Shapovalova, D. T. Guranda, P. A. Kudryavtsev, V. K. Švedas, O. A. Shpigun, *J. Chromatogr. A* **2007**, *1175*, 89-95.

[2] a) C. Reisinger, A. Kern, K. Fesko, H. Schwab, *Appl. Microbiol. Biotechnol.* **2007**, *77*, 241; b) K. Fesko, M. Uhl, J. Steinreiber, K. Gruber, H. Griengl, *Angew. Chem., Int. Ed.* **2010**, *49*, 121-124.

[3] R. O. Cann, C.-P. H. Chen, Q. Gao, R. L. Hanson, D. Hsieh, J. Li, D. Lin, R. L. Parsons, Y. Pendri, R. B. Nielsen, W. A. Nugent, W. L. Parker, S. Quinlan, N. P. Reising, B. Remy, J. Sausker, X. Wang, *Org. Proc. Res. Dev.* **2012**, *16*, 1953-1966.

3. NMR spectra

^{1}H-NMR of (2*S*)-**3a** (Table 3, entry 1)

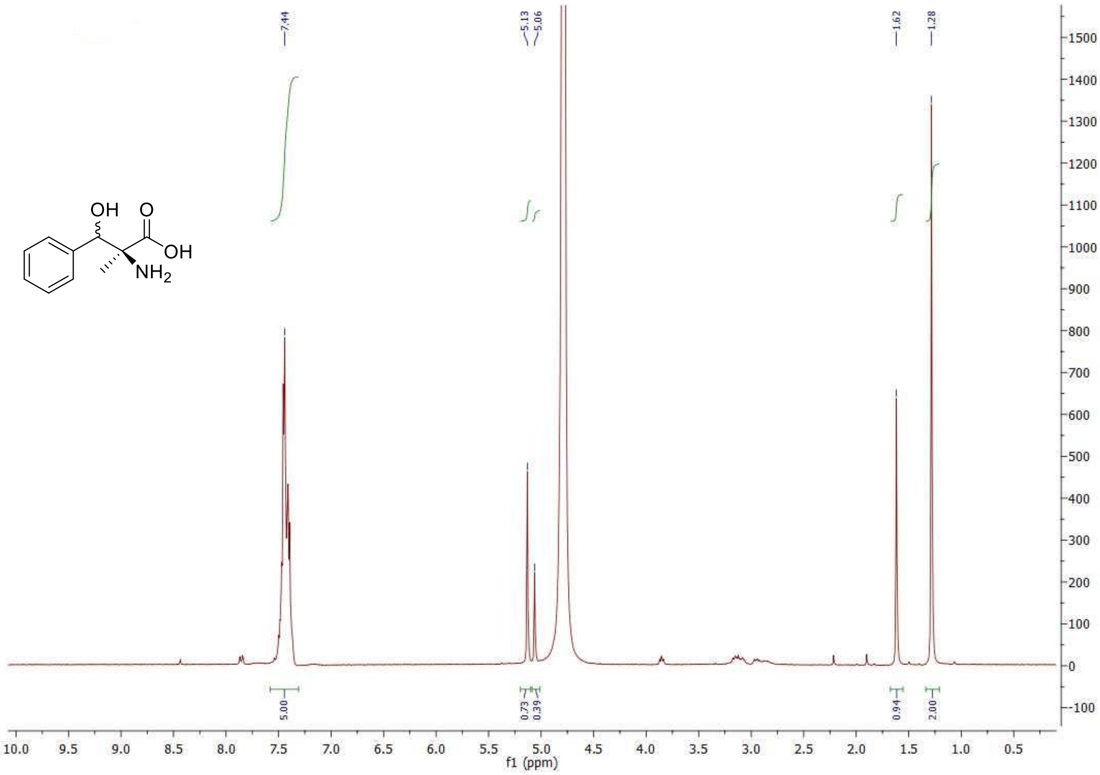

^{13}C-NMR of (2*S*)-**3a** (Table 3, entry 1)

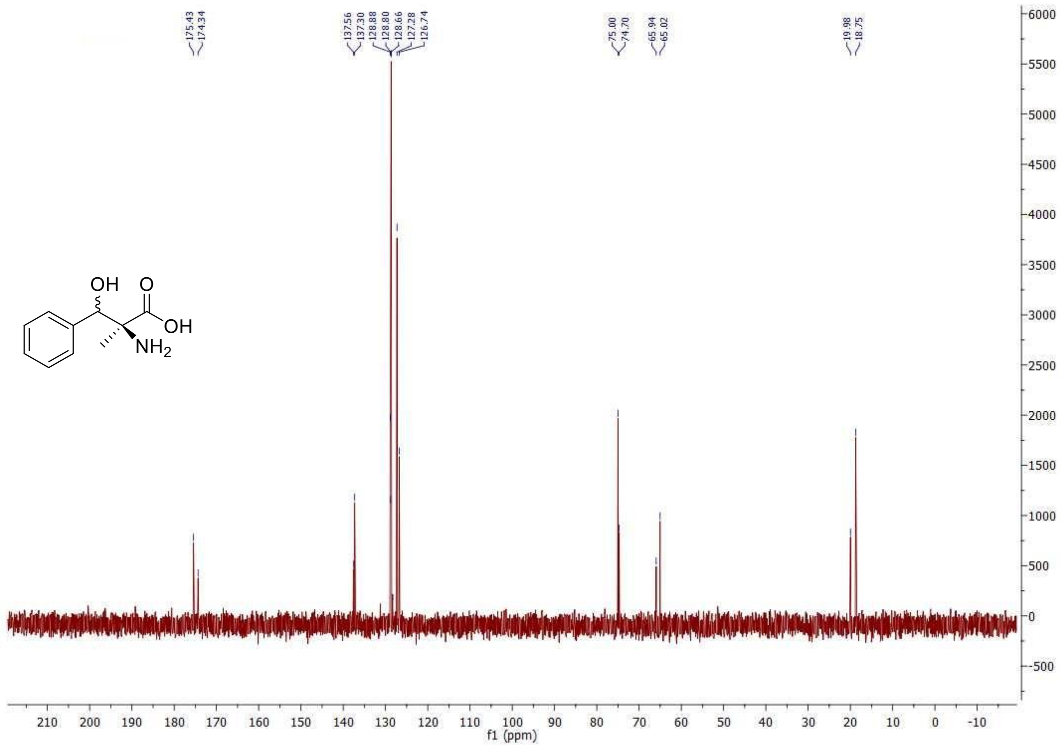

^{1}H-NMR of (2*R*)-**3a** (Table 3, entry 2)

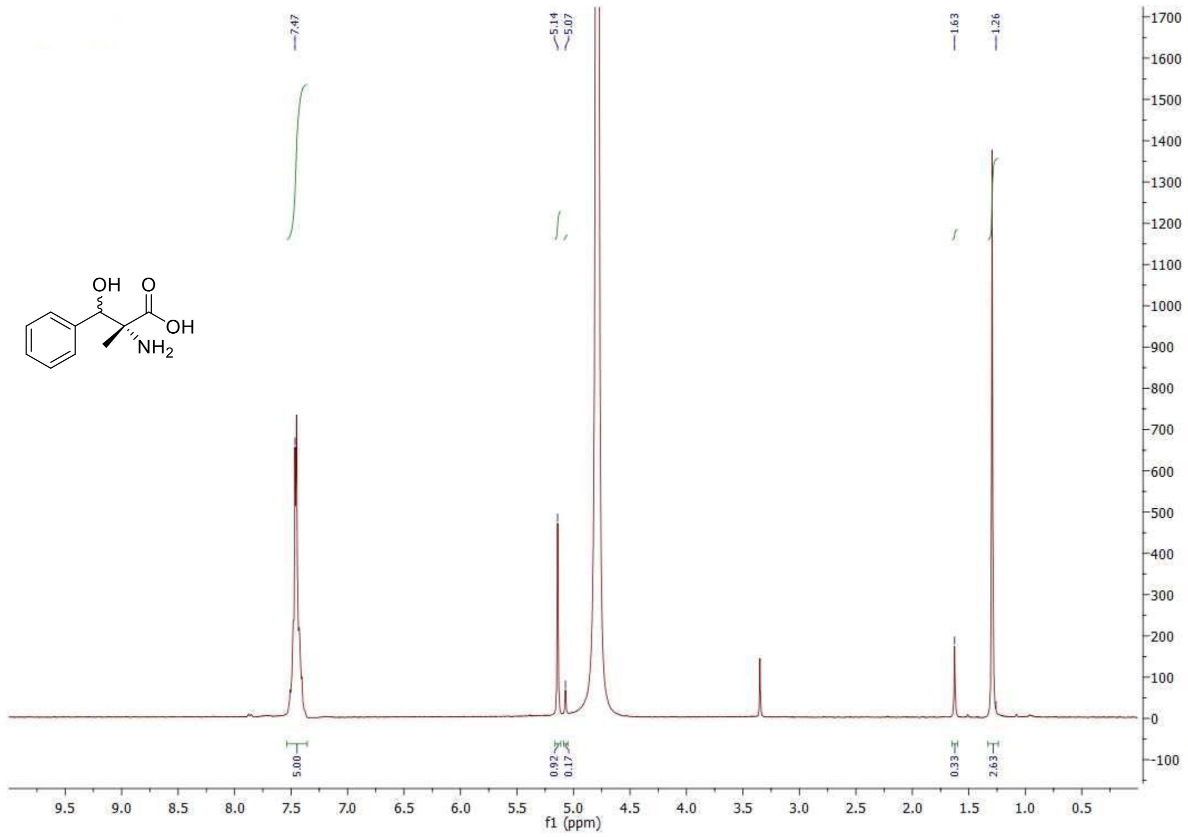

^{13}C-NMR of (2*R*)-**3a** (Table 3, entry 2)

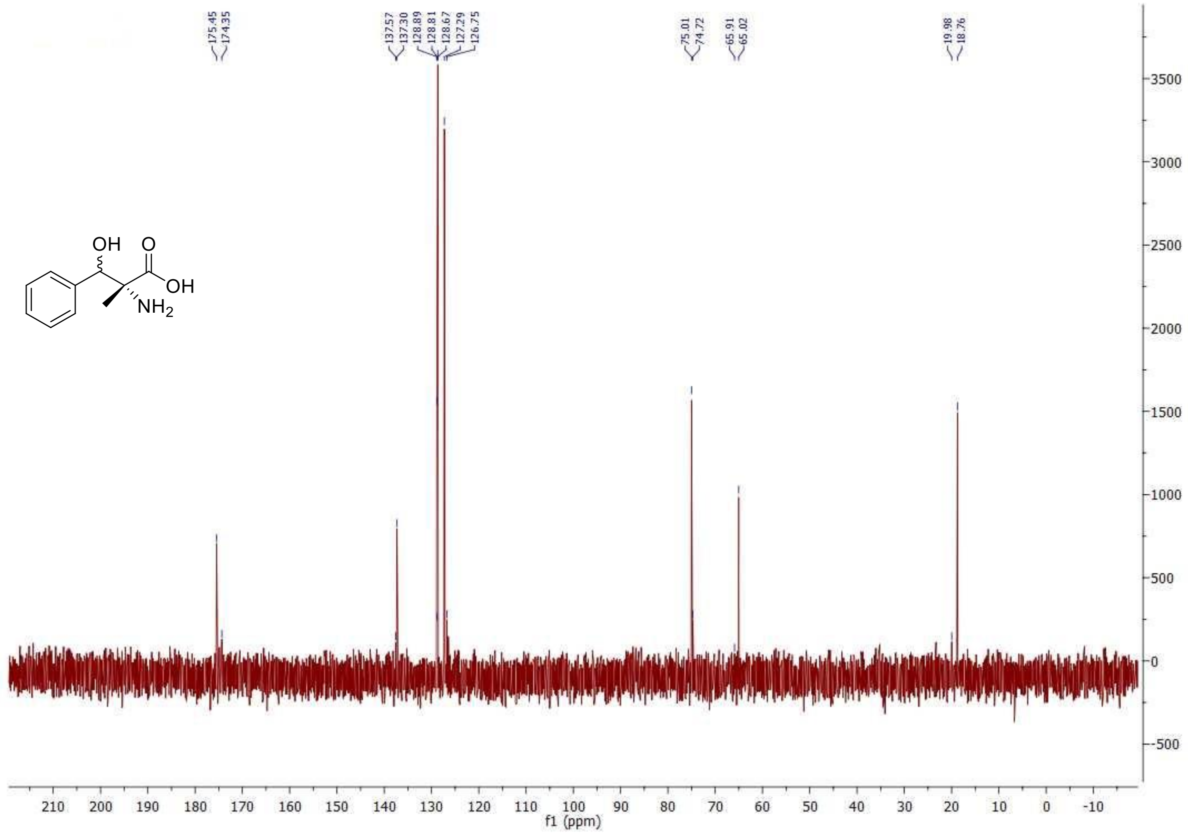

^{1}H-NMR of (2*S*)-**(*o*)-3b** (Table 3, entry 3)

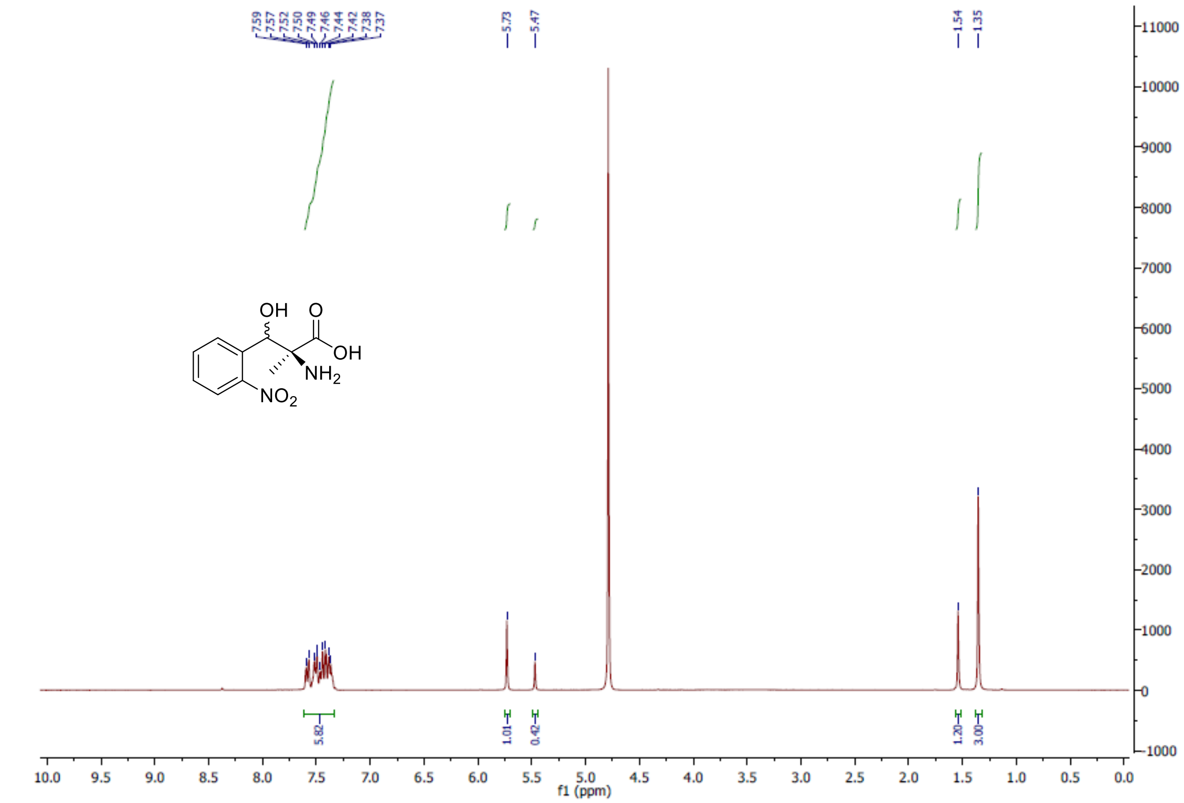

^{13}C-NMR of (2*S*)-**(*o*)-3b** (Table 3, entry 3)

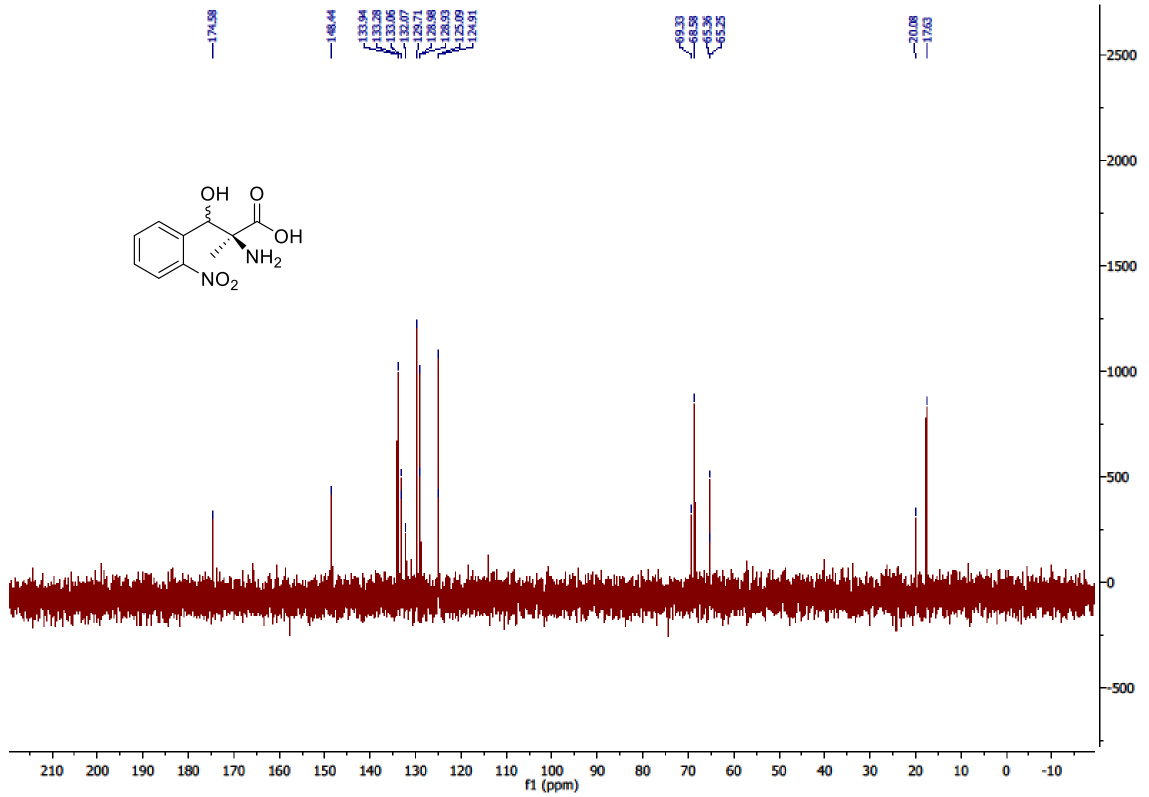

^{1}H-NMR of (2*S*)-**(*m*)-3b** (Table 3, entry 4)

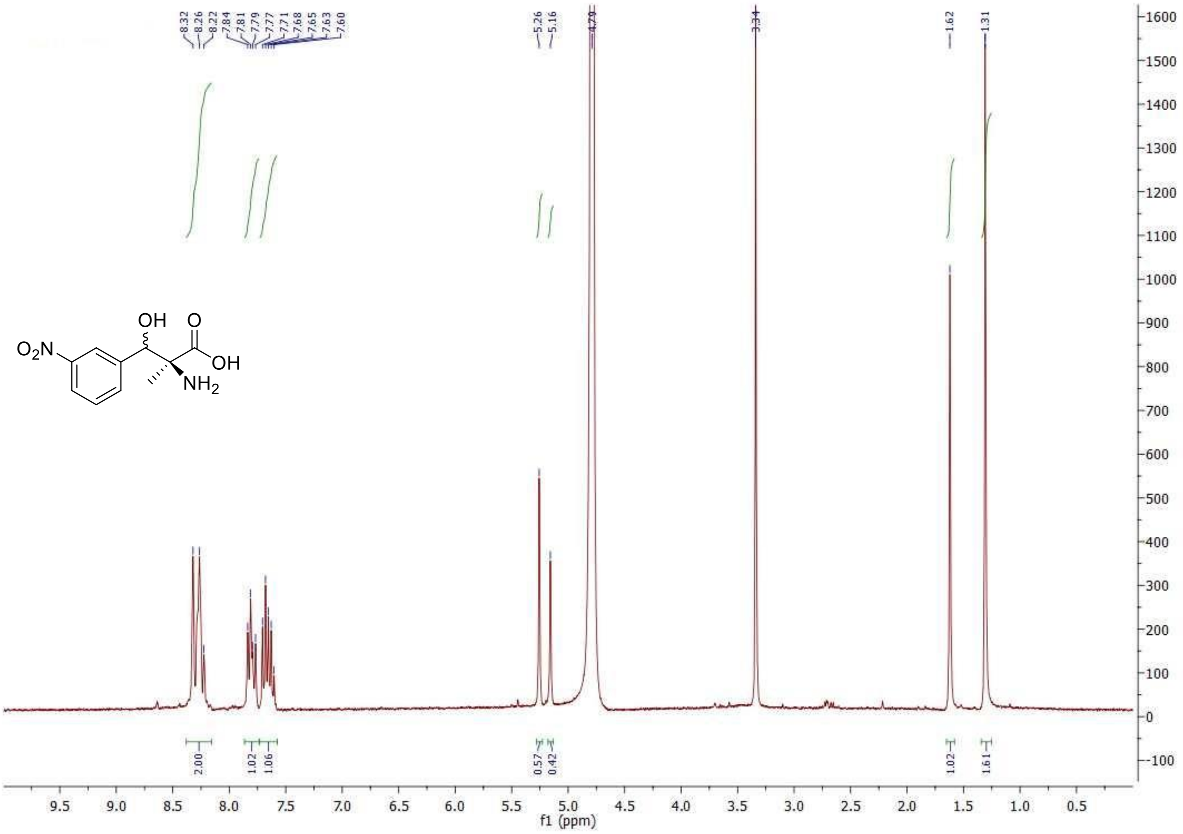

^{13}C-NMR of (2*S*)-**(*m*)-3b** (Table 3, entry 4) (2*S*)-**(*m*)-3b** (Table 3, entry 4)

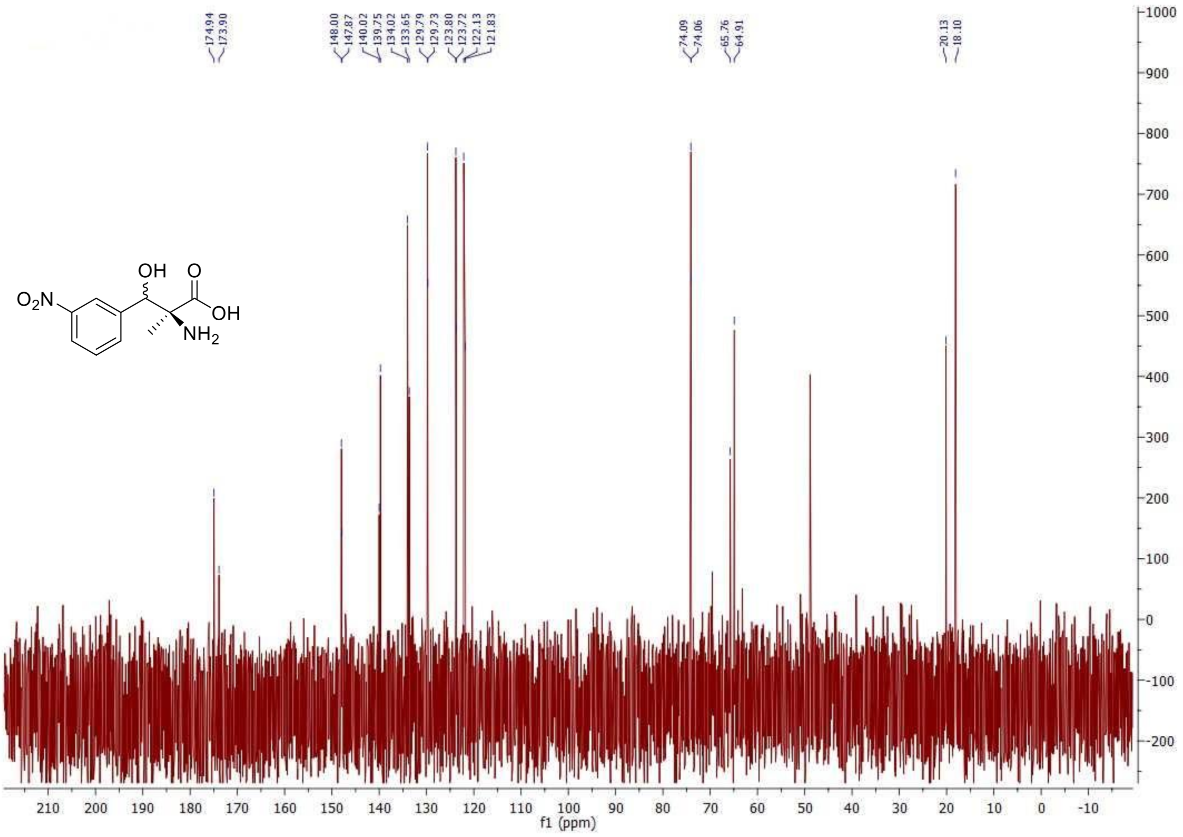

^{1}H-NMR of (2*S*)-**(*p*)-3b** (Table 3, entry 5)

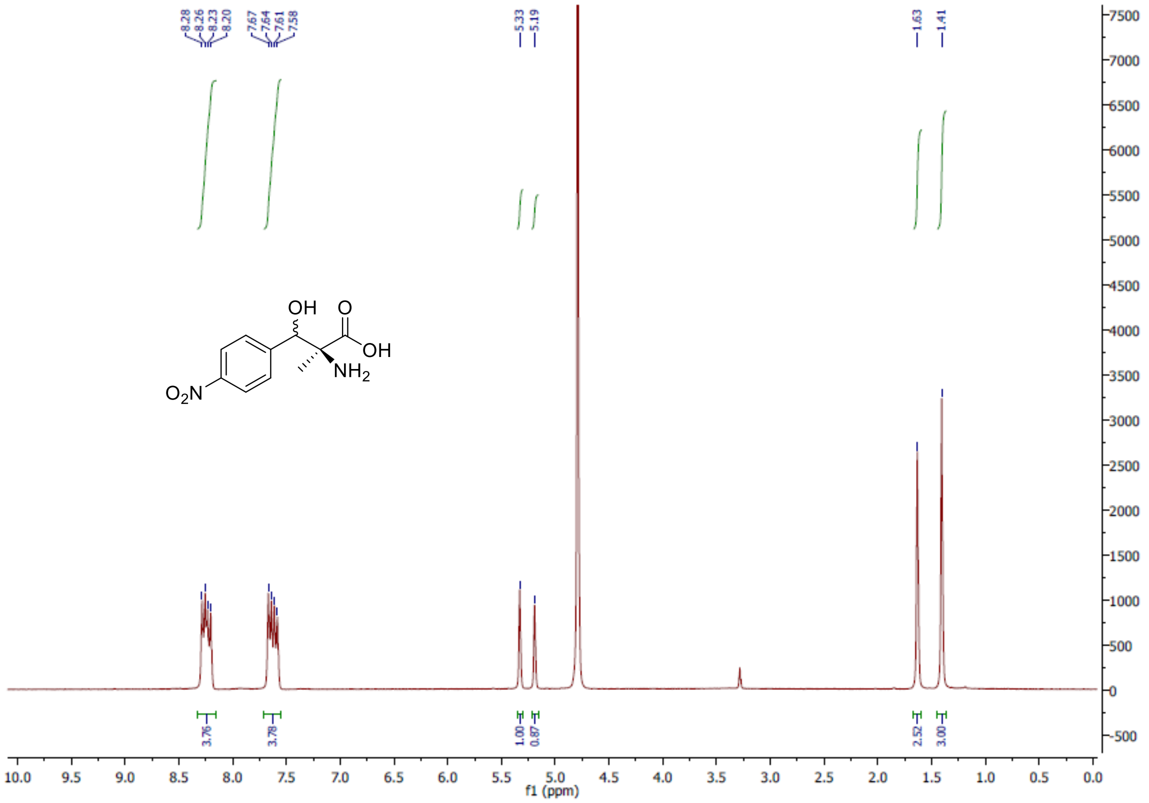

^{13}C-NMR of (2*S*)-**(*p*)-3b** (Table 3, entry 5)

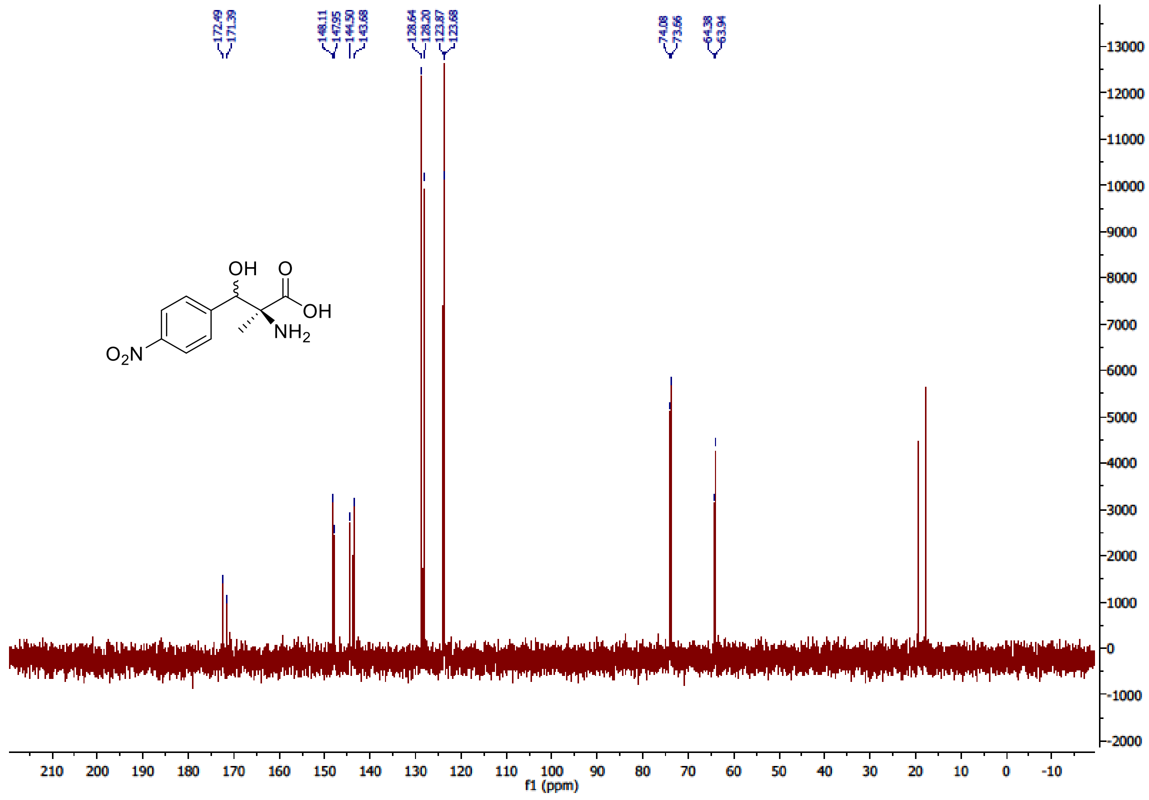

^{1}H-NMR of (2*S*)-**(*o*)-3e** (Table 3, entry 6)

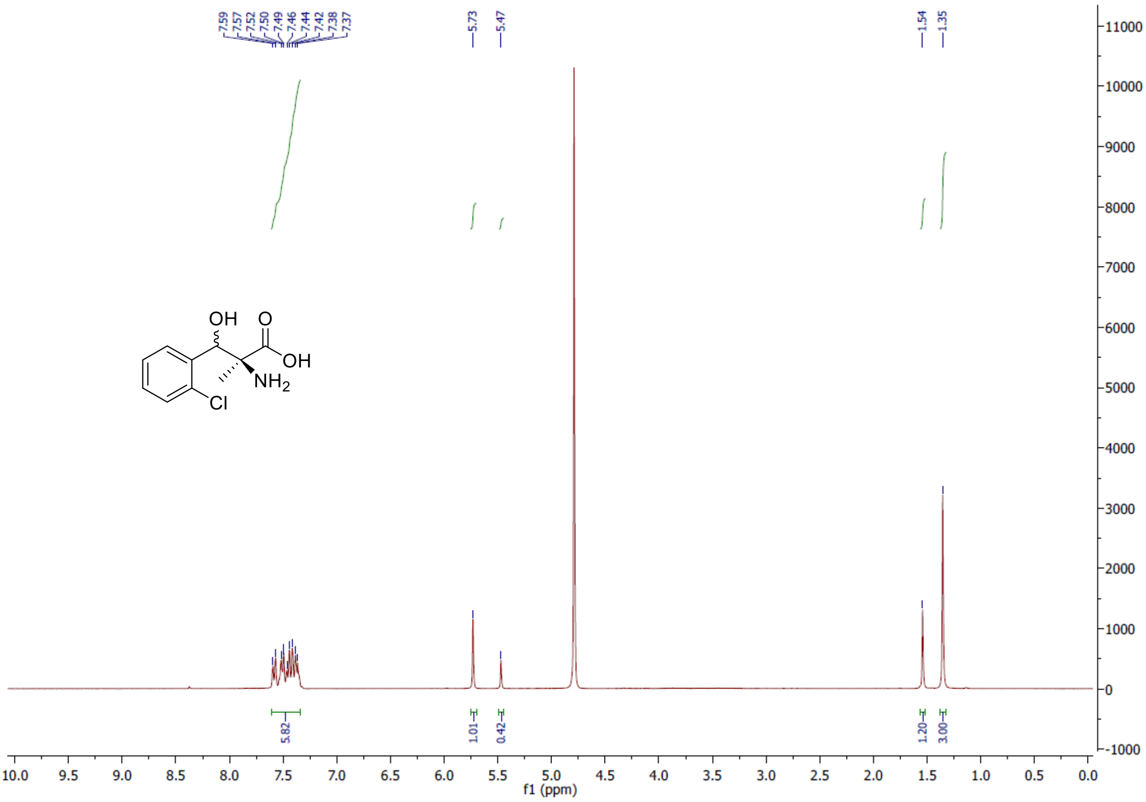

^{13}C-NMR of (2*S*)-**(*o*)-3e** (Table 3, entry 6)

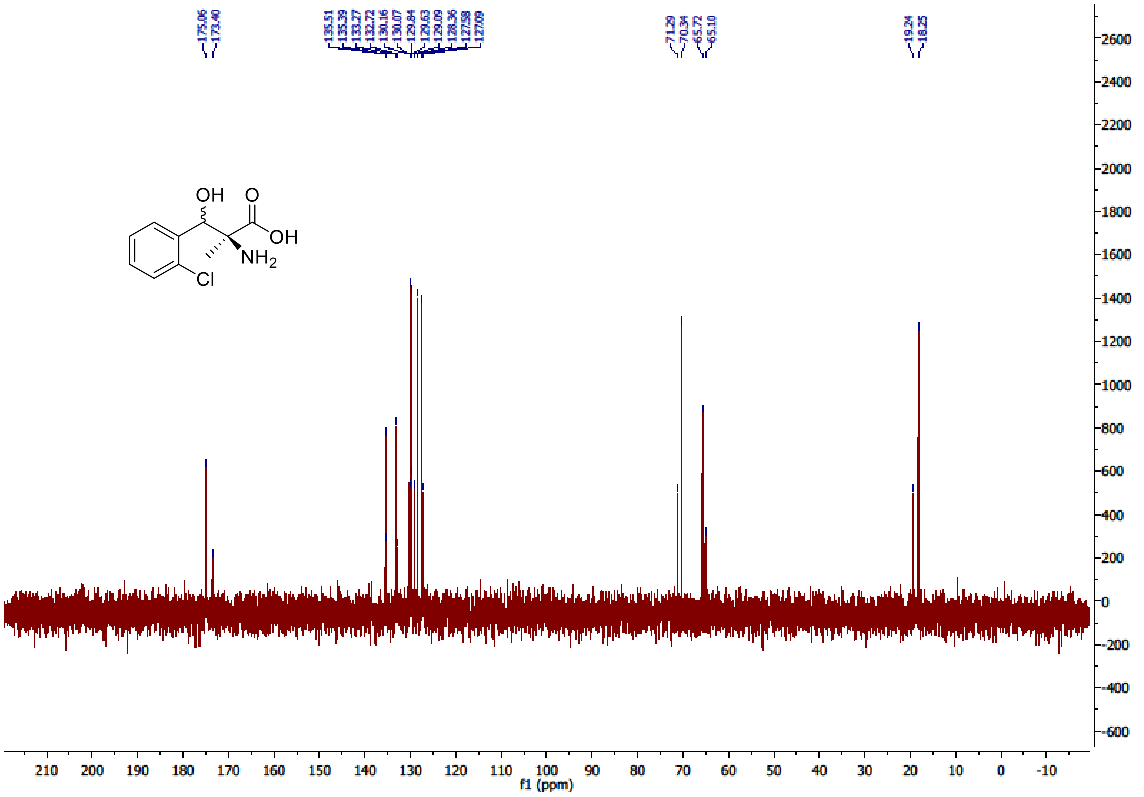

^{1}H-NMR of (2*S*)-**(*o*)-3f** (Table 3, entry 7)

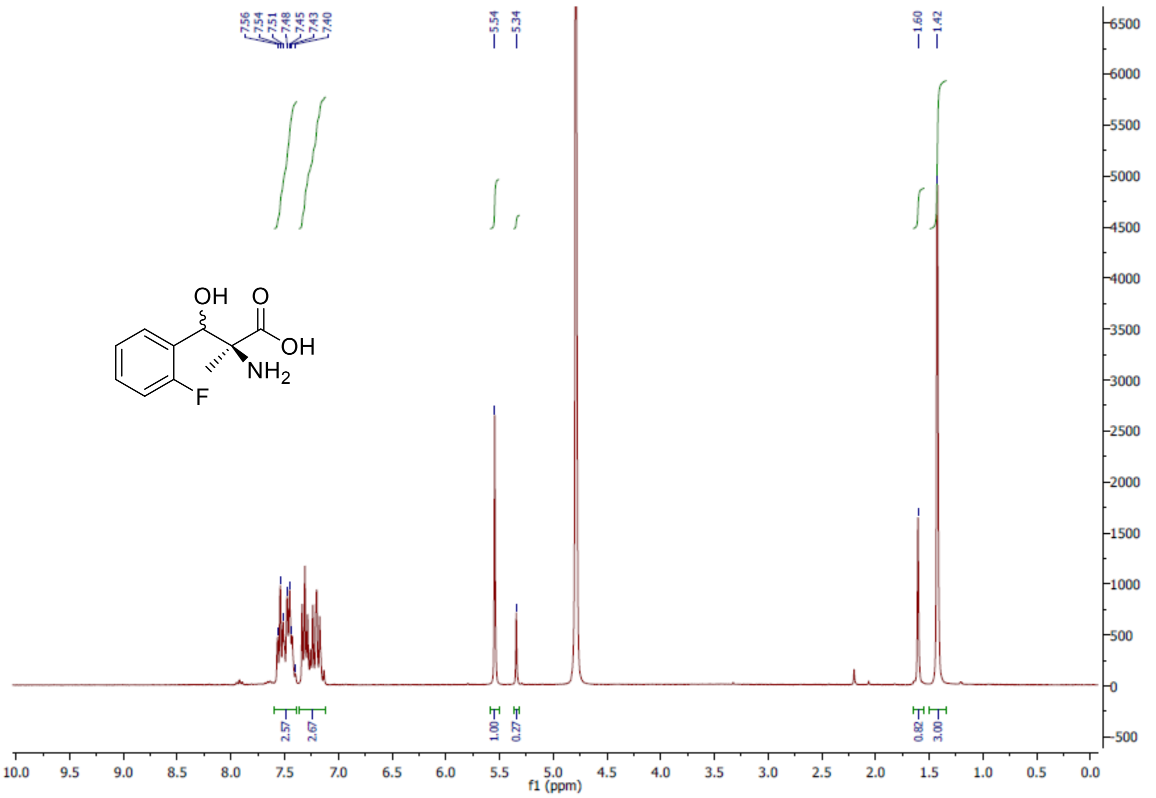

^{13}C-NMR of (2*S*)-**(*o*)-3f** (Table 3, entry 7)

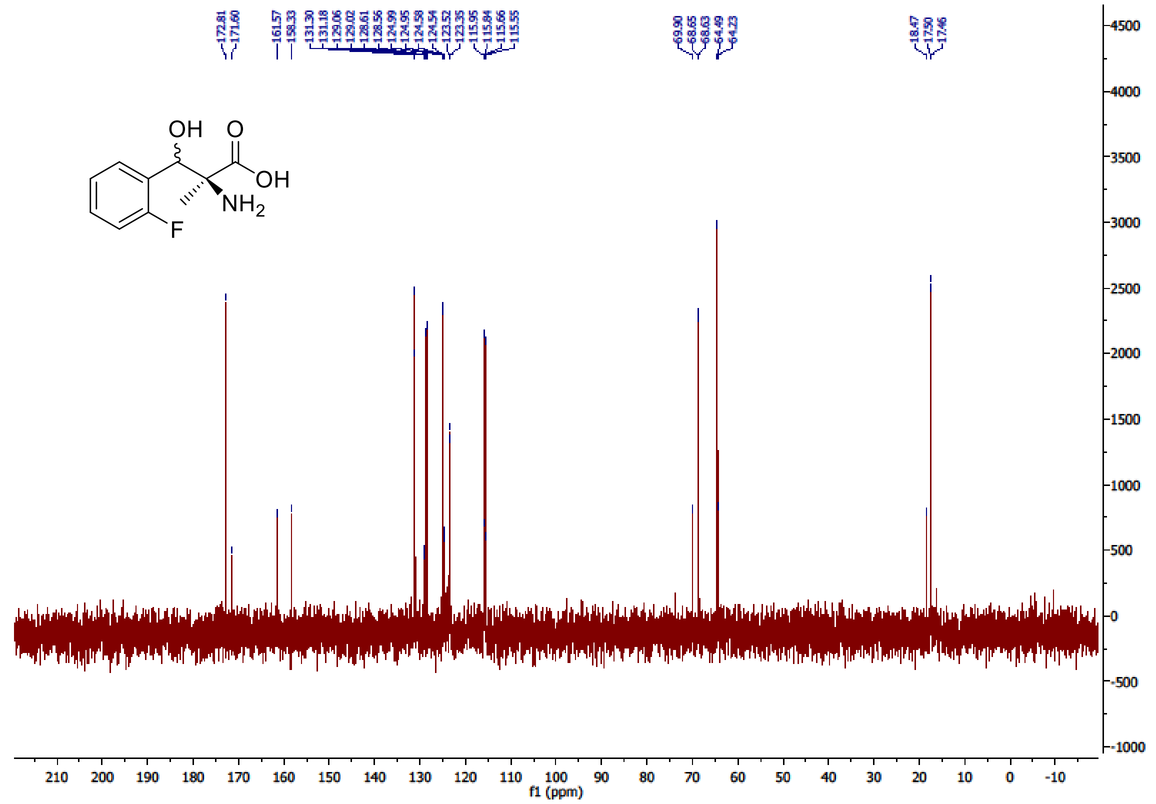

^{1}H-NMR of (2*R*)-**(*o*)-3f** (Table 3, entry 8)

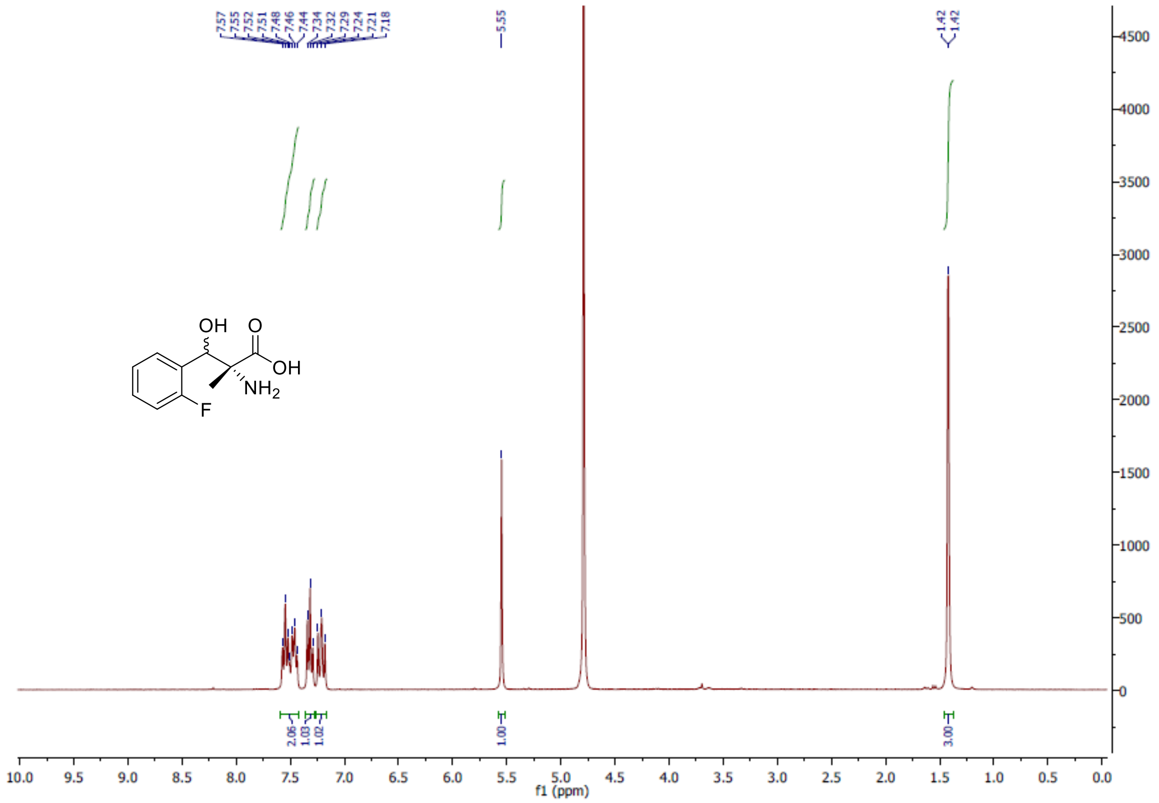

^{13}C-NMR of (2*R*)-**(*o*)-3f** (Table 3, entry 8)

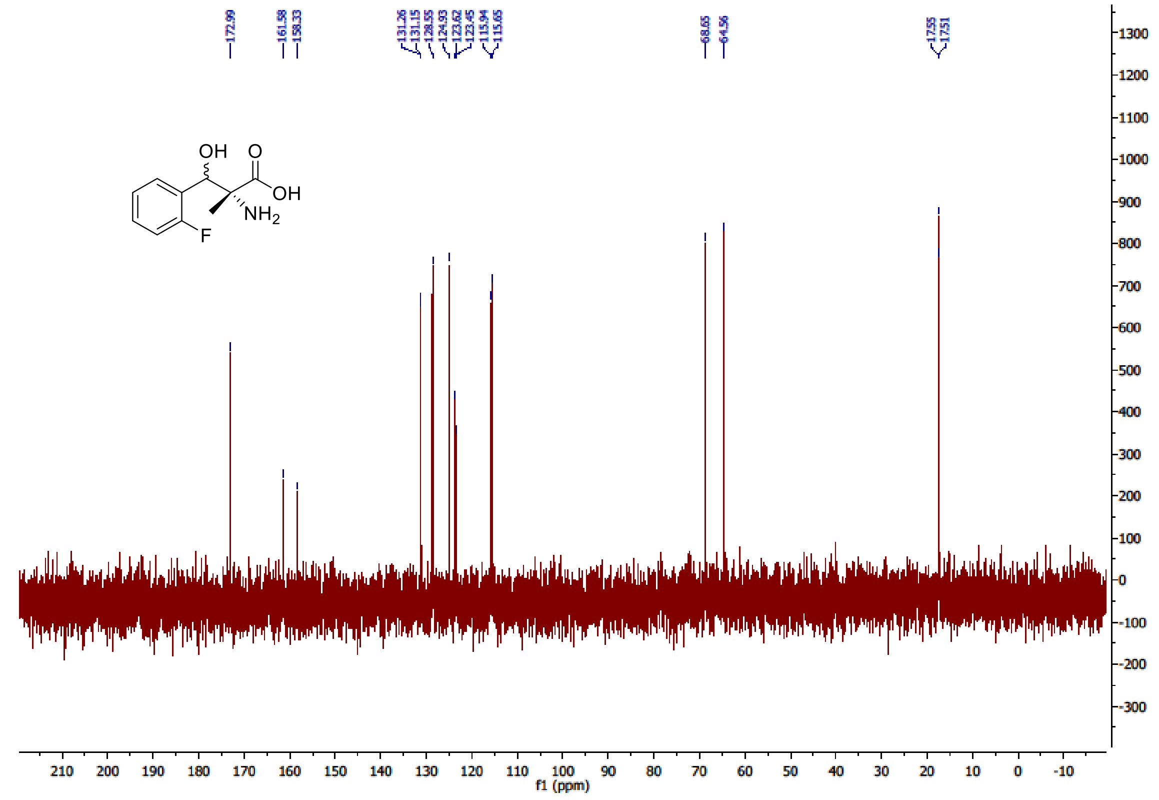

^{1}H-NMR of (2*S*)-**(*m*)-3i** (Table 3, entry 9)

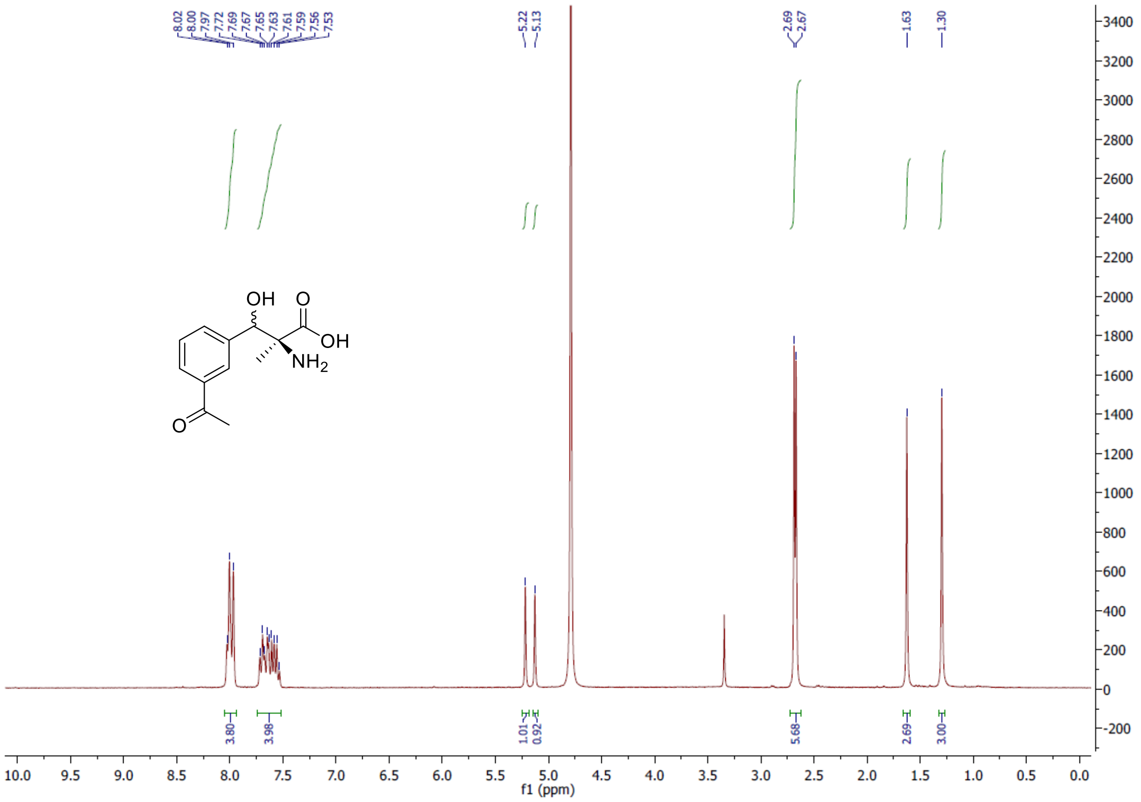

^{13}C-NMR of (2*S*)-**(*m*)-3i** (Table 3, entry 9)

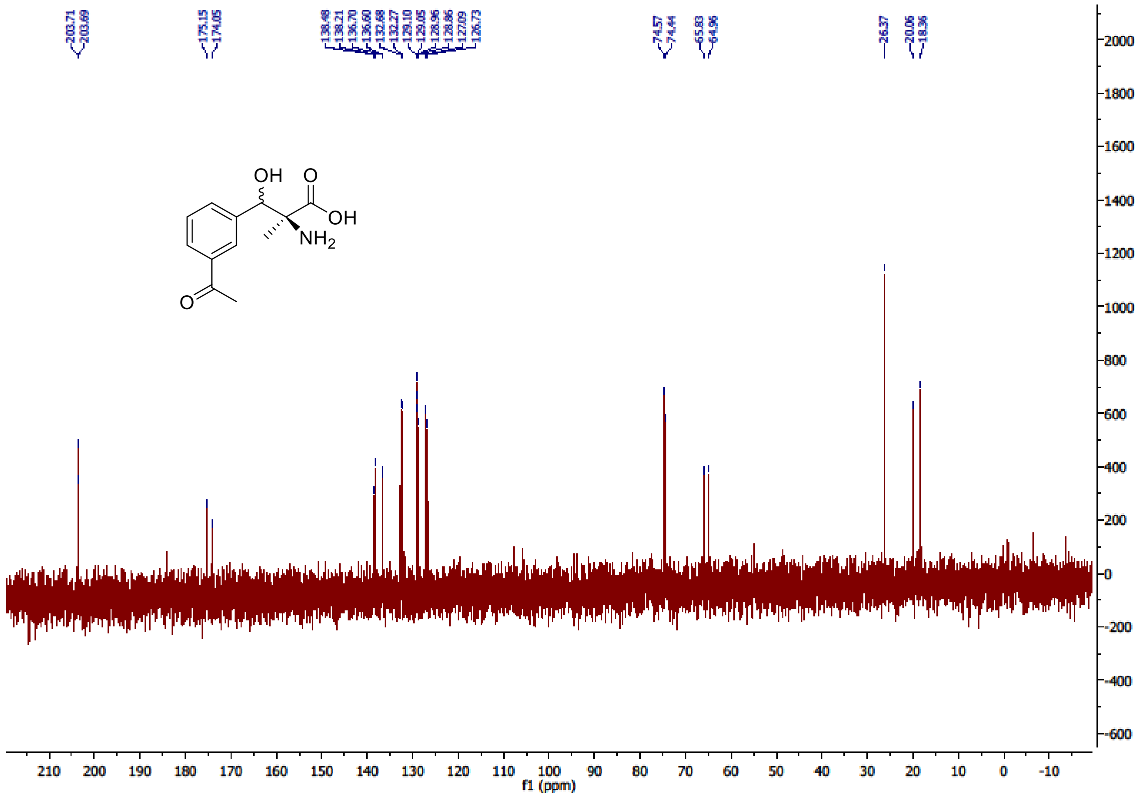

^{1}H-NMR of (2*S*)-**(*p*)-3i** (Table 3, entry 10)

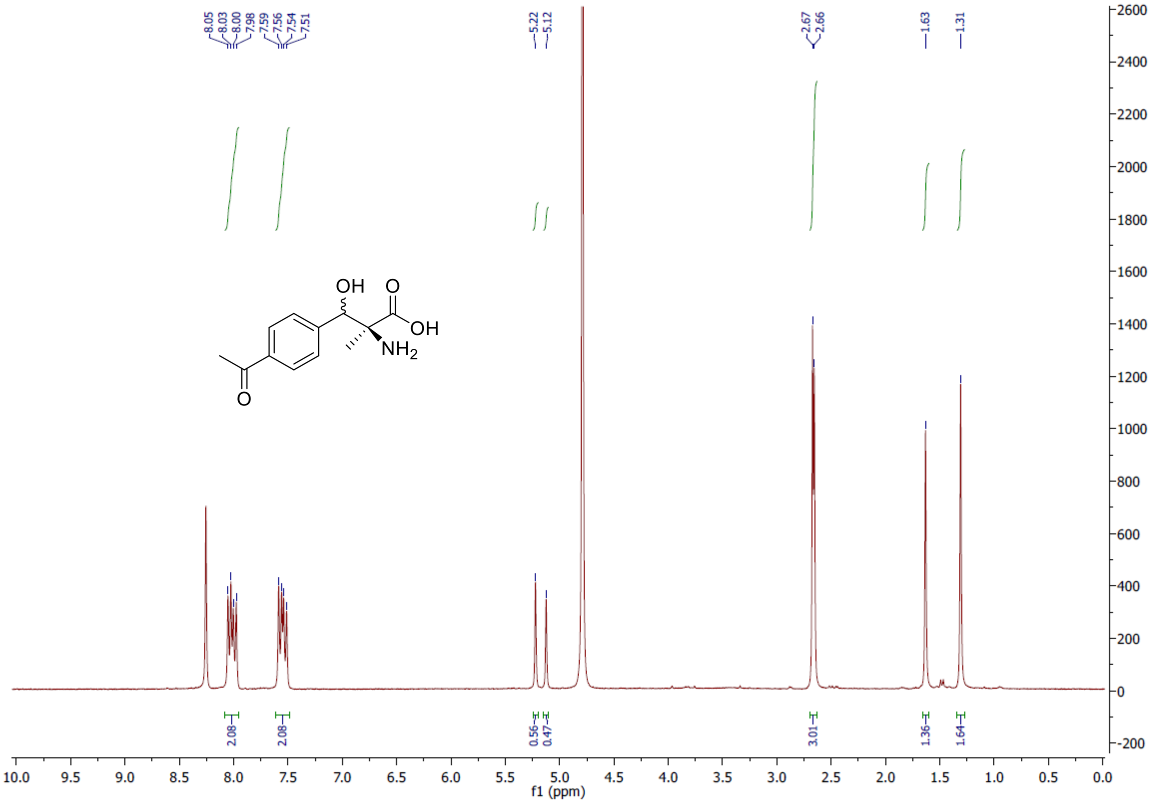

^{13}C-NMR of (2*S*)-**(*p*)-3i** (Table 3, entry 10)

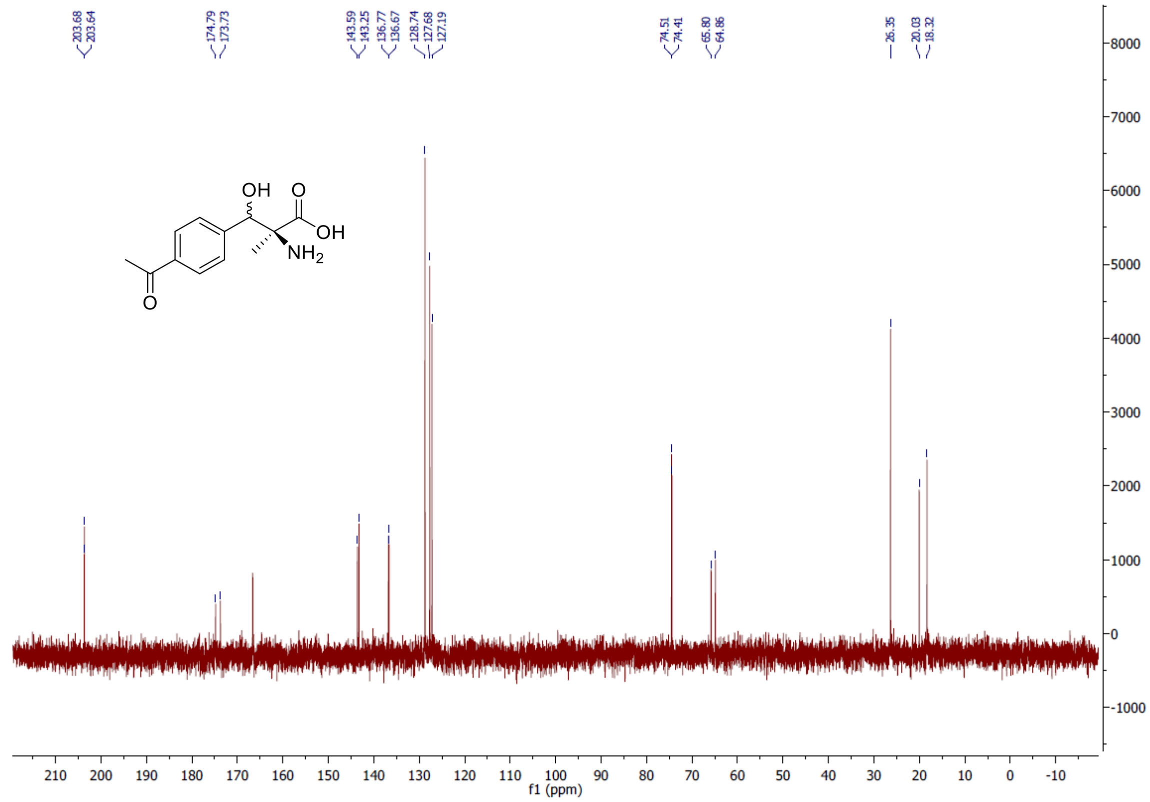

^{1}H-NMR of (2*S*)-**(*m*)-3j** (Table 3, entry 11)

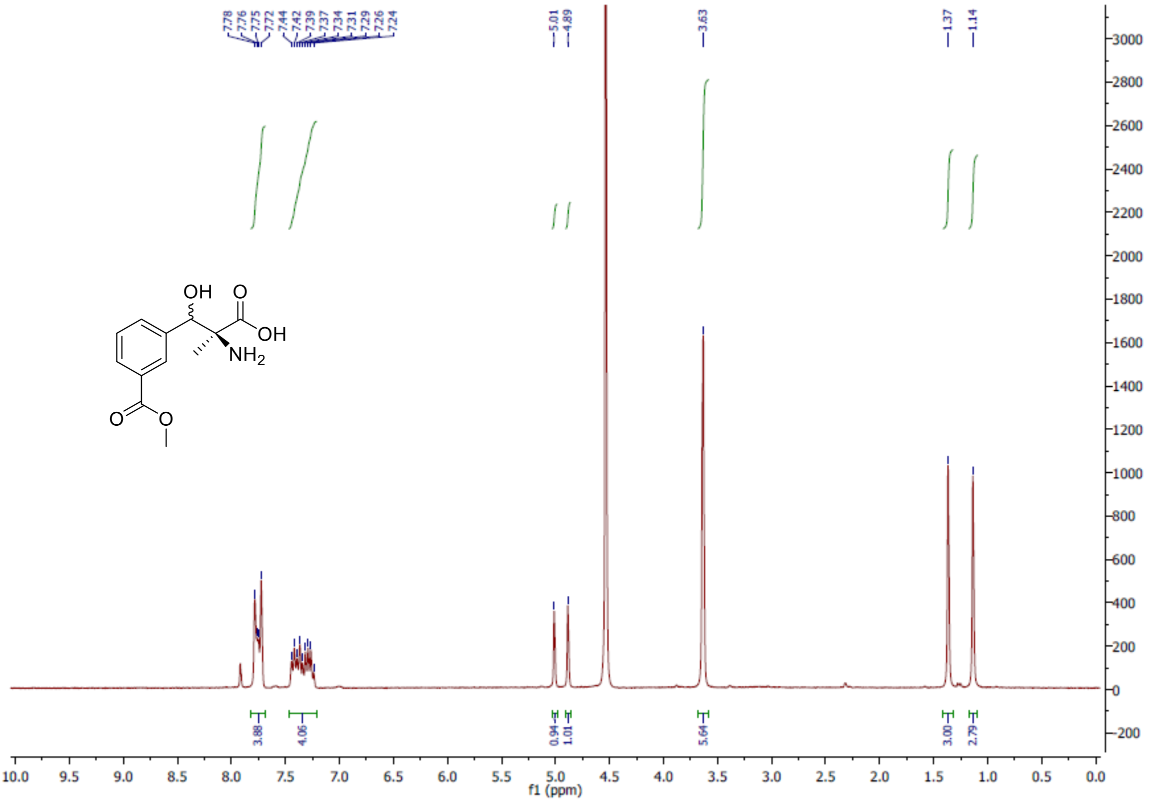

^{13}C-NMR of (2*S*)-**(*m*)-3j** (Table 3, entry 11)

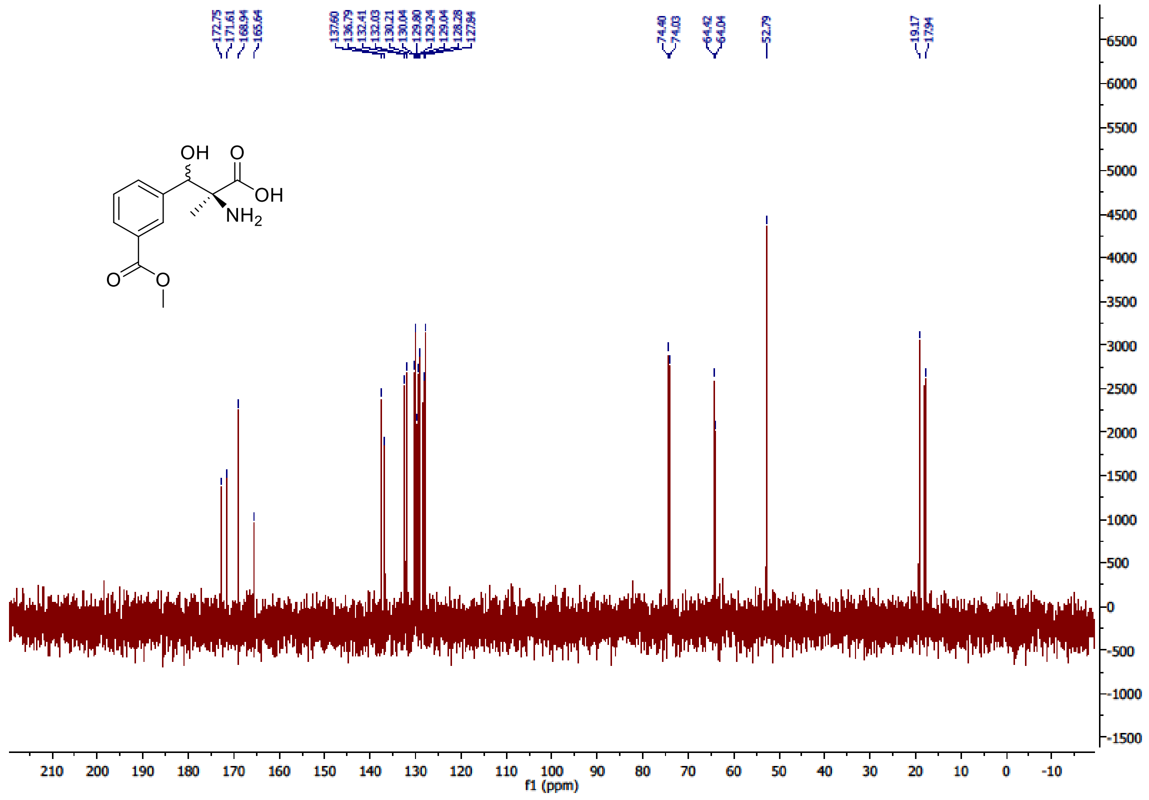

^{1}H-NMR of (2*S*)-**3k** (Table 3, entry 12)

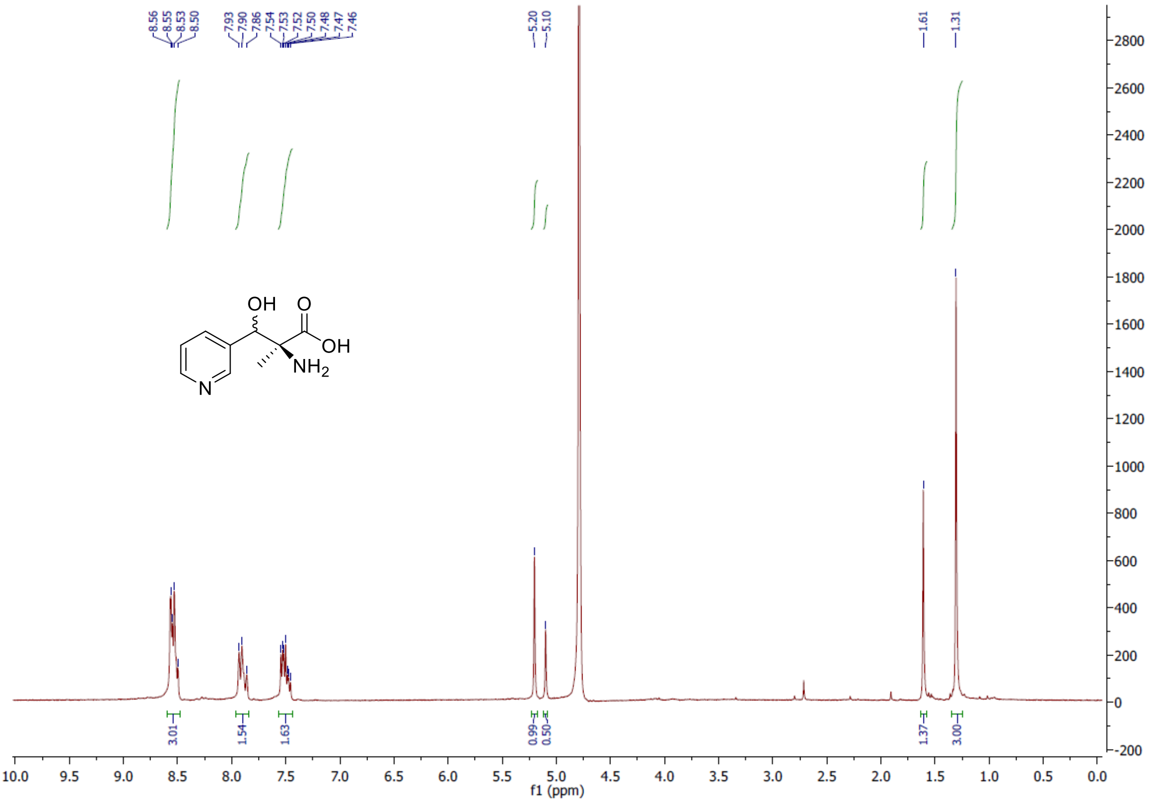

^{13}C-NMR of (2*S*)-**3k** (Table 3, entry 12)

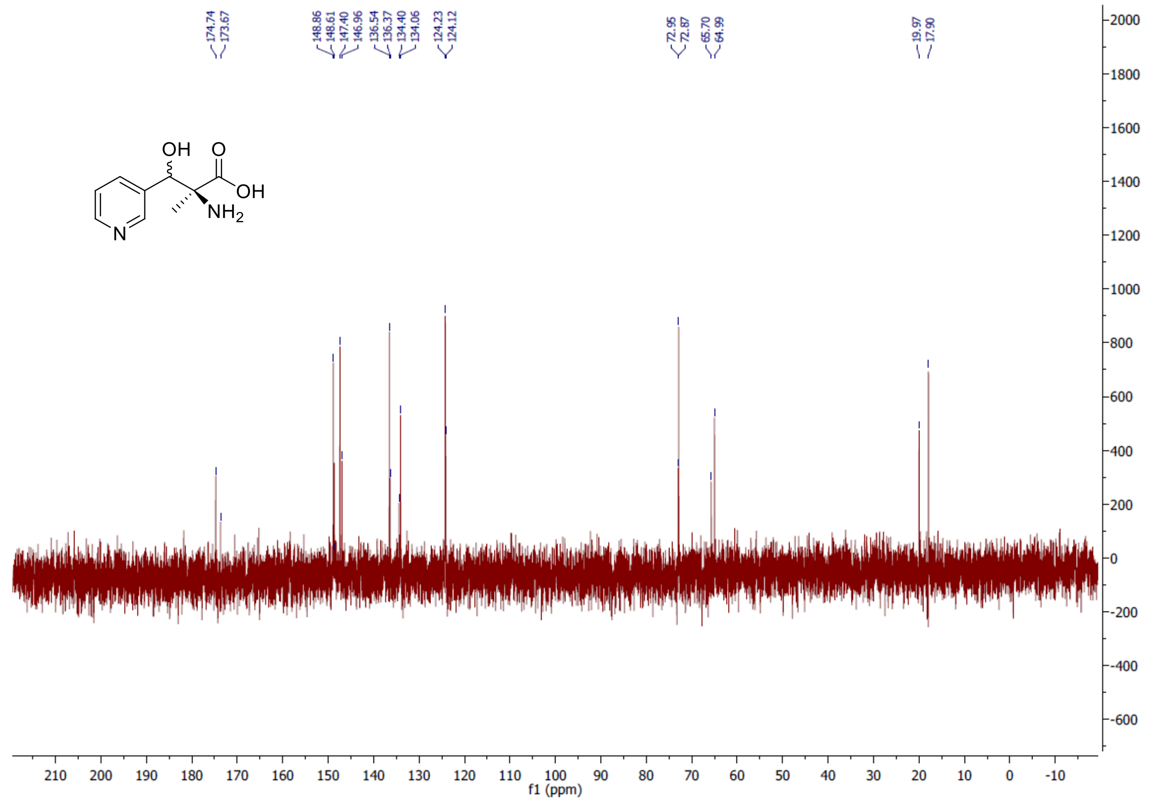

^{1}H-NMR of (2*S*)-**3r** (Table 3, entry 13)

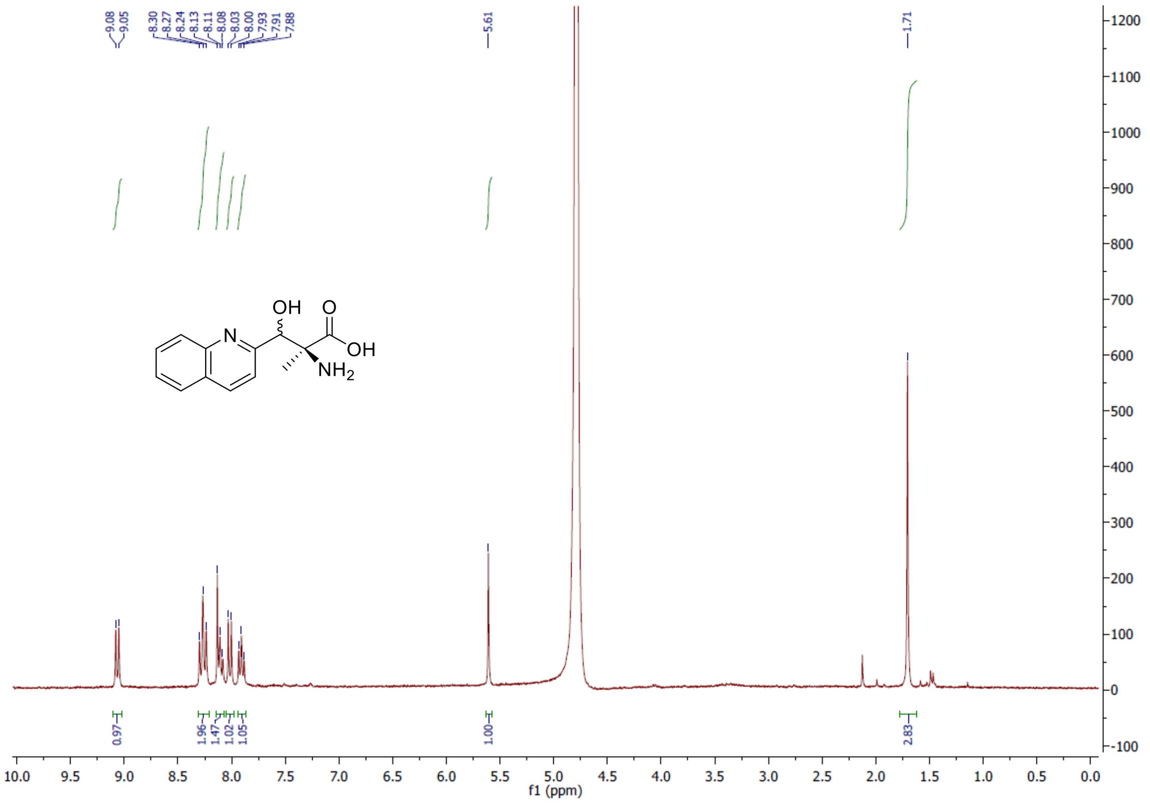

^{13}C-NMR of (2*S*)-**3r** (Table 3, entry 13)

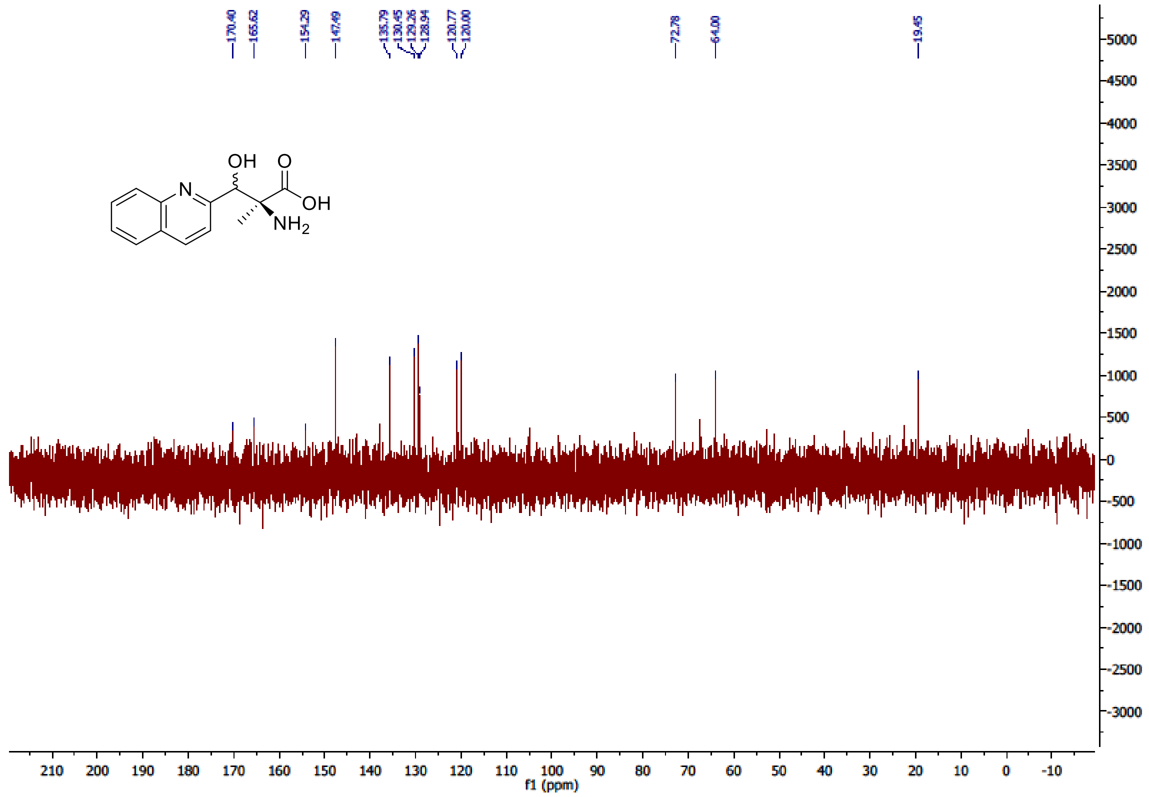

^{1}H-NMR of (2*S*)-**3u** (Table 3, entry 14)

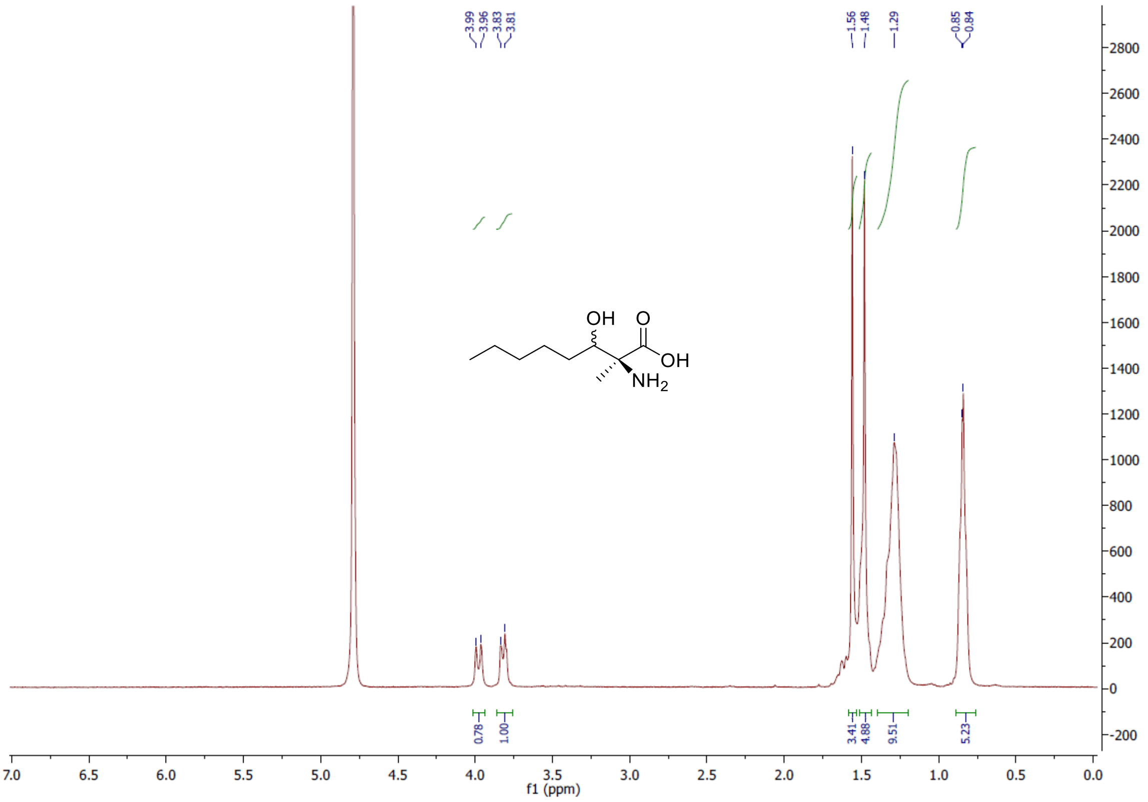

^{13}C-NMR of (2*S*)-**3u** (Table 3, entry 14)

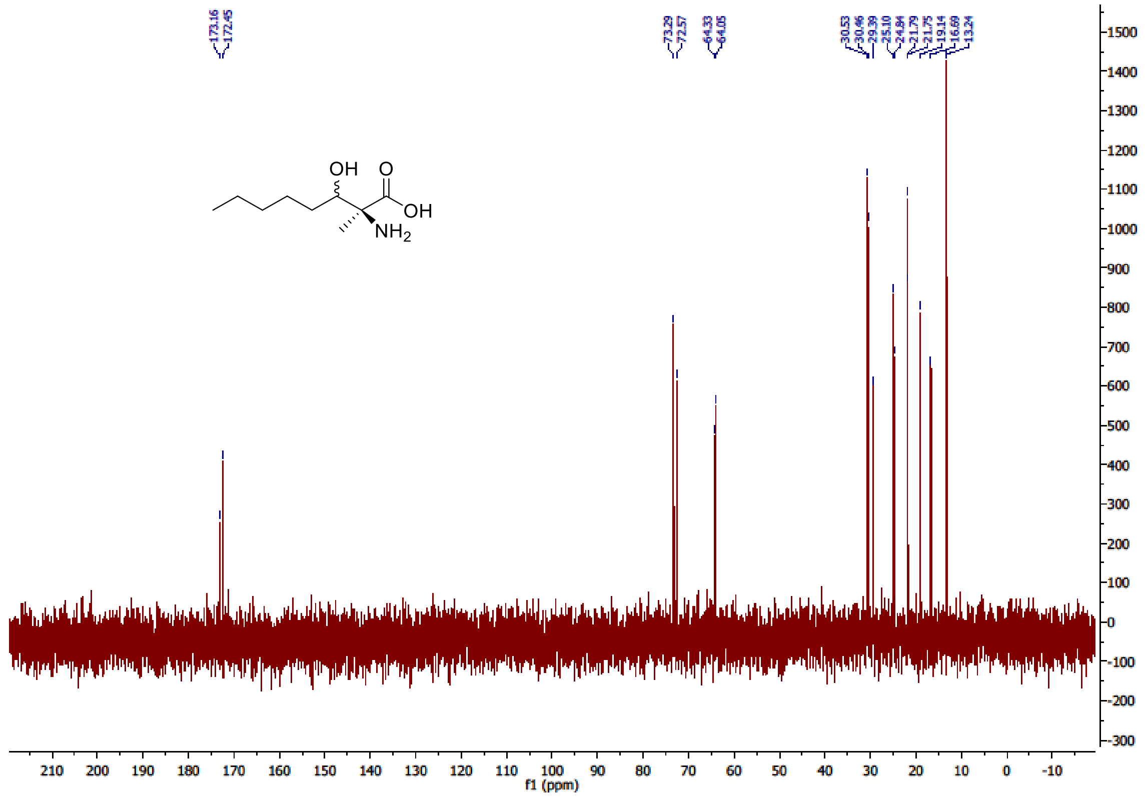

^{1}H-NMR of (2*R*)-**3u** (Table 3, entry 15)

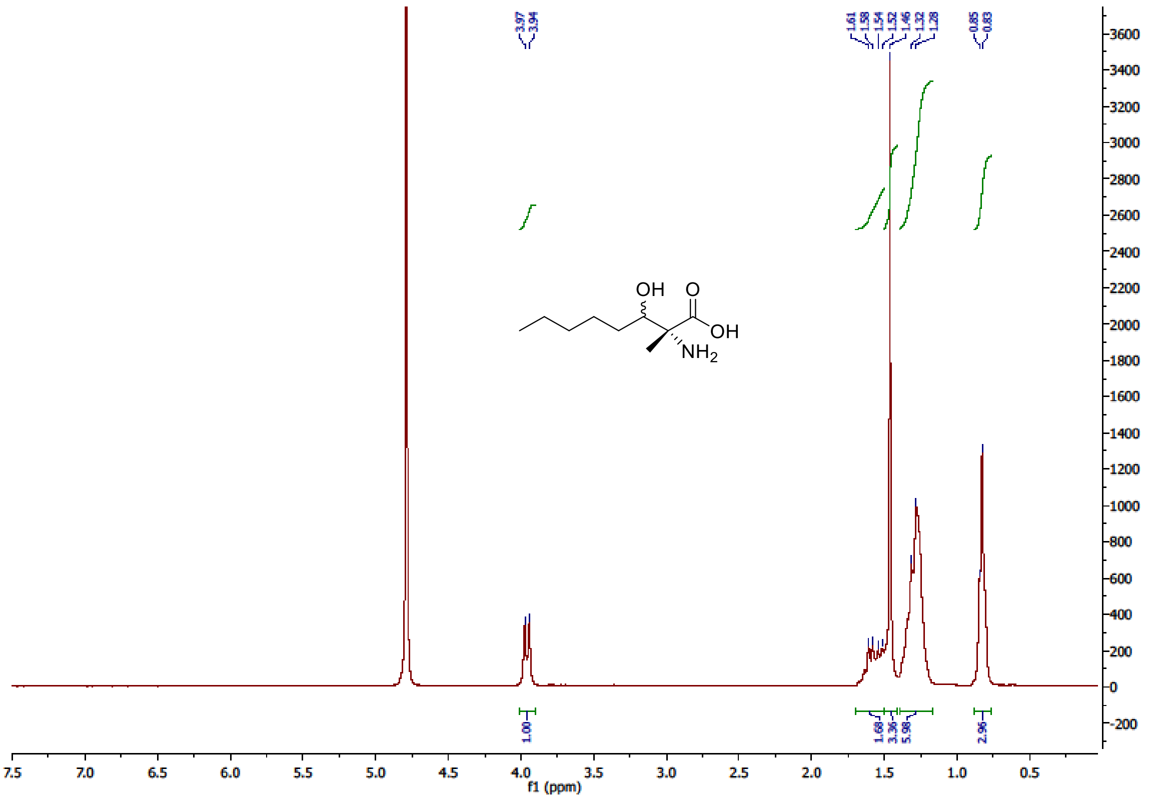

^{13}C-NMR of (2*R*)-**3u** (Table 3, entry 15)

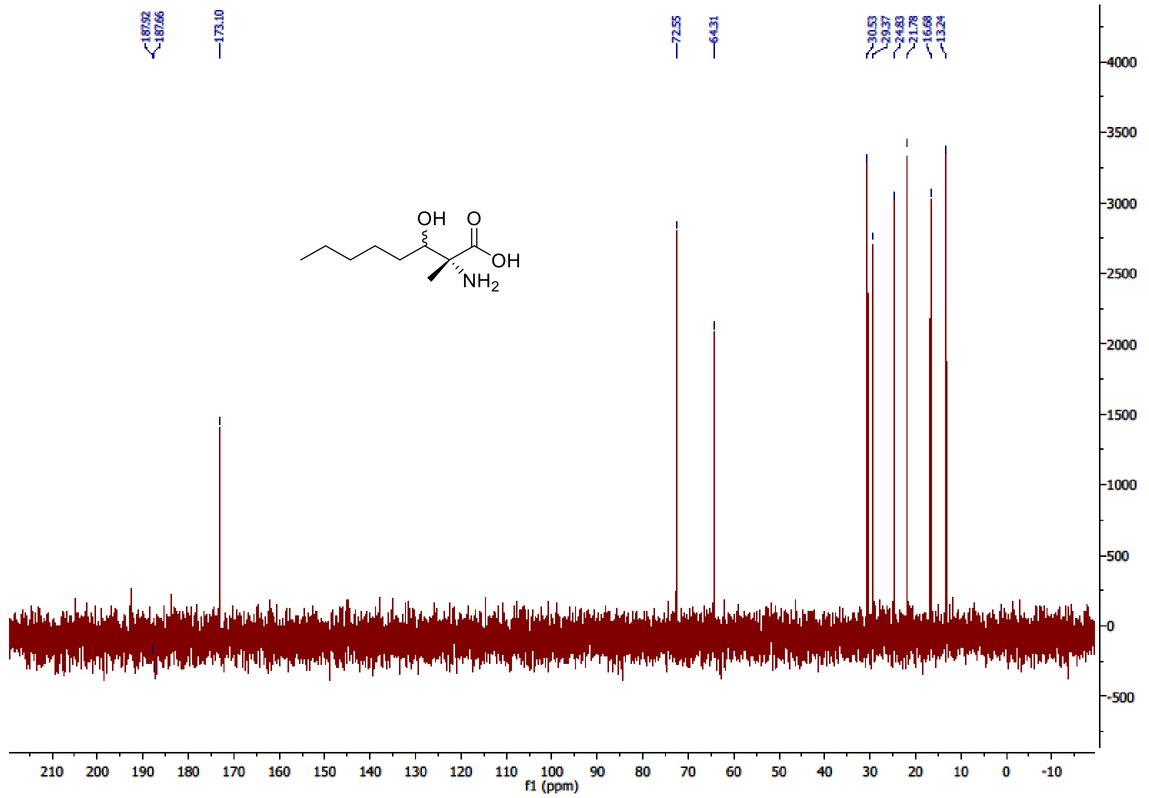

^{1}H-NMR of (2*R*)-**3x** (Table 3, entry 16)

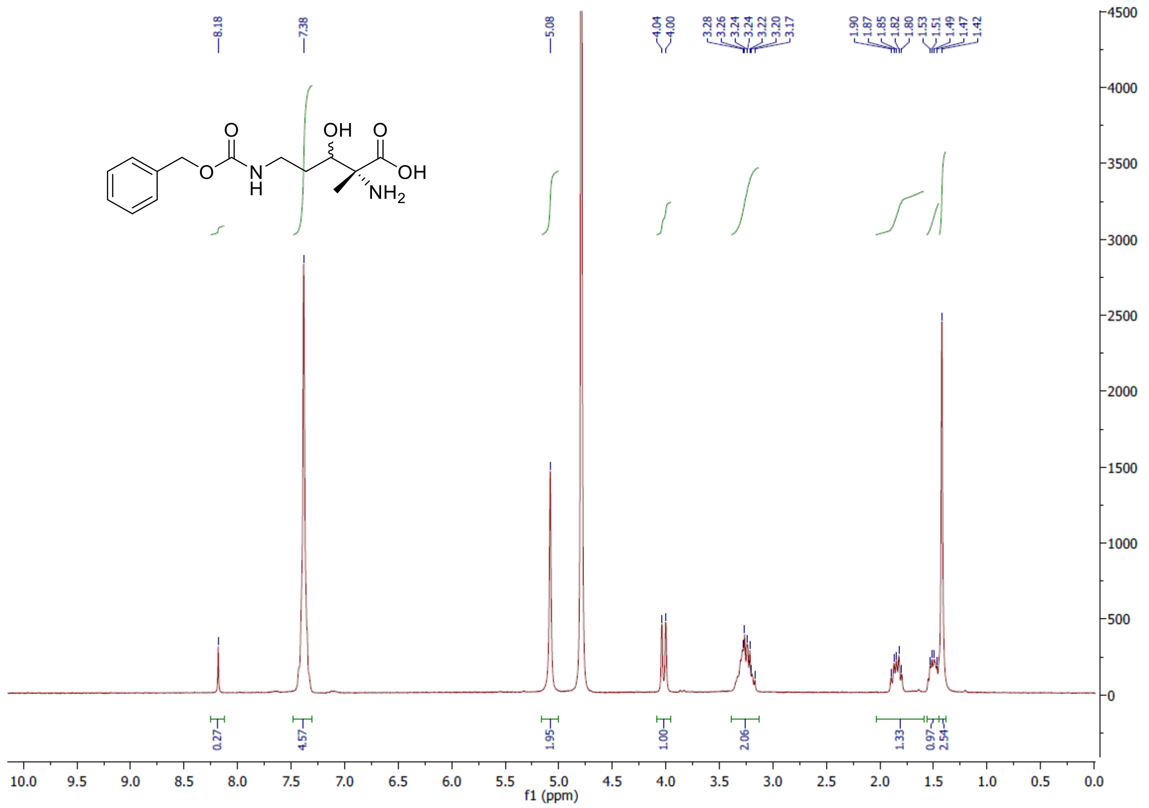

^{13}C-NMR of (2*R*)-**3x** (Table 3, entry 16)

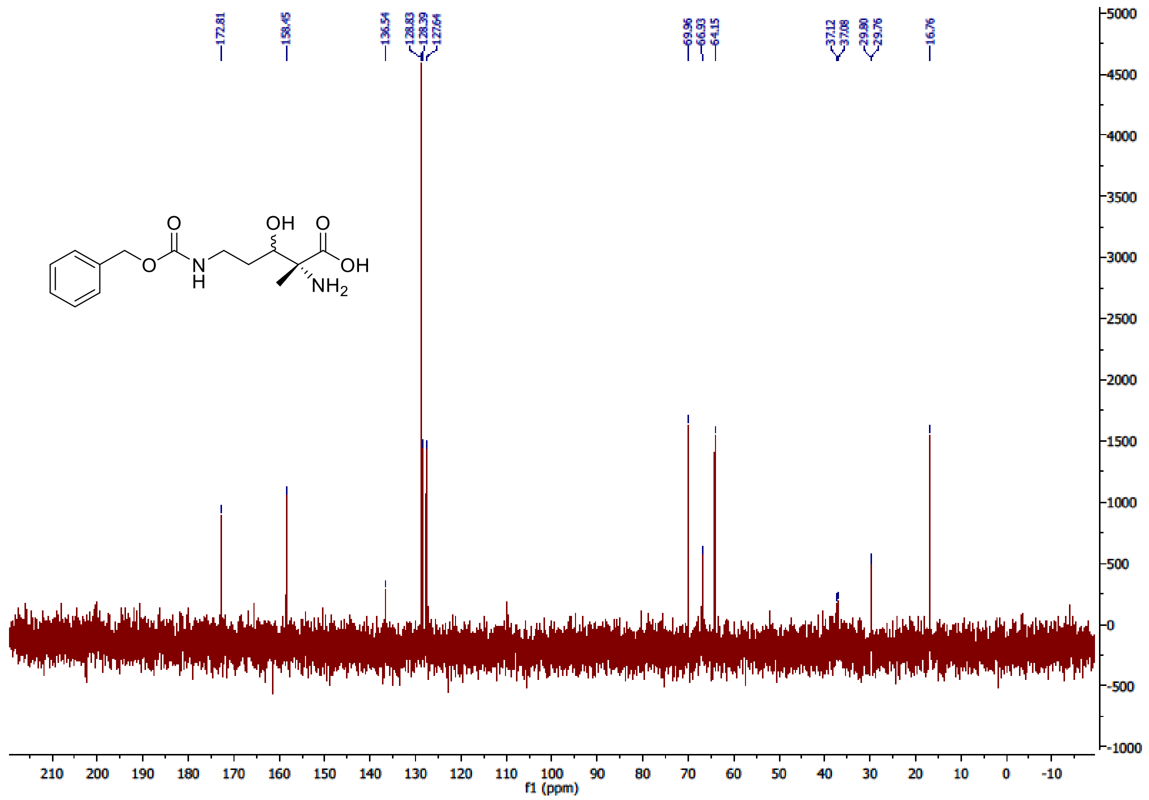

^{1}H-NMR of (*S*)-2-amino-2-methyl-3-phenylpropanoic acid (*S*)-**4a**

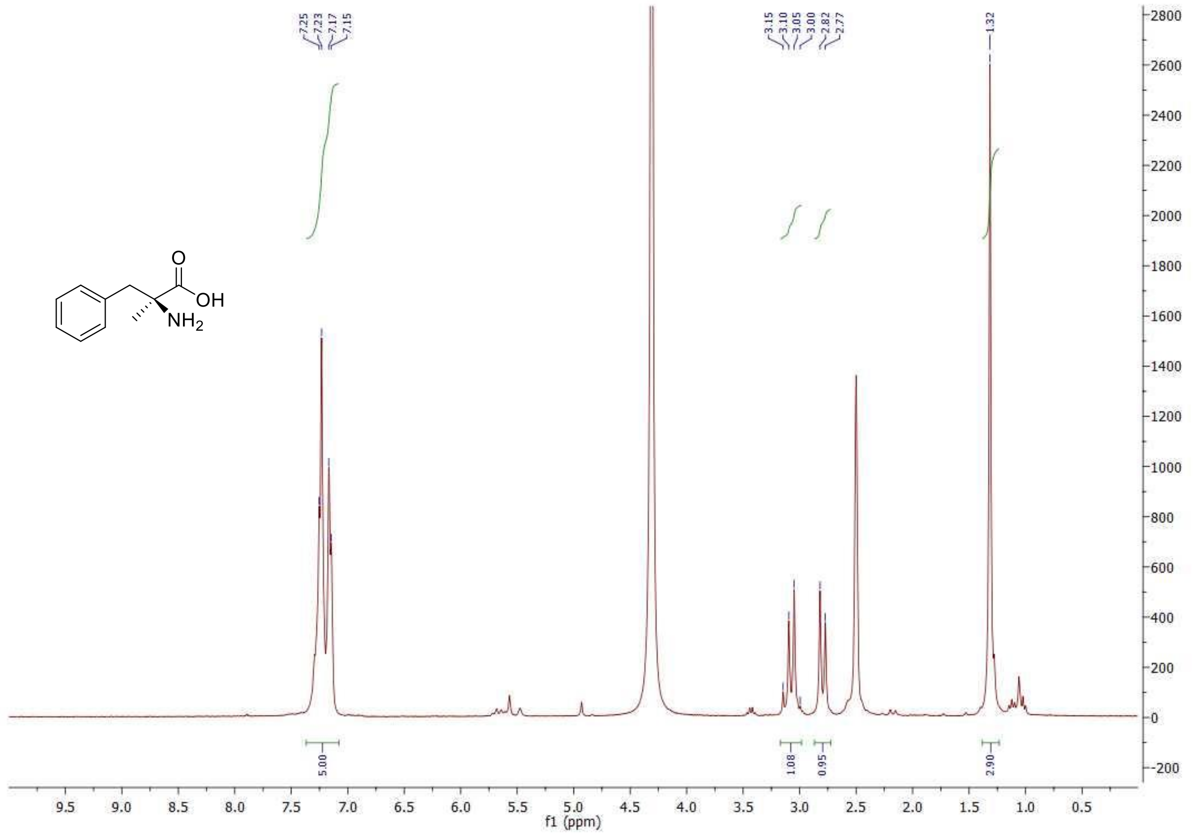

^{13}C-NMR of (*S*)-2-amino-2-methyl-3-phenylpropanoic acid (*S*)-**4a**

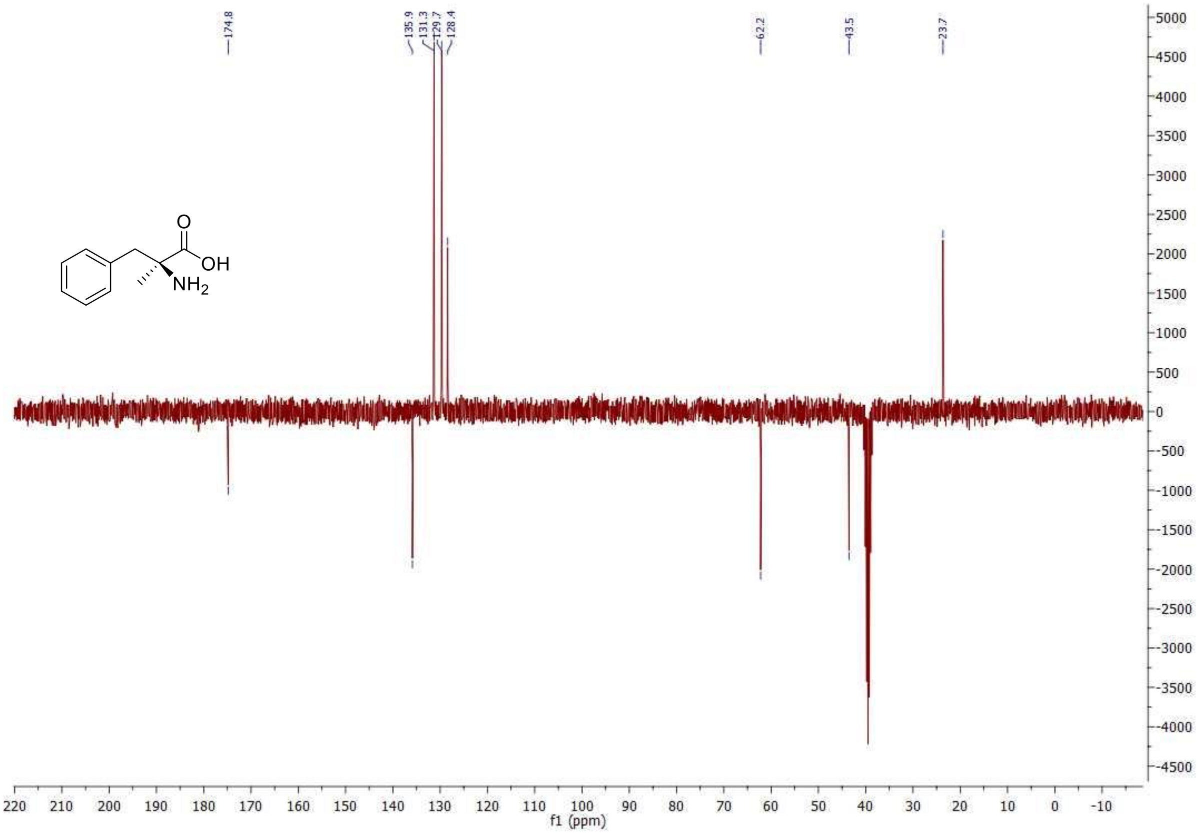

^{1}H-NMR of (2*S*)-3-hydroxy-2-methylindole-2-carboxylic acid (2*S*)-**4b**

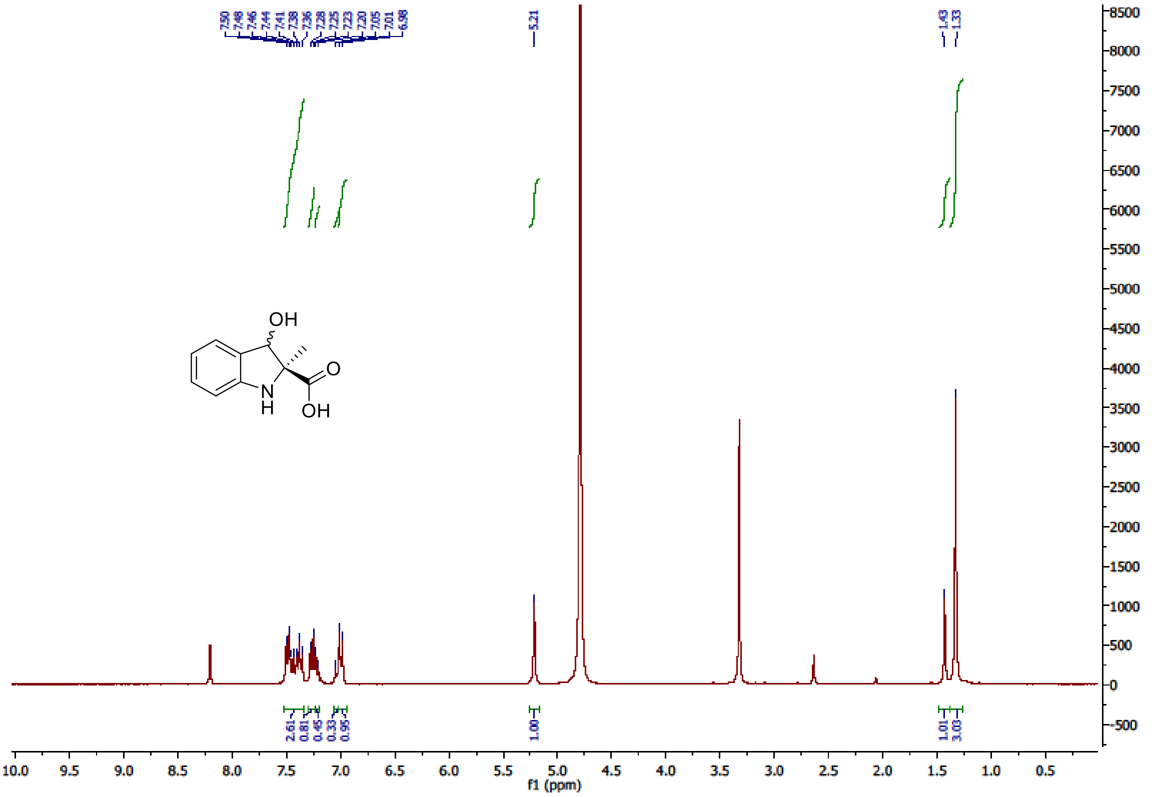

^{13}C-NMR of (2*S*)-3-hydroxy-2-methylindole-2-carboxylic acid (2*S*)-**4b**

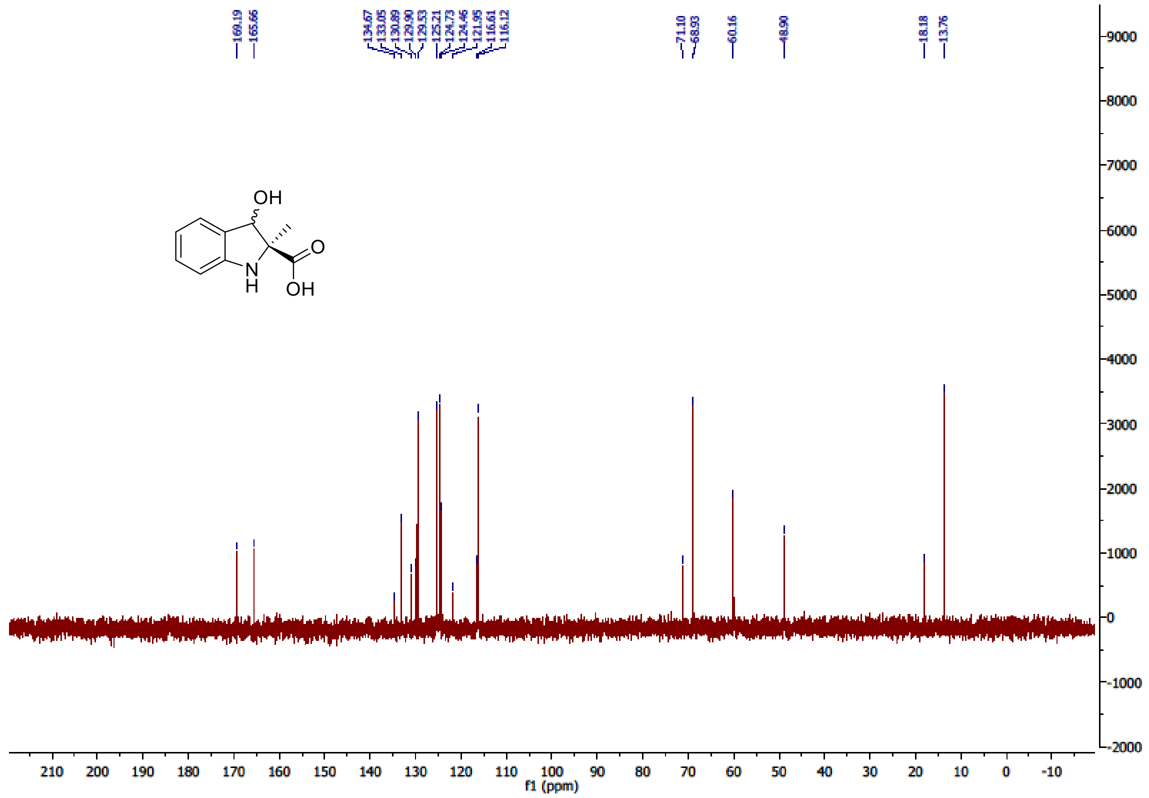

^{1}H-NMR of (S)-2-amino-3-hydroxy-2-methyl-3-(m-tolyl)propanoic acid (S)-**4j**

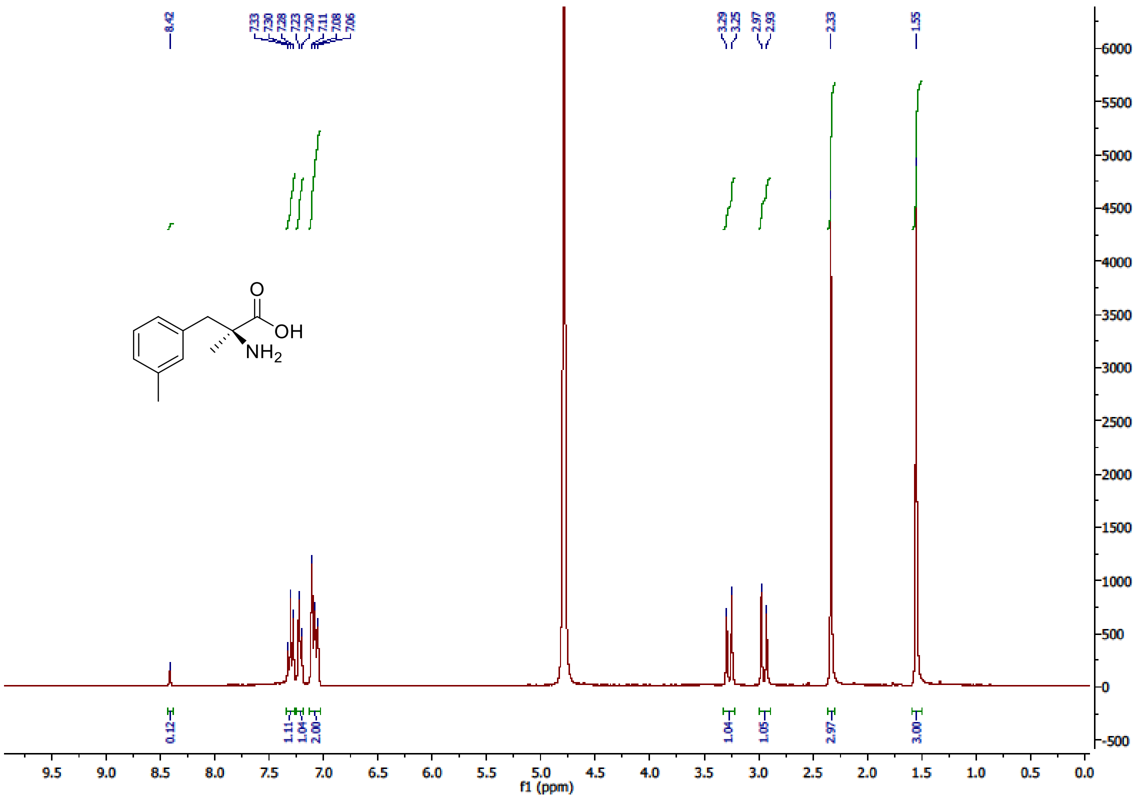

^{13}C-NMR of (S)-2-amino-3-hydroxy-2-methyl-3-(m-tolyl)propanoic acid (S)-**4j**

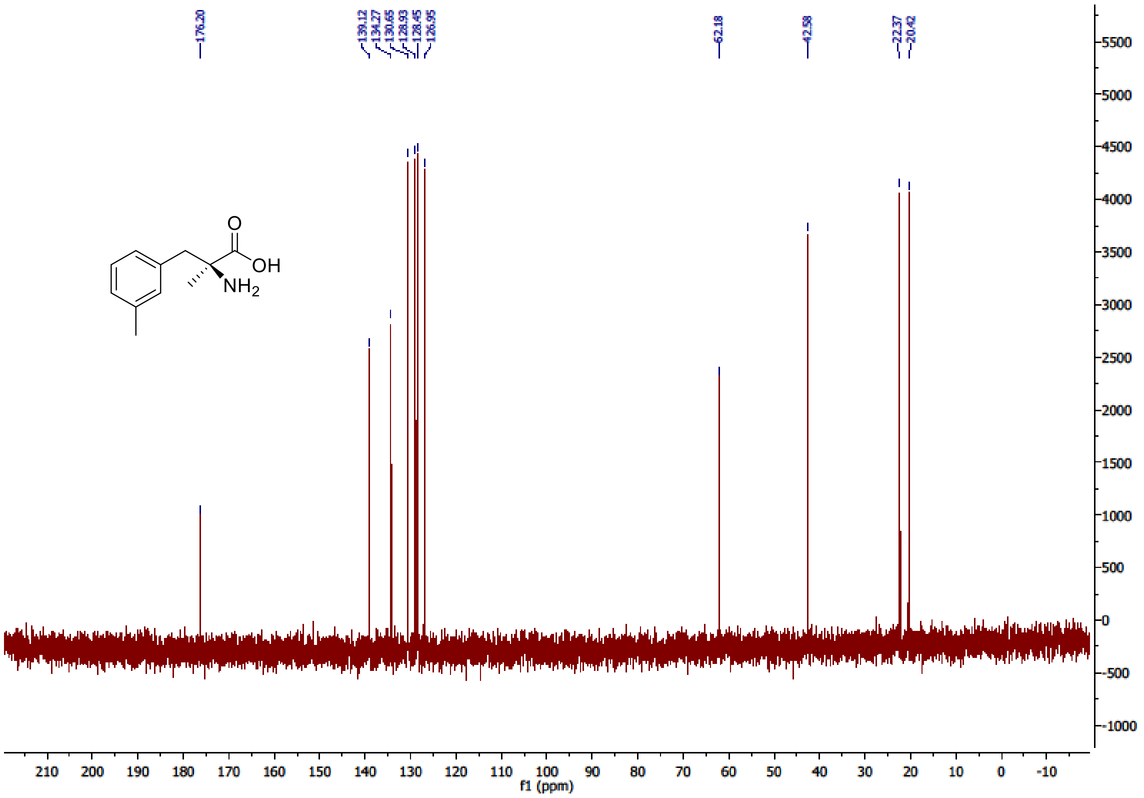

^{1}H-NMR of (*S*)-2-amino-3-(2-chlorophenyl)-2-methylpropanoic acid (*S*)-**6**

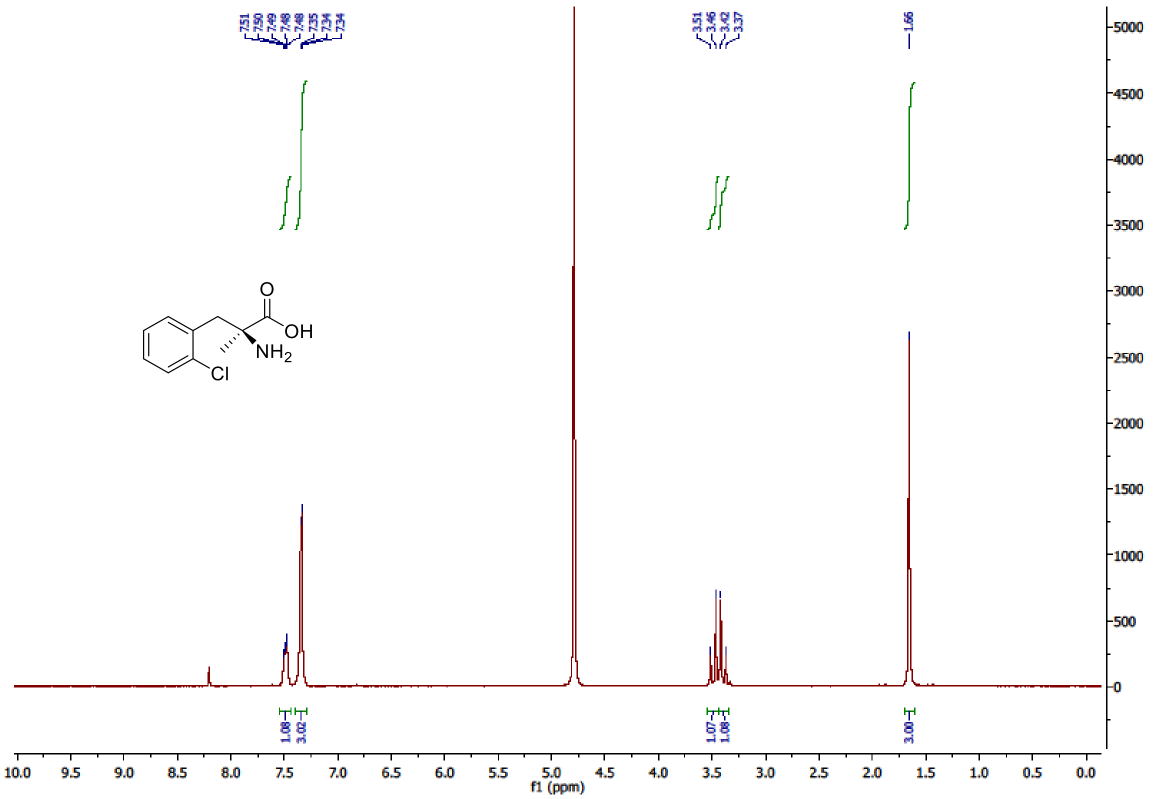

^{13}C-NMR of (*S*)-2-amino-3-(2-chlorophenyl)-2-methylpropanoic acid (*S*)-**6e**

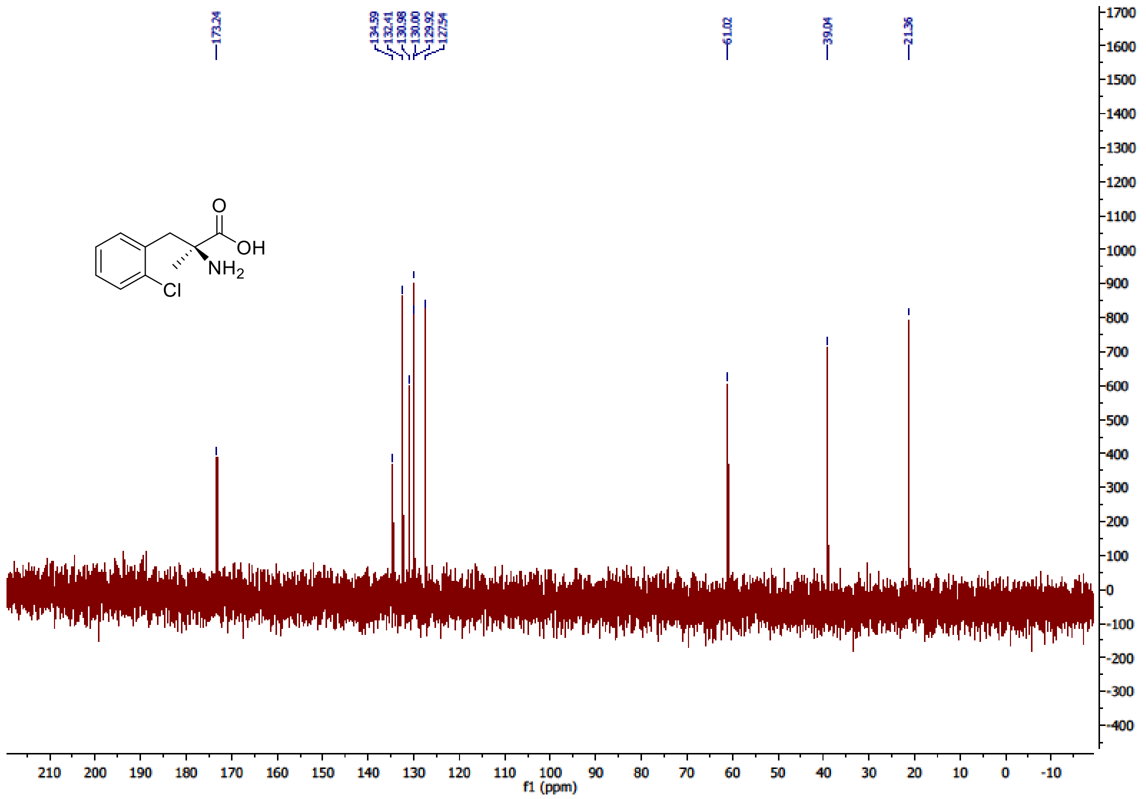

Supplement: Supplementary file 1 — Supplementary [file CCTC-10-3453-s001.pdf]
